# The Notch1 signaling pathway directly modulates the human RANKL-induced osteoclastogenesis

**DOI:** 10.1038/s41598-023-48615-2

**Published:** 2023-12-01

**Authors:** Costanzo Padovano, Salvatore Daniele Bianco, Francesca Sansico, Elisabetta De Santis, Francesco Tamiro, Mattia Colucci, Beatrice Totti, Serena Di Iasio, Gaja Bruno, Patrizio Panelli, Giuseppe Miscio, Tommaso Mazza, Vincenzo Giambra

**Affiliations:** 1 Hematopathology Laboratory, Institute for Stem Cell Biology, Regenerative Medicine and Innovative Therapies (ISBReMIT), Fondazione IRCCS “Casa Sollievo della Sofferenza”, 71013 San Giovanni Rotondo (FG), Italy; 2 Bioinformatics Unit, Fondazione IRCCS Casa Sollievo della Sofferenza, 71013 San Giovanni Rotondo, Italy; 3 Clinical Laboratory Analysis and Transfusional Medicine, Fondazione IRCCS “Casa Sollievo della Sofferenza”, 71013 San Giovanni Rotondo (FG), Italy

**Keywords:** Cell signalling, Next-generation sequencing, Bone development

## Abstract

Notch signaling is an evolutionary conserved pathway with a key role in tissue homeostasis, differentiation and proliferation. It was reported that Notch1 receptor negatively regulates mouse osteoclast development and formation by inhibiting the expression of macrophage colony-stimulating factor in mesenchymal cells. Nonetheless, the involvement of Notch1 pathway in the generation of human osteoclasts is still controversial. Here, we report that the constitutive activation of Notch1 signaling induced a differentiation block in human mononuclear CD14^+^ cells directly isolated from peripheral blood mononuclear cells (PBMCs) upon in vitro stimulation to osteoclasts. Additionally, using a combined approach of single-cell RNA sequencing (scRNA-Seq) simultaneously with a panel of 31 oligo-conjugated antibodies against cell surface markers (AbSeq assay) as well as unsupervised learning methods, we detected four different cell stages of human RANKL-induced osteoclastogenesis after 5 days in which Notch1 signaling enforces the cell expansion of specific subsets. These cell populations were characterized by distinct gene expression and immunophenotypic profiles and active Notch1, JAK/STAT and WNT signaling pathways. Furthermore, cell–cell communication analyses revealed extrinsic modulators of osteoclast progenitors including the IL7/IL7R and WNT5a/RYK axes. Interestingly, we also report that Interleukin-7 receptor (IL7R) was a downstream effector of Notch1 pathway and that Notch1 and IL7R interplay promoted cell expansion of human RANKL-induced osteoclast progenitors. Taken together, these findings underline a novel cell pattern of human osteoclastogenesis, outlining the key role of Notch1 and IL-7R signaling pathways.

## Introduction

Bone homeostasis is a dynamic process characterized by the balanced activity between osteoblasts, which are involved in the bone formation and osteoclasts, responsible for bone resorption^1^. Osteoclasts are giant multinucleated bone-resorbing cells that originate from the fusion of hematopoietic progenitors of monocytes and macrophages lineage^2^. The differentiation process of monocyte to osteoclasts, namely known as osteoclastogenesis, is a multi-stage process induced by the interactions between osteoclast progenitors and stromal cells derived from mesenchymal stem cells within the bone marrow^3,4^. Specifically, osteoclast progenitors can be differentiated in mature cells upon stimulation induced by two critical cytokines, the receptor activator of NF-κB ligand (RANKL) and the macrophage-colony stimulating factor (M-CSF)^5–7^. In the bone marrow, the molecular cross-talk between RANKL-RANK and RANKL-osteoprotegerin (OPG) interaction system acts as dominant final mediator to fine-tunes bone homeostasis in normal physiology and disease condition^8^. Interestingly, human osteoclasts can also be generated from cell precursors directly isolated from peripheral blood mononuclear cells (PBMCs) using an in vitro RANKL-dependent culture system^9,10^.

In physiological conditions, the osteoclast precursors are the CD14^++^CD16^−^ classical monocytes, whereas in inflammatory conditions the CD14^++^CD16^+^ intermediate monocytes can also differentiate into osteoclasts with an increased ability of bone resorption^11^. Recently, cell subsets, double positive for CD11b and CD14 surface markers have also identified as osteoclast progenitors along with a second CD14^−^CD11^blo^CD115^+^ cell population with high monocytic progenitor activity and the capacity of producing osteoclast-like cells^11^. Interestingly, adaptive immune cells, such as T-cell subsets, also modulate this process. During tissue inflammation or inflammatory responses, activated T cells are indeed one of the major sources of RANKL and promote the differentiation of osteoclast precursors to more mature cell subsets^12,13^.

The role of Notch signaling on human osteoclastogenesis and osteoclast function is still unclear. Nonetheless, it was reported that in mice, Notch receptors have a direct impact on osteoclast precursors and indirectly enhance the ability of osteoblast lineage cells to stimulate osteoclastogenesis^14–18^. Notch family members are parts of an evolutionary conserved intercellular signaling pathway that regulates a variety of developmental processes by controlling cell fate decisions during skeletal development as well as during adult life and cancer^19^. Notch proteins are a family of type-1 transmembrane proteins which are evolutionary conserved and regulate different fundamental cellular processes through a signal transduction mechanism^20^. In mammals, four Notch receptors (Notch1 to -4) and five ligands (Delta1, -3, and -4 and Jagged1 and -2) were identified to mediate the canonical Notch signaling pathway^20^. The Notch extracellular domain (NECD) mediates interactions with DSL (Delta, Serrate, LAG-2) family ligands resulting with a receptor proteolysis induced by the ligand to unmask the protected transmembrane S2/S3 sites for cleavage by ADAMs and γ-Secretase^20^. The release of Notch intracellular domain (NICD) into the nucleus allows the association with proteins coactivators and the formation of the transcription complex^21^. This complex is responsible for the transcription of NOTCH target genes, including those of HES (hairy and enhancer of split) and the HES-related transcription factor HEY^22^. Interestingly, it was reported that Notch1 receptor negatively regulates mouse osteoclast development and formation by inhibiting the expression of macrophage colony-stimulating factor in mesenchymal cells inhibits^15^, contrarily to Notch2/Delta-like 1 axis^18,23^.

In this study, to focus on the suppressive role of Notch1 signaling on human osteoclastogenesis, human CD14^+^CD16^−^ monocytes were transduced with lentivectors encoding a constitutive active NOTCH1-ΔE isoform of Notch1 receptor and subsequently tracked over time in culture after in vitro RANKL stimulation. We found the constitutive activation of Notch1 pathway in osteoclast precursor promoted cell differentiation blocks at the early stages of osteoclastogenesis. Furthermore, using a combined approach of gene expression data at the single-cell level (scRNA-Seq), simultaneously with a panel of oligo-conjugated antibodies (AbSeq assay), we characterized four different cell stages of human osteoclastogenesis. Finally, we also point out that the interleukin 7 receptor (IL7R) acted as a downstream effector of Notch1 signaling pathways in osteoclast precursors. These observations highlight the functional suppressive role of Notch1 signaling in human osteoclastogenesis and may provide novel therapeutic opportunities and strategies for osteoclast-related diseases.

## Materials and methods

### Isolation of peripheral blood mononuclear cells (PBMCs)

After appropriate institutional approvals (“Fondazione IRCCS Casa Sollievo della Sofferenza” Research Ethics Boards, Prot. N.155/CE, ICF: V1.0_16 Mag 18), blood specimens of healthy donors were collected at the “Casa Sollievo della Sofferenza” Hospital in Southern Italy. Informed consent was obtained from all the participants. All methods were carried out in accordance with the Declaration of Helsinki. PBMCs were isolated from whole blood using Ficoll-Paque. Briefly, 6 ml of EDTA-anticoagulated blood was diluted with an equal volume of phosphate-buffered saline, pH 7.4 (PBS), containing 0.05 M ethylenediaminetetraacetic acid (EDTA; Invitrogen). 12 ml of diluted blood was layered over 24 ml of the Ficoll-Paque PLUS (GE Healthcare). Gradients were centrifuged at 400×*g* for 30 min at room temperature in a swinging-bucket rotor with no brake or acceleration. The cell interface was carefully removed by pipetting and washed with PBS-EDTA by centrifugation at 250×*g* for 10 min. PBMC pellets were suspended in ammonium-chloride solution (Stemcell Technologies) and incubated for 10 min at room temperature on mixing platform in order to lyse contaminating red blood cells. Isolated PBMCs were finally washed with PBS-EDTA and then cryopreserved in liquid nitrogen in fetal calf serum (FCS; Invitrogen) containing 10% dimethylsulfoxide (DMSO; ThermoFisher Scientific) and stored until required for downstream analyses.

### Isolation of human monocytes and cell stimulation

Untouched human monocytes were purified from PBMCs by magnetic depletion beads and an antibody mix containing biotinylated mouse IgG antibodies for CD3, CD7, CD16 (specific for CD16a and CD16b), CD19, CD56, CDw123 and CD235a (Glycophorin A) for the removal of non-monocytes (NK cells, B and T cells, T cells, dendritic cells, granulocytes, erythrocytes, and macrophages), according to the manufacturer’s recommendations (Dynabeads™ Untouched™ Human Monocytes Kit, cat. 11350D, Invitrogen). Afterwards, purified CD14^+^CD16^−^ monocytes or total PBMCs were cultured in RPMI1640 medium supplemented with 10% fetal bovine serum (FBS), 1 mM sodium pyruvate, 2 mM l-glutamine, 100 units/ml penicillin, 100 μg/ml streptomycin (ThermoFisher), seeded at 2 × 10^6^ cells/ml and supplemented with recombinant human sRANKL (50 ng/ml, cat. 310-01, Peprotech), M-CSF (25 ng/ml, cat. 300-25, Peprotech), TGF-β1 (5 ng/ml, cat. 100-21C, Peprotech) and dexamethasone (1 μM, cat. D4902, Sigma) to induce osteoclastogenesis as described previously^9^. Recombinant human IL-7 protein (cat. 207-IL, Peprotech) was also employed. Neutralizing antibody against IL-7R (CD127 Monoclonal Antibody (A7R34), cat. #14-1271-82, ThermoFisher) or mouse IgG1 isotype (Mouse IgG1 kappa Isotype Control (P3.6.2.8.1), cat. #13-4714-85, ThermoFisher) as mock control was used at the concentration of 1 μg/ml.

### Acid phosphatase, leukocyte (TRAP) assay

Adherent cell cultures were fixed by immersing in fixative solution and subsequently stained, according to the manufacturer's recommendations (acid phosphatase, leukocyte (TRAP) kit, cat. 387A, Sigma). Afterwards, the stained cells were identified and visualized using the Eclipse TE300 Inverted Microscope.

### Single cell RNA-sequencing (scRNA-Seq) and Ab-sequencing (AbSeq)

Whole transcriptome analyses at single cell level and profiling of oligoconjugated antibodies were performed on FACS-sorted GFP^+^ alive cells using the BD Rhapsody Single-Cell Analysis System (BD, Biosciences). Specifically, human CD14^+^CD16^−^ monocytes were isolated from PBMCs and transduced with lentivectors encoding the active NOTCH1-ΔE isoform or empty vector as control. Transduced GFP^+^ cells were in vitro RANKL stimulated and expanded for 5 days as indicated above and subsequently purified by FACS-sorting. Isolated cells from each sample were labeled with the BD Single-Cell Multiplexing Kit (cat. 633781, BD Biosciences) as well as 31 BD AbSeq Ab-Oligos for 1 h (Table S1), following the manufacturer’s protocol.

## Results

### The constitutive activation of Notch1 signaling in human osteoclast precursors blocks the in vitro RANKL-induced osteoclastogenesis

**Figure 1:**
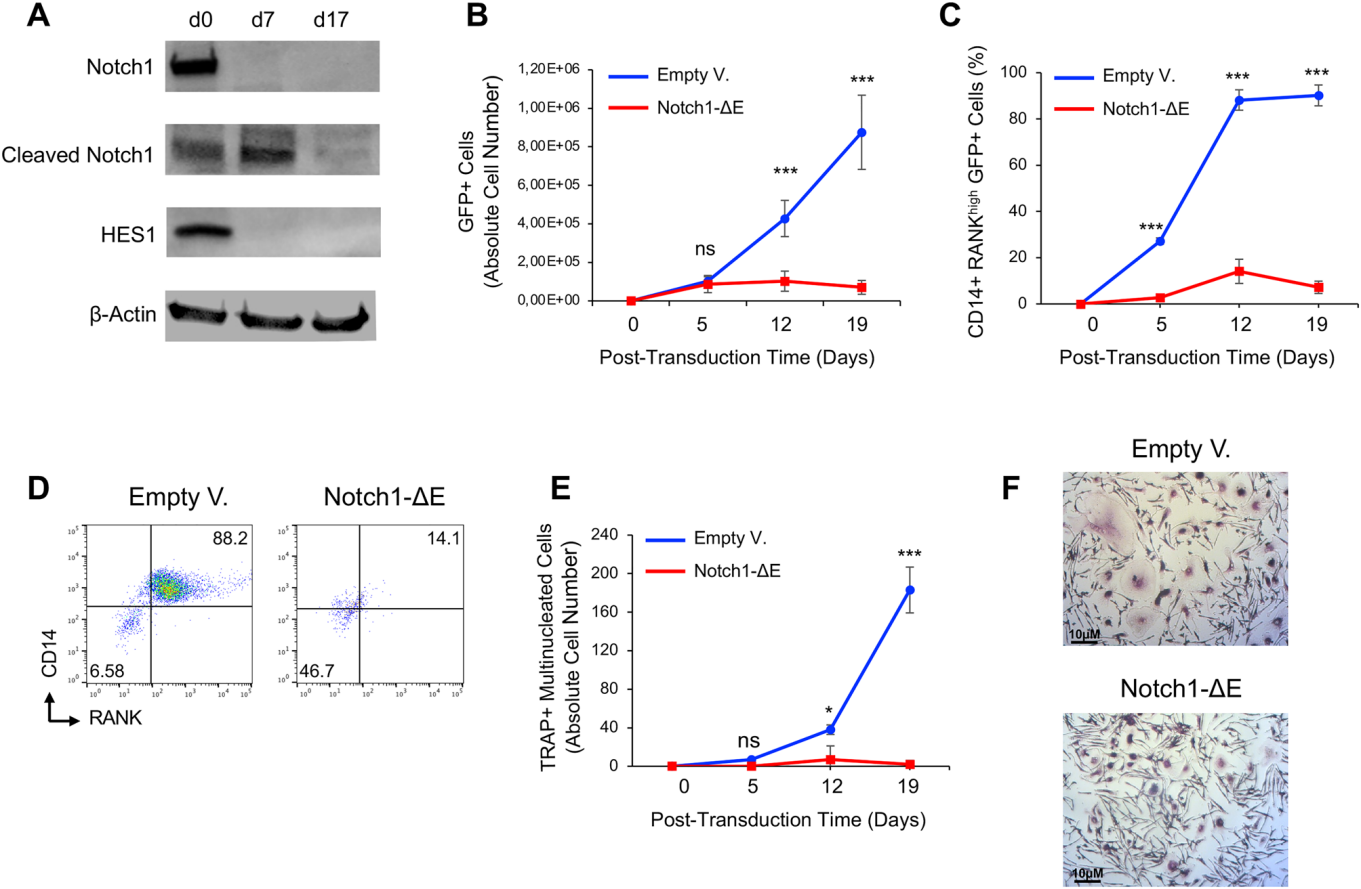
Constitutive activation of Notch1 signaling in human osteoclast precursors suppresses in vitro RANKL-induced osteoclastogenesis. (**A**) Western blot analysis of total and cleaved intracellular NOTCH1, HES1 and β-Actin as a loading control in total cell lysates of human CD14^+^CD16^−^ monocytes at day 0 and after in vitro growth in serum-containing growth media, supplemented with RANKL (50 ng/ml), M-CSF (25 ng/ml), TGF-β1(5 ng/ml) and dexamethasone (1 μM) and seeded at 2 × 10^6^ cells/ml for 7 and 17 days. The blots were cut prior to hybridisation with antibodies. The uncropped version of the western blots is reported in Fig. S19. (**B**,**C**) Flow cytometric analysis of abundance of total GFP^+^ cells (**B**) and CD14^+^RANK^high^GFP^+^ cell fraction (**C**) in human CD14^+^CD16^−^ monocytes, isolated from peripheral blood mononuclear cells (PBMC) and transduced with lentivectors encoding the active NOTCH1-ΔE isoform or empty vector as control. Abundance of transduced total GFP^+^ cells (**B**) and CD14^+^RANK^high^GFP^+^ cell fraction (**C**) was tracked over time in culture after in vitro RANKL stimulation at the indicated time points by flow cytometry. GFP^+^ alive cells were discriminated using the LIVE/DEAD™ Fixable Near-IR stain. Means ± SD fractions of the initial transduction value are plotted for experiments performed in biological triplicates using PBMCs derived from different healthy donors. ***p < 0.001 (Student’s *t*-test); *NS* not significant. (**D**) Flow cytometry plots of NOTCH1-ΔE or empty vector transduced cells after 12 days of in vitro RANKL stimulation, showing the protein expression of CD14 and RANK cell receptors. (**E**) Absolute number of TRAP-positive multinucleated cells, derived from human CD14^+^CD16^−^ monocytes, isolated from PBMC, lentivirally transduced with the active NOTCH1-ΔE isoform or empty lentivectors as control and subsequently in vitro stimulated as described above. Number of TRAP-positive multinucleated cells was quantified microscopically over time in culture as indicated. Means ± SD values are plotted for experiments performed in biological triplicates. *p < 0.05; ***p < 0.001 (Student’s *t*-test); *NS* not significant. (**F**) Microphotographs of TRAP-positive multinucleated cells, transduced as indicated after 19 days of in vitro RANKL stimulation.

To address the role of Notch1 signaling on osteoclast differentiation, we assessed the protein levels of total and cleaved intracellular NOTCH1 in human CD14^+^CD16^−^ monocytes isolated from peripheral blood mononuclear cells (PBMCs) and subsequently, cultured in serum-containing growth media, supplemented with RANKL (50 ng/ml), M-CSF (25 ng/ml), TGF-β1 (5 ng/ml) and dexamethasone (1 μM) to induce in vitro osteoclastogenesis as previously described^9^. We found that the expression levels of total NOTCH1 receptor is absent after 7 days and subsequently the active intracellular NOTCH1 proteins decreased in RANKL-induced cells after 17 days of in vitro growth (Fig. 1A). Interestingly, the expression of HES1 protein factor, a Notch target gene^22^, was similar to Notch1 level (Fig. 1A), suggesting that Notch1 signaling is inactive after a long-term in vitro growth during osteoclast differentiation induced by RANKL.

To further explore the effect of active Notch1 signaling on osteoclastogenesis, human CD14^+^CD16^−^ monocytes were transduced with lentivectors encoding the active NOTCH1-ΔE isoform^24^ or empty vector as control and subsequently tracked over time in culture after in vitro RANKL stimulation by flow cytometry. Interestingly, the NOTCH1-ΔE transduced cells did not expand in vitro with respect to control cells (Fig. 1B), suggesting that the constitutive activation of Notch1 signaling might be detrimental for osteoclastogenesis. To address this hypothesis, we also evaluated the abundance of CD14^+^RANK^high^ pre-osteoclasts^25^ by flow cytometry as well as the number of tartrate-resistant acid phosphatase (TRAP) positive cells, which are considered osteoclast-like multinucleated giant cells^26^. We found that the NOTCH1-ΔE transduced cells generated a lower number of CD14^+^RANK^high^ pre-osteoclasts (Fig. 1C,D) and TRAP-positive multinucleated cells (Fig. 1E,F) compared to the control cells, suggesting that the human CD14^+^CD16^−^ monocytes, lentivirally transduced with the active NOTCH1-ΔE isoform were strongly inhibited to generate osteoclasts following in vitro RANKL-stimulation. Similar findings were also obtained from human RANKL-induced PBMCs, after transduction with NOTCH1-ΔE lentiviruses (Fig. S1) and from NOTCH1-ΔE transduced CD14^+^CD16^−^ monocytes maintained in vitro only with RANKL (50 ng/ml) and M-CSF (25 ng/ml) (Fig. S2). Furthermore, the activation of endogenous Notch signaling by human Delta^MAX^ ligand stimulation^27^ also suppressed the RANKL-induced osteoclastogenesis (Fig. S3). Interestingly, we also found that the constitutive activation of Notch1 signaling in CD14^+^CD16^−^ monocytes increased cell proliferation, but enforced cell death during RANKL-induced osteoclastogenesis (Fig. S4). Taken together, these data support the conclusion that the constitutive activation of Notch1 signaling in human CD14^+^CD16^−^ monocytes might block the RANKL-induced osteoclast formation by promoting cell death and differentiation arrest.

### Single-cell transcriptomics reveal Notch1-dependent stages of human osteoclastogenesis

**Figure 2:**
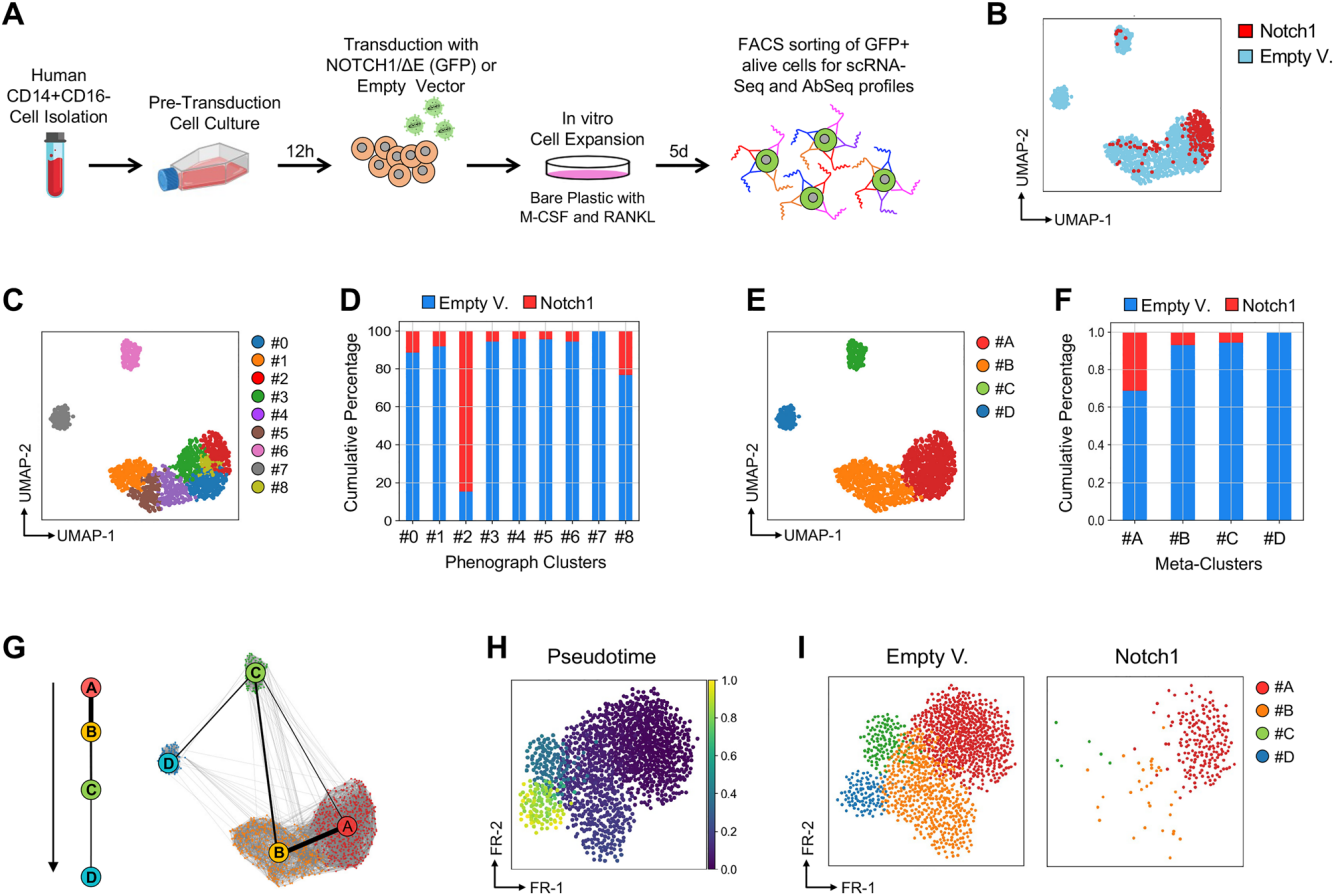
Single-cell analysis reveals differential stages of in vitro human osteoclastogenesis. (**A**) Schematic overview of experimental approach. Specifically, human CD14^+^CD16^−^ monocytes were isolated from PBMCs and transduced with lentivectors encoding the active NOTCH1-ΔE isoform or empty vector as control. Transduced GFP^+^ cells were in vitro stimulated and expanded with RANKL and M-CSF for 5 days and subsequently purified by FACS-sorting for performing single cell RNA-Sequencing (scRNA-Seq) and Ab-sequencing (AbSeq) assays. (**B**) UMAP plots based on the scRNA-Seq and AbSeq data showing the distribution of Notch1-transduced cells in red vs. control cell subsets in light blue. Each dot represents one cell. (**C**) UMAP plot as in Panel B, but with each cluster depicted in a different color as determined by the Leiden graph-clustering method (resolution = 1.4). There was a total of 9 different phenotypic clusters identified by the Leiden algorithm. (**D**) Cumulative percentage of Notch1-transduced and control cells according to each sample group. (**E**) UMAP plot as in Panel B, but with cells colored based on their assigned MetaCluster (MC) as determined by the Leiden graph-clustering method (resolution = 0.4). (**F**) Cumulative percentage of Notch1-transduced and control cells according to each MetaCluster. (**G**) The differentiation and developmental trajectory of human CD14^+^CD16^−^ monocytes following 5 days of in vitro RANKL-stimulation as determined by partition-based graph abstraction (PAGA). Arrow and black bars indicate the direction and the average velocity of the differentiation and progression of each MetaCluster, respectively. (**H**,**I**) Diffusion pseudotime plots depicting each MetaCluster in all cells (**H**) as well as in the Notch1-transduced and control cell subsets (**I**).

To generate an overview of cell lineage specification modulated by Notch1 signaling at the single-cell level at the early stages of human RANKL-induced osteoclastogenesis, we analyzed single-cell transcriptomic data simultaneously with a panel of 31 oligo-conjugated antibodies (AbSeq) against cell surface protein markers (Table S1). Specifically, human CD14^+^CD16^−^ monocytes were isolated from PBMCs and transduced with lentivectors encoding the active NOTCH1-ΔE isoform or empty vector as control. Transduced GFP^+^ cells were in vitro stimulated and expanded with RANKL and M-CSF for 5 days and subsequently purified by FACS-sorting for performing single cell RNA-Sequencing (scRNA-Seq) and Ab-sequencing (AbSeq) assays (Fig. 2A). Initially, UMAP algorithm was used with standard parameters (n_neighbors = 15, min_dist = 0.5, n_components = 2, metric = ‘euclidean’) to perform dimensional reduction on 231 and 1,054 transcriptomes from NOTCH1-ΔE transduced and control cells respectively; this allowed us to visualize cell subsets in two dimensional cartesian spaces. Interestingly, we found that NOTCH1-ΔE transduced cells were differently distributed with respect to control cells (Fig. 2B), suggesting that the constitutive activation of Notch1 signaling in human CD14^+^CD16^−^ monocytes might alter the normal commitment of osteoclast specification. To discriminate between cell subsets with related phenotypes from highly dimensional data, we employed the Leiden clustering algorithm^28^ (resolution = 1.4), which identified a total of 9 distinct population clusters, each including a different fraction of NOTCH1-ΔE transduced and control cells (Fig. 2C,D). In order to identify common phenotypes among any two cell conditions, we also performed a meta-clustering analysis based on the Silhouettes metric^29^ and narrowed the resolution parameter to 0.4, which is the value corresponding to the best Silhouette score, thereby selecting 4 meta-clusters (MCs) from the original 9 (Fig. 2E and Fig. S5). Of note, we found that NOTCH1-ΔE-transduced cells were significantly concentrated on MC-#A group (p-value < 0.001 by Fisher's exact test, p-value < 0.001) and absent in MC-#D subset (Fig. 2F). To explore the role of Notch1 signalling on the cell fate decisions required for development of mature osteoclasts, we traced gene expression change by performing a Partition-based graph abstraction (PAGA)^30^, which quantifies the state of individual cells as well as the connectivity among clusters. Interestingly, the single-cell trajectory and diffusion pseudotime analyses show the progression of different MC groups (Fig. 2G,H), starting from MC-#A cluster and highlighting the complex differentiation processes in which cells make fate decisions to mature osteoclasts. Of note, the MC-#D cluster, only composed of control cells, was enriched in genes of osteoclast differentiation and development by Gene Ontology (GO) enrichment analysis and expressed various markers of the osteoclast lineage, including TYROBP^31^, SNX10^32^ and FOS^33^ (Fig. 2I and Fig. S6), while significant associations with the gene signatures of other cell types were not find in the other clusters, supporting the idea that the activation of Notch1 signaling blocks the differentiation of precursors to mature human osteoclasts.

### Gene expression and immunophenotypic profiles of RANKL-induced stages of osteoclast lineage

**Figure 3:**
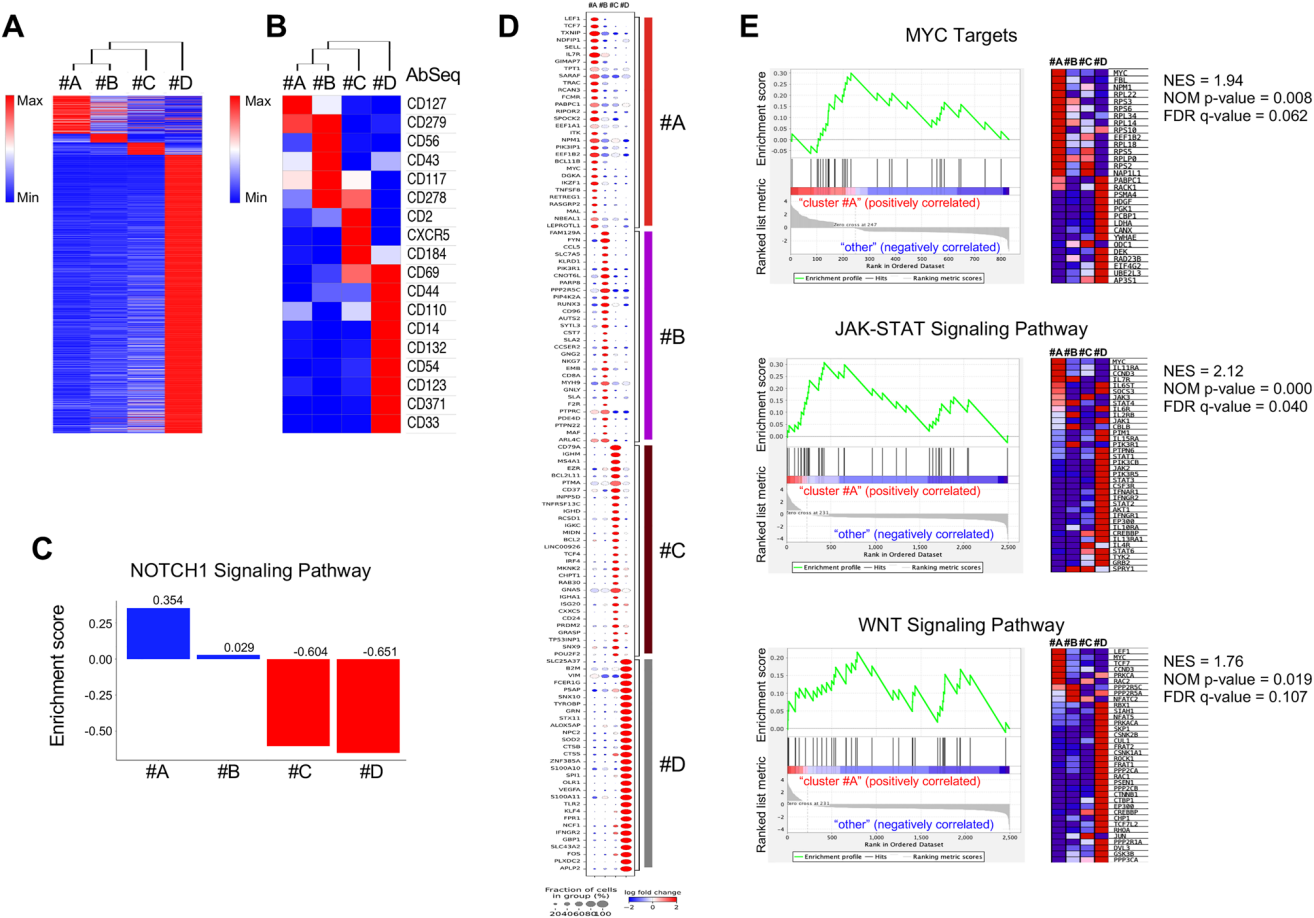
Gene expression and immunophenotypic profiles displays known and previously uncharacterized markers of human osteoclastogenesis. (**A**) Heatmap and hierarchical clustering of gene expression scRNA-Seq data for each MetaCluster (MC) as identified by the Leiden clustering algorithm and generated by Seaborn software (Version: 0.12.2). Gene list is reported in Table S8. (**B**) Expression heatmap and hierarchical clustering of all surface markers recognized by 31 oligo-conjugated antibodies (Abseq panel in Table S1) in each of the four MetaClusters as generated by Seaborn software (Version: 0.12.2). (**C**) The enrichment score of cells of indicated MetaClusters across the genes related to KEGG NOTCH1 signaling pathway (hsa04330) by gene set variation analysis (GSVA). The bar above zero (blue color) represent subsets with gene enrichment in the NOTCH1 signaling pathway using a non-parametric and unsupervised approach. (**D**) Dot plot of highly differentially expressed genes (adjusted p-value < 0.05) in cells of indicated MetaClusters displays known and previously uncharacterized markers of human osteoclastogenesis as generated by Scanpy toolkit (Version: 1.8.1) at the following link https://scanpy.readthedocs.io/en/stable/generated/scanpy.pl.rank_genes_groups_dotplot.html. (**E**) Gene set enrichment analysis (GSEA) of indicated gene sets and signaling pathways as enriched in MC-#A. To the left, the enrichment score (ES) plots show the profile of running ES and positions of gene set members on the rank-ordered list for the Myc target genes from HALLMARK database as well as the genes of JAK/STAT and WNT signaling pathways from the KEGG database at the following link https://www.gsea-msigdb.org/. Heatmaps of the top marker genes for each phenotype are reported on the right. NES, normalized enrichment score; NOM, nominal; FDR, false discovery rate.

Human osteoclasts can be generated in vitro from peripheral blood upon stimulation induced by RANKL and M-CSF factors^5–7^. Nonetheless, the cell stages and progression of human RANKL-induced osteoclastogenesis were never described before. To highlight the major changes in the gene expression and cellular pathways of different osteoclast precursors, we determined the transcriptional and immunophenotypic profiles of main cell subsets, detected by the scRNA-Seq and AbSeq assays. Initially, we validated and immunophenotypically characterized the four MC groups based on differential gene expression analyses (Fig. 3A) as well as on their immunophenotypic profiles as determined by the panel of 31 oligo-conjugated antibodies in the AbSeq assay (Fig. 3B). Interestingly, distinct cell surface markers were significantly highly expressed in each of four MC subsets. Specifically, we found that CD127 was enriched in MC-#A; CD279, CD56, CD43 and CD117 in MC-#B; CD2, CXCR5 and CD184 in MC-#C and CD44, CD110, CD14, CD132, CD54, CD123, CD371 and CD33 in MC-#D (Figs. S7, S8). We also validated the highlighted immunophenotypic changes and the progression of the RANKL-induced stages of osteoclast lineages by multiparameter flow cytometry (Fig. S9). Notably, we. found an increase of M1 macrophages in the Notch1-transduced cells with respect the control cell subsets, suggesting that the activation of Notch1 signaling might promote the development of pro-inflammatory M1 Macrophages to inhibit RANKL-induced osteoclastogenesis as previously reported^34^.

To further detail the divergence between different subsets of osteoclast precursors, we determined the gene expression profiles and cellular pathways of highlighted four MetaClusters. Hence, we performed a gene set variation analysis (GSVA) to assess variations of the Notch1 pathway activity over the different subsets of control cells in an unsupervised manner. Consistently with our previous results, we observed that MC-#A and MC-#B cell populations were enriched in genes of Notch1 signaling pathway (Fig. 3C). Moreover, we found that the Notch ligand, Jagged1 (JAG1) was highly expressed in the most mature cells enriched in MC-#D (Fig. S11), suggesting that there might be a reciprocal Notch1 ligand supply between cells in the considered experimental system. Distinct gene signatures were also observed in all cell states of osteoclastogenesis (Fig. 3D), including TXNIP and IL7R at the early stages in MC-#A, which are known modulators of osteoclast differentiation^35–39^ as well as distinct metabolic gene signatures and gene markers (Fig. S12). Interestingly, gene set enrichment analysis (GSEA) identified an enrichment of target genes of c-MYC transcription factor, which is directly modulated by Notch1^40,41^ as well as JAK/STAT and WNT signaling pathways in MC-#A (Fig. 3E and Tables S2–S5), highlighting potential functional mechanisms of osteoclast differentiation at the early stages.

### Cell–cell communications and molecular interactions in RANKL-induced osteoclastogenesis

**Figure 4:**
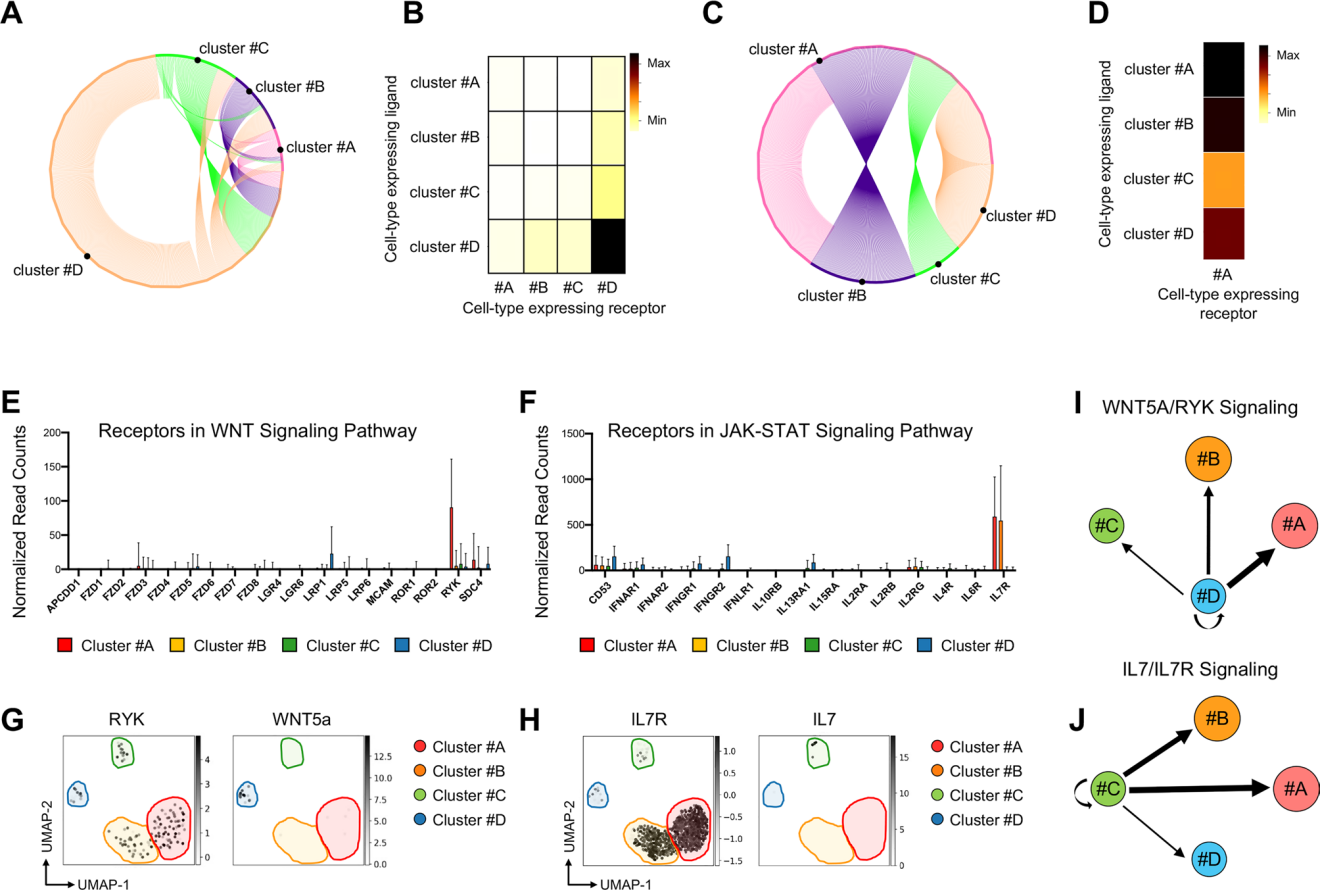
Cell–cell communication network in RANKL-induced osteoclastogenesis. (**A**) Circos-view of interaction network constructed by NATMI1 algorithm. Edges are colored by ligand expressing cell type, lanes connect target cell type and thickness is relative to the number of ligand–receptor pairs connecting the two clusters. (**B**) Heatmap of interaction network as reported in A using the NATMI1 method (https://hub.docker.com/r/asrhou/natmi). Cell subsets expressing the receptors are listed in rows. Columns indicate cells expressing the receptors. Color is relative to the count number of ligand–receptor pairs. (**C**,**D**) Circos-view and heatmap of interaction network of MC-#A cells using the NATMI1 method (https://hub.docker.com/r/asrhou/natmi). (**E**,**F**) mRNA expression level of human genes encoding the cell receptors involved in WNT (**E**) and JACK-STAT (**F**) signaling pathways as reported in the KEGG database at the following link https://www.gsea-msigdb.org/. In the plots, read counts, normalized by median ratio of total counts over all genes are reported for the indicated MetaClusters (MC) by SCANPY toolkit (Version: 1.8.1) at the following link https://scanpy.readthedocs.io/en/stable/generated/scanpy.pl.rank_genes_groups_dotplot.html. (**G**,**H)** UMAP plots based on the single cell RNA-Sequencing (scRNA-Seq) data showing the expression of RYK receptor and WNT5a ligand (**G**) as well as the level of IL7R receptor and IL7 ligand (**H**) in the highlighted MetaClusters (MC) of osteoclast differentiation. (**I**,**J**) Visualization of cell types connected based only on WNT5a/RYK (**I**) or IL7/IL7R (**L**) matrix (ligand-receptors) by NATMI algorithm. The thickness of lanes is relative to the number of ligand–receptor pairs connecting the two clusters.

Our previous results suggest that extrinsic modulators may promote cell-type-specific diversity. Therefore, to identify and characterize ligand-receptor relationships in our system, we applied the NATMI algorithm that infers a cell-to-cell communication network by calculating the number of ligand-receptor pairs among all clusters of control cells^42^ (Fig. S13). Interestingly, we found an increased expression of both ligands and receptors in the cells derived from MC-#D subset (Fig. 4A,B), suggesting a higher autocrine and paracrine signaling activities of these cells compared to the other clusters. Furthermore, to explore cell–cell interactions active during the early stages of osteoclast differentiation, we computationally isolated cells derived from MC-#A population and applied NATMI algorithm to identify signaling crosstalk among the different clusters. We found an increased number of ligand-receptor pairs among MC-#A and MC-#B as well as MC-#A and MC-#D (Fig. 4C,D). As expected, we also highlight a pronounced WNT and JAK/STAT signaling mostly involving MC-#A and MC-#B subsets. Specifically, we found that RYK and IL7R were the most expressed receptors in WNT and JAK/STAT pathways, respectively (Fig. 4E,F). Interestingly, we found that WNT5a, a noncanonical WNT ligand was associated to RYK receptor and mainly expressed by MC-#D cell subset (Fig. 4G). Interleukin-7 (IL-7) was instead mainly expressed by cells derived from MC-#C cluster (Fig. 4H). Taken together, these data suggest a model of cell extrinsic regulations of osteoclast progenitors, which induce WNT and JAK/STAT signaling pathways and are modulated by WNT5a and IL7 secreted factors, respectively (Fig. 4I,J).

### Notch1 and IL7R signaling axis primes the expansion of osteoclast progenitors

**Figure 5:**
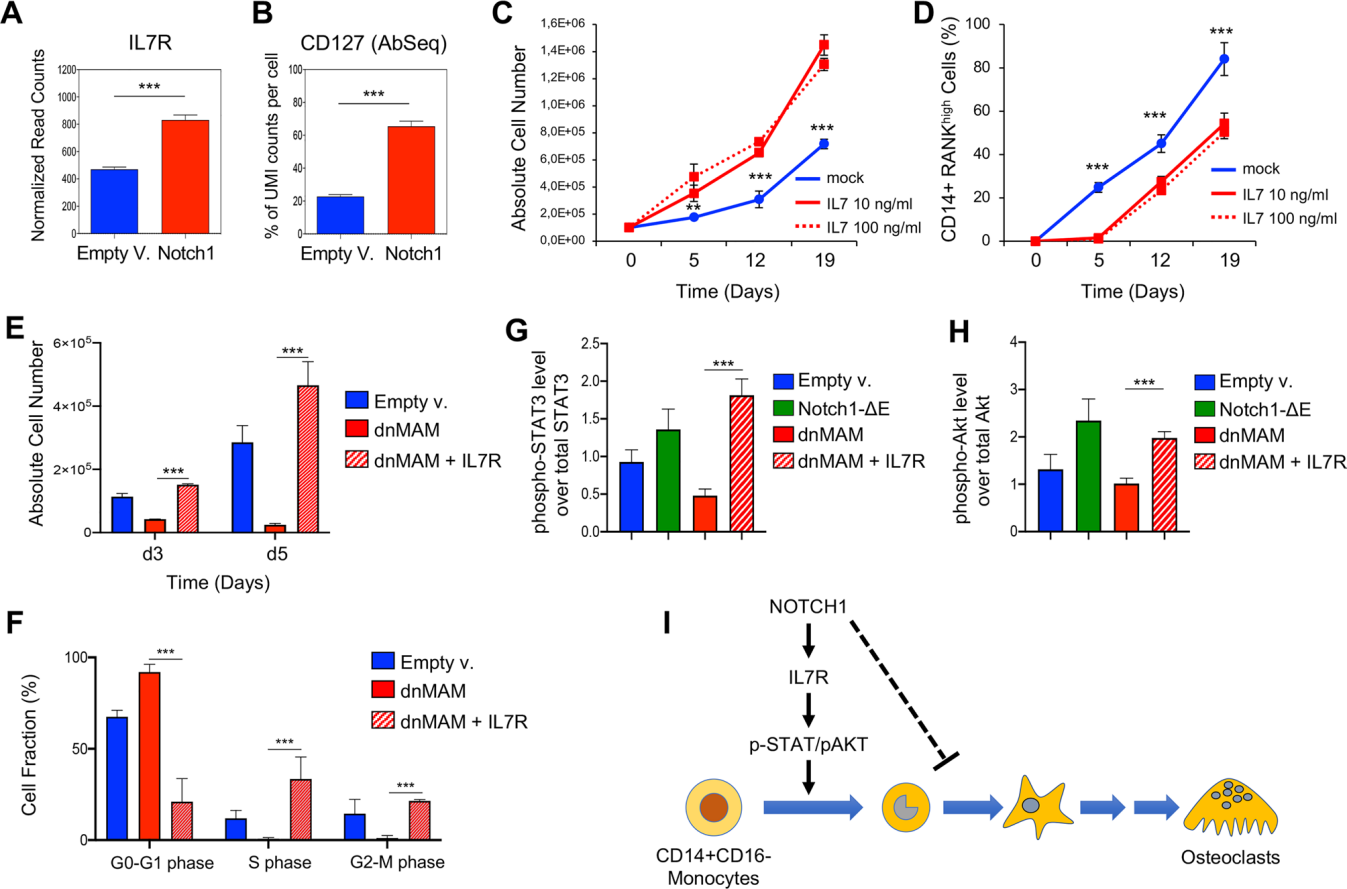
Constitutive activation of IL7R signaling rescues the cell phenotypes induced by the block of Notch1 signaling pathway in osteoclast progenitors. (**A**) mRNA expression level of IL7R gene in Notch1-transduced vs control bulk cells as determined by scRNA-seq data. ***p < 0.001 (Student’s *t*-test). (**B**) Percentage of UMI counts of CD127 oligo-conjugated antibody in Notch1-transduced vs control bulk cells as determined by Ab-seq assay. ***p < 0.001 (Student’s *t*-test). (**C**,**D**) Flow cytometric analysis of abundance of total cells (**C**) and CD14^+^RANK^high^cell fraction (**D**) in human CD14^+^CD16^−^ monocytes, isolated from peripheral blood and in vitro RANKL stimulation in presence of recombinant IL7 at the indicated concentration (10 ng/ml and 100 ng/ml) or phosphate-buffered saline (PBS) as mock control. Abundance of total cells (**C**) and CD14^+^RANK^high^ cell fraction (**D**) was tracked over time at the indicated time points by flow cytometry. Alive cells were discriminated using the LIVE/DEAD™ Fixable Near-IR stain. Means ± SD fraction of the initial transduction value are plotted for experiments performed in biological triplicates. **p < 0.01; ***p < 0.001 (Two-way ANOVA with Dunnett’s test, comparing the mock control mean with the other values). (**E**) Flow cytometric analysis of abundance of total cells after transduction with dnMAM alone (dnMAM) or in combination with IL7R_P2mut (dnMAM + IL7R) construct, harboring the p.Thr244_Ile245insCysProThr mutation to induce constitutive signaling, or empty vector as control. Cell subsets were measured after 3 and 5 days of in vitro RANKL stimulation by flow cytometry. The graphs report the result of two independent experiments performed in biological triplicates. ***p < 0.001 (Student’s *t*-test). (**F**) Flow cytometric analysis of cell proliferation by BrdU incorporation in human CD14^+^CD16^−^ monocytes, following transduction with dnMAM alone (dnMAM) or in combination with IL7R_P2mut (dnMAM + IL7R) construct or empty vector as indicated. Cells were measured after 3 days of in vitro RANKL stimulation by flow cytometry. The graphs report the result of two independent experiments performed in biological triplicates. ***p < 0.001 (Student’s *t*-test). (**G**,**H**) Expression level of total and phosphorylated proteins of STAT3 (**G**) and Akt (**H**) factors in human CD14^+^CD16^−^ monocytes, following transduction with NOTCH1-ΔE, dnMAM alone (dnMAM) or in combination with IL7R_P2mut (dnMAM + IL7R) construct or empty vector as indicated. Cells were measured after 3 days of in vitro RANKL stimulation by flow cytometry. Each graph reports the ratio of mean fluorescence intensity (MFI) of phosphorylated protein over the MFI of total protein in two independent biological experiments. **p < 0.01; ***p < 0.001 (Student’s *t*-test). (**I**) Schematic of the signaling pathways involving NOTCH1 and IL7R in the expansion of osteoclast progenitors at the early stage of human RANKL-induced osteoclastogenesis.

To highlight functional correlations between Notch1 signaling and cell extrinsic regulators of osteoclast progenitors, we focused on the IL7/IL7R pathway. It was indeed reported that IL7R gene expression is directly modulated by NOTCH1 in normal T cells^43^. Moreover, the intracellular domain of NOTCH1 receptor binds the promoter region of the human IL7R locus in CUTLL1 cell line, which is derived from a human T-cell lymphoma^44,45^ (Fig. S14). To explore the NOTCH1/IL7R signaling axis during human RANKL-induced osteoclastogenesis, we compared the expression level of IL7R transcript and CD127 protein among Notch1-transduced and control cells using the scRNA-seq and Ab-seq data. As expected, we found a statistically significant increase of both transcript and protein expression in NOTCH1-ΔE transduced cells with respect to control cells (Fig. 5A,B). We also detected a significant reduction of IL7R protein in cells, lentivirally transduced with a dominant-negative form of Mastermind-like protein 1 (dnMAM), which shutdowns Notch signaling^46^ (Fig. S15), supporting the idea that NOTCH1 is a direct modulator of IL7R expression in osteoclast progenitors.

Signaling mediated by IL7R has a fundamental role in the development and homeostasis of different cell types, including T cells, monocytes, endothelial cells and fibroblasts^37,47^. To explore the effects of IL7/IL7R signaling on the RANKL-mediated osteoclast differentiation, human CD14^+^CD16^−^ monocytes were isolated from peripheral blood and subsequently cultured upon RANKL stimulation in the presence of recombinant human IL7 at two different concentrations up to 19 days (Fig. 5C). Notably, addition of recombinant IL7 to cell cultures induced higher expansion with respect to mock treated cells (Fig. 5C). Nonetheless, the IL7-treated cell subsets generated a lower number of CD14^+^RANK^high^ pre-osteoclasts (Fig. 5D), supporting the idea that IL7R signaling pathway enforces the expansion of osteoclast progenitors at the early stages following in vitro RANKL-induction. To assess the hypothesis that Notch signaling might induce IL7R signaling to enforce the expansion of osteoclast progenitors, human CD14^+^CD16^−^ monocytes were transduced with the active NOTCH1-ΔE isoform and in vitro RANKL-stimulated in presence of neutralizing antibody against IL-7R or mouse IgG1 isotype as mock control. Interestingly, the inhibition of RANKL-induced osteoclastogenesis due to constitutive activation of Notch1 signaling in human osteoclast precursors was abolished in vitro upon the addition of a neutralizing antibody against IL-7R (Fig. S16). Human CD14^+^CD16^−^ monocytes were also transduced with dnMAM alone (dnMAM) or in combination with IL7R_P2mut (dnMAM + IL7R) construct, harboring the p.Thr244_Ile245insCysProThr mutation, which promotes constitutive IL7R signaling^48^. Of note, the activation of IL7R pathway promoted cell expansion and rescued the apoptotic phenotype induced by the block of Notch signaling pathway in dnMAM-transduced cells (Fig. 5E and Fig. S17). Furthermore, the constitutive activation of IL7R in dnMAM cells significantly increased the proliferation rate with respect to other cell conditions (Fig. 5F), suggesting that Notch1 and IL7R signaling axis enforces the proliferation of osteoclast progenitors at the early stage of human RANKL-induced osteoclastogenesis. Interestingly, we also found that the NOTCH1 target gene, c-MYC modulated the expression of IL7R receptor (Fig. S18A,B). Moreover, the shRNA-mediated knockdown of c-MYC gene in NOTCH1-ΔE-transduced cells enforced the cell-death (Fig. S18C), highlighting its role as crucial mediator of Notch1 signaling pathway in osteoclast progenitors.

The binding of IL7 ligand to IL7R activates two main pathways including the Janus kinase (JAK)/signal transducers and activator of transcription (STAT) and phosphoinositide-3 kinase (PI3K)/Akt, leading cell survival and development^36,37,47,49–51^. Notably our scRNA-seq data suggest that JAK/STAT signaling pathway is enriched at the early stage of RANKL-induced osteoclastogenesis (Fig. 3E). To validate this hypothesis and to determine how IL7R promote cell expansion of osteoclast progenitors, we assessed components of early IL7R signaling, including p-STAT3 and p-Akt in dnMAM-transduced cells by flow cytometry. We discovered that activation of both STAT3 and Akt factors were induced by IL7R without an active Notch1 signaling pathway (Fig. 5G,H), suggesting that IL7R promotes the expansion of osteoclast progenitors at the early stages through STAT3 and Akt signaling mediators (Fig. 5I).

## Discussion

Osteoclasts are large multinucleated cells, differentiated from monocyte precursors and responsible for the absorption and dissolution^1^. Previous works have reported that the Notch signaling pathway affects mouse osteoclast precursors^14–16^. Moreover, more recent studies have highlighted novel trajectories of osteoclasts differentiation using single-cell RNA-Seq analysis^52,53^. Nonetheless, despite these results the effect of Notch1 signaling on human osteoclast differentiation is still poorly characterized. In this study, we explored the functional role underlying the Notch1 activity in human osteoclastogenesis. Specifically, we reported that the constitutive activation of Notch1 signaling pathway promotes a cell differentiation arrest of human mononuclear CD14^+^ cells upon in vitro RANKL-induced stimulation to osteoclasts. Through a scRNA-Seq profiling of NOTCH1-ΔE transduced vs. control cells, we further demonstrated that Notch1 signaling is mainly required at specific stages of human osteoclastogenesis. Interestingly, we revealed four different stages of cell differentiation upon in vitro RANKL-induced stimulation by unsupervised learning methods. These cell subsets were characterized by distinct gene expression and immunophenotypic profiles and active Notch1, JAK/STAT and WNT signaling pathways. Recent studies based on scRNA-Seq technology have also highlighted different cell subsets of osteoclast precursors during human and murine osteoclastogenesis, including CD11c-expressing dendritic cell (DC)-like populations at the early stage^52,54–56^. In agreement with these observations, we also identified distinct cell subsets of human osteoclast progenitors. Furthermore, we found that the Notch signaling pathway is a relevant modulator of cell expansion of osteoclast precursors as well as of osteoclast cell fate commitment. Additionally, cell–cell communication analyses revealed extrinsic modulators of osteoclast progenitors including the IL7/IL7R and WNT5a/RYK axes. Moreover, we found that IL7R gene expression is directly modulated by NOTCH1 in the RANKL-induced cells and that, through Akt and STAT3 signaling mediators, Notch1 and IL7R signaling pathways enforce the expansion of osteoclast progenitors following in vitro RANKL-induction.

Taken together, these findings suggest a model in which an active Notch1 signaling blocks the late stages of human osteoclast formation. Interestingly, it was reported that the constitutive activation of Notch1 negatively regulates mouse osteoclast development and formation by inhibiting the expression of macrophage colony-stimulating factor in mesenchymal cells inhibits^15^. Additionally, the binding of Jagged1 ligand to Notch1 receptor suppresses osteoclastogenesis contrarily to Notch2/Delta-like 1 axis^18,23^. This suggests that the Notch receptors might differently modulate osteoclast development by responding to unique stimuli or extrinsic modulators that promote cell-type diversity. Moreover, these findings propose that the effect of Notch signaling pathway is directly dependent on the cell stage of osteoclast progenitors and mainly involved in the progress of human osteoclastogenesis at the early phases.

Furthermore, while the relevance of IL7R/IL7 signaling was already explored in osteoclast activity and differentiation^35,36^, the identified link between IL7R and Notch1 pathways is likely to be the most relevant in the context of PI3K/AKT and STAT mediators. Interestingly, recent studies indicate that JAK/STAT inhibitors, such as tofacitinib, significantly moderate the symptoms of rheumatoid arthritis and reduce the progression of osteoclasts as well as structural joint damage^57^. Moreover, it was reported that IL-7 contributes to inflammation and tissue destruction in patients with rheumatoid arthritis in a unique manner^37^ and the blockade of IL-7/IL-7R signaling pathway protected collagen-induced arthritis (CIA) mice from bone degradation^51^. Taken together, these results support the therapeutic role of Notch1/IL7R axis in osteoclast-related diseases. Nonetheless, further studies will be required to explore the intriguing possibility that therapies which modulate Notch and/or IL7R expression or signaling activity, may modulate osteoclast progression and thereby improve clinical outcomes in patients with rheumatoid arthritis or other osteoclast-related diseases.

### Supplementary Information

Supplementary Information.

## Data Availability

The scRNA-Seq datasets generated during the current study are available in the NCBI Sequence Read Archive (SRA) repository (cod. PRJNA914118) (reviewer link: https://dataview.ncbi.nlm.nih.gov/object/PRJNA914118?reviewer=gipq08ecnitd79an81gmk3sees). For other original data, please contact the corresponding author.
