# Supplementary material for: The Notch1 signaling pathway directly modulates the human RANKL-induced osteoclastogenesis
Source: Sci Rep. 2023 Dec 1;13:21199. doi: 10.1038/s41598-023-48615-2 (PMC10692129; doi:10.1038/s41598-023-48615-2)
Supplement: Supplementary file 1 — Supplementary Information. [file 41598_2023_48615_MOESM1_ESM.pdf]

The Notch1 signaling pathway directly modulates the human RANKL-Induced osteoclastogenesis

Costanzo Padovano[1,*], Salvatore Daniele Bianco[2,*], Francesca Sansico[1], Elisabetta De Santis[1], Francesco Tamiro[1], Mattia Colucci[1], Beatrice Totti[1], Serena Di Iasio[1], Gaja Bruno[1], Patrizio Panelli[1], Giuseppe Miscio[3], Tommaso Mazza[2,*], Vincenzo Giambra[1,*,±]

[1]Institute for Stem Cell Biology, Regenerative Medicine and Innovative Therapies (ISBReMIT), Fondazione IRCCS "Casa Sollievo della Sofferenza", 71013 San Giovanni Rotondo (FG), Italy

[2]Bioinformatics Unit, Fondazione IRCCS Casa Sollievo della Sofferenza, 71013 San Giovanni Rotondo, Italy;

[3]Clinical Laboratory Analysis and Transfusional Medicine, Fondazione IRCCS "Casa Sollievo della Sofferenza", 71013 San Giovanni Rotondo (FG), Italy

Supplemental Information including:

Supplementary Methods
Supplementary References
Figures S1 to S19
Tables S1 to S8

Materials and Methods

Viral transduction. Lentiviral particles were produced by transient co-transfection of 293T cells with packaging/envelope vectors and concentrated by ultracentrifugation. Viral transduction was performed by spinfection in the presence of polybrene as reported[1]. Virally transduced cells were FACS-sorted as applicable. The lentivector encoding the human active NOTCH1-ΔE isoform[1], dominant negative version of MAML1 (DNMAM)[2] and IL7R_P2mut with the p.Thr244_Ile245insCysProThr mutation[3] were based on pRRLsin.cPPT.MNDU3.PGK.GFP.WPRE backbone and kindly provided by Dr. Andrew P. Weng (BCCRC, Vancouver, Canada). The RNAi Consortium (TRC) shRNAs targeting C-MYC (shMYC_#40, TRCN0000039640; shMYC_#42, TRCN0000039642) and NOTCH1 (shNOTCH1_#30, TRCN0000350330; shNOTCH1_#61, TRCN0000003361) were cloned into a derivative of pLKO.1 vector (Addgene #8453) with the mTag2BFP selection marker. The lentivector encoding the Delta^{MAX} Notch ligand[4], conjugated with a rabbit IgG1 (Fc specific, Heavy Chain) was based on pRRLsin.cPPT.MNDU3.PGK.GFP.WPRE backbone and kindly provided by Dr. Vincent C. Luca (Moffitt Cancer Center, Tampa, USA).

RNA extraction and real-time PCR

Total RNA was extracted using RNeasy Mini Kit (Qiagen, Hilden, Germany), treated with Dnase-Rnase free (Qiagen, Hilden, Germany) and quantified by Nanodrop (Thermo Fisher Scientific). First strand cDNA was generated from total RNA by reverse transcription with SuperScript III/VILO master mix (Invitrogen) including a combination of random 15-mer and anchored oligo(dT) primers. The following TaqMan probe-based assays were used: HES1 (Hs00172878_m1; FAM), HEY1 (Hs00232618_m1; FAM), DTX1 (Hs01092201_m1; FAM), c-MYC (Hs00153408_m1; FAM), CALCR (dHsaCPE5057778; FAM), MMP9 (dHsaCPE5050120;

FAM), CTSK (dHsaCPE5055058; FAM), and B2M (Hs99999907_m1; VIC, primer limited) (Applied Biosystems/ThermoFisher). qRT-PCR experiments were performed using a QuantStudio 12K Flex Real-Time PCR System (ThermoFisher Scientific) with manufacturer's recommended cycling conditions (Qiagen).

Protein extraction and Western Blot assay. Total cells were collected, washed in ice-cold phosphate-buffered saline and subsequently lysed in ice-cold 50mM Tris-HCl (pH 7.4), 0.25% sodium deoxycholate, 1% Nonidet P-40, 150mM sodium chloride, 1mM sodium orthovanadate, 1mM sodium fluoride, 2.5mM sodium pyrophosphate, 1mM EDTA, 1mM phenylmethylsulphonyl fluoride, and protease inhibitor cocktail (cat #539134, Calbiochem). Whole cell lysates were quantified using the Pierce BCA protein assay kit (cat. 23227, ThermoFisher) and 25µg of total proteins were incubated at 95°C for 10 minutes, loaded on SDS-PAGE gels and then transferred to Hybond-ECL membranes (Amersham). The membranes were blocked with 5% milk/0.3% TBS-Tween20 at 4°C for 1 hour and then probed with primary antibodies against Notch1 (C-20) (1:1,000 dilution; cat. sc-6014, Santa Cruz Biotechnology), cleaved Notch1 (Val 1744) (1:1,000 dilution; cat. 4147, Cell Signaling) or HES1 (1:1,000 dilution; cat. ab71559, AbCam) and β-Actin (1:6,000 dilution; cat. A1978, Sigma). HRP-conjugated secondary antibodies (Cat. NEF812001EA, Perkin Elmer) were used at 1:10000 dilution. The chemiluminescent signal was detected with enhanced chemiluminescence (ECL) (cat.32106, ThermoFisher) and subsequently autoradiography. The blots were cut prior to hybridisation with antibodies and ll images were generated by the ChemiDoc MP Imaging System.

Generation of Delta^{MAX} -beads. To load Fc-Delta^{MAX} proteins onto the surface of magnetic beads, Delta^{MAX} 293T cell overexpression lysate was incubated with Dynabeads Protein G (cat. #10003D, ThermoFisher). Briefly, the cultured cells were first harvested, washed 3 times in 5ml of ice-cold PBS, and then lysed in RIPA buffer (without SDS) supplemented with protease and phosphatase inhibitors. The lysed cells were centrifuged at 16,000 rpm for 10 min at 4°C. Next, 10ul of beads were pipetted into 1.5 ml microcentrifuge tubes, placed in a magnetic stand (Dynal Bead Separator Rack, Invitrogen), washed in PBS, and then pre-blocked with PBS/BSA 5% and Glycine 100mM (blocking buffer) for 1h at room temperature. After magnetic separation, beads were incubated in 40ul of cell lysate overnight at 4°C with constant mixing. The next day, beads were again washed three times in 100ul of PBS/Tween 0.1% to remove nonspecifically bound proteins, eluted in 100ul of PBS (pH 7.0) and immediately stored at 4°C. No transduced HEK-293T cell lysate was used as a negative control. Secondary Antibodies anti-IgG (anti-mouse/AlexaFluor488 or anti-Rabbit/AlexaFluor647, Invitrogen,1:500) were used for detection of background noise and Delta^{MAX} proteins loading by flow cytometry analysis (BD LSRFII Fortessa instrument).

Flow cytometry assays. Human cells were stained with fluorochrome antibodies against CD45 (monoclonal antibody (HI30), eFluor™ 506, eBioscience), CD14 (monoclonal antibody (MφP9), PE, BD Biosciences), CD16 (monoclonal antibody (eBioCB16 (CB16)), APC, eBioscience), CD127 (monoclonal antibody (HIL-7R-M21), BV605, BD Biosciences) and human RANK/TNFRSF11A (monoclonal antibody (Clone 80704), R&D Systems). An anti-mouse IgG Alexa Fluor568- or Alexa Fluor647conjugated secondary antibodies (cat. A-11004 and A-21235, ThermoFisher) were used to detect the unconjugated antibody against RANK. The LIVE/DEAD™ Fixable Near-IR dead cell stain kit (cat. L34975, ThermoFisher) or DRAQ7™

(1:200 dilution; cat. #564904, BD Biosciences) were also included for identifying the fraction of total living cells as indicated. Panel of cell surface markers and fluorophore-conjugated antibodies used in the multiparameter flow cytometry assessment of human CD14+CD16- monocytes, transduced with NOTCH1-ΔE lentiviruses or empty vector as control is reported in Table S6. We performed intracellular staining with an anti-cMyc Alexa Fluor 647-conjugated antibody (1:50 dilution; cat # MA1-980-A647, ThermoFisher), an anti-phospho-Akt (Ser473) PE-Cy7-conjugated antibody (1:50 dilution; cat #88106, Cell Signaling), an anti-Akt (pan) Alexa Fluor 647-conjugated antibody (1:50 dilution; cat # 5186, Cell Signaling), an anti-phospho-STAT3 (Tyr705) APC-conjugated antibody (1:50 dilution; cat #17-9033-42, ThermoFisher) and an anti-STAT3 (pan) PE-conjugated antibody (1:50 dilution; cat #MA5-23569, ThermoFisher) after paraformaldehyde fixation and permeabilization with 90% ice-cold methanol as specified by the manufacturer. We measured cell proliferation by BrdU incorporation according to the manufacturer's instructions (BrdU kit, BD Biosciences). Absolute cell counts were determined using AccuCheck Counting Beads (cat #PCB100, ThermoFisher) following the manufacturer's instructions. Early apoptotic cells were determined by AnnexinV binding and 7AAD exclusion using the PE Annexin V Apoptosis Detection Kit (Cat. No. 559763, BD PharmigenTM) and following the manufacturer's recommendations. Cell cycle analysis was performed by BrdU incorporation according to the manufacturer's instructions (Cat. No. 557892, BD Biosciences). The MACSPlex Cytokine 12 Kit (cat. 130-099-169, Miltenyi Biotec.) was employed for determining the concentrations of following cytokines: GM-CSF, IFN-α, IFN-γ, IL-2, IL-4, IL-5, IL-6, IL-9, IL-10, IL-12, IL-17 and TNF-α in the conditioned media derived from human CD14+CD16- monocytes, transduced with NOTCH1-ΔE lentiviruses or empty vector as control after RANKL-stimulation at days 5, 10 and 15. Specifically, 1ml of conditioned media from each

cell condition was incubated overnight in the dark on a shaking platform (1400rpm) at room temperature with 20 µL of MACSPlex Capture Beads and treated according to the manufacturer's recommendations. After staining, the beads were coated with capture antibodies against the reported soluble analytes and detected by flow cytometry. We performed FACS analysis and sorting on FACS Canto2 and MoFlo Astrios cell sorter (Beckman Coulter). Flow cytometry data were analyzed by FlowJo software (Becton Dickinson). GraphPad-Prism 8.4.3 software was employed for the visualization and statistical data analyses.

Library generation for Single cell RNA-sequencing (scRNA-Seq) and Ab-sequencing (AbSeq). The cell viability and concentration were determined with the BD Rhapsody Scanner system after staining with viability dyes, Calcein AM (1:200 dilution; cat. #C1430, ThermoFisher) and DRAQ7™ (1:200 dilution; cat. #564904, BD Biosciences), and incubation for 5 min at 37°C. Cells were counted using the Improved Neubauer Hemocytometer (INCYTO). Afterward, GFP+ cells for each sample condition, derived from two biologically independent replicates, were pooled equally in 650ml cold BD Sample Buffer and a BD Rhapsody cartridge was loaded with 10,000 pooled cells for single cell separation. Single cells were isolated using Single-Cell Capture and cDNA Synthesis with the BD Rhapsody Express Single-Cell Analysis System according to the manufacturer's recommendations (BD Biosciences). Based on the number of viable cells revealed and captured on the beads, the final resuspension volume was calculated to subsample and sequence about 2,000 cells. Whole transcriptome, Sample Tag, and BD™ AbSeq amplification were performed with the BD Rhapsody Whole Transcriptome and AbSeq Amplification Kit (cat. #633774), following the manufacturer's instructions. Unwanted PCR products and other small molecules were excluded performing a side cleanup using the AMPure XP Beckman magnetic

beads (cat. #A63880, Beckman Coulter). DNA quantity and quality control were performed using the Qubit™ dsDNA HS Assay Kit (cat. # Q32851, ThermoFisher Scientific) and the electrophoresis system Agilent 2200 TapeStation, cartridge (cat. #5067-5584). Sequencing was performed in paired-end mode (2*75 cycles) on NextSeq 500 System (Illumina) with the NextSeq 500/550 High Output Kit v2.5 (150 Cycles) chemistry to reach a depth of 75,000 reads for WTA, 21,500 reads for AbSeq and 500 reads for SMK per cell for a total of 97,000 reads per cell on average.

scRNA-Seq data analysis. Sequencing data were processed on the Seven Bridges Platform (https://www.sevenbridges.com/) for sample demultiplexing and generation of expression sparse matrices. UMI counting was done including sequencing reads correction, recursive substation error correction (RSEC) and distribution-based error correction (DBEC). Validated reads were aligned to the reference human genome GRCh38, quantified and subsequently, their counts were reported per-genes in a matrix. Highly dimensional scRNA-Seq data were analyzed using SCANPY ver. 1.8.1 for visualization, clustering by uniform manifold approximation and projection (UMAP) algorithm, and differential expression analysis[5-7]. The identified cells in all biological replicates were aggregated. SCANPY was used to make a first data check and trim out outlier cells, genes and mitochondrial transcripts in order to increase the dataset quality[8]. Cells expressing outlier total counts of genes were dropped, and in general all cells exhibiting more than 3000 piled-up reads. Read counts of the remaining 1336 cells were normalized on the median value of all the cell counts, *log1p*-transformed, and scaled. Differential gene expression analyses, gene set variation analysis (GSVA)[9], Gene Set Enrichment Analysis (GSEA)[10] and Gene Ontology (GO) enrichment analysis[11] were performed on the preprocessed read counts using R ver. 4.2.2. Mann-Witney U

test was performed to find differentially expressed genes and p-values were corrected using the Benjamini-Hochberg method.

Cell clustering analysis. Cell-clustering and sub-clustering analyses were performed using the Leiden graph-clustering method[12] using two distinct resolution values that were obtained through the Silhouette metrics. We also used the Partition-based graph abstraction (PAGA)[13] method to generate graph-like maps of cells that preserve both continuous and disconnected structure in data at multiple resolutions and, in particular, to reconstruct the lineage relationships and to trace the gene expression changes among clusters. We then used the UMAP algorithm for dimension reduction and to visualize these results.

Pseudotime trajectory analysis. Trajectory inference was conducted using the Diffusion Pseudotime (DP)[14] function of SCANPY with the aim to analyze the evolutionary processes of cells. DP was first applied to the root cells in the "A" cluster to detect any branching trajectory. Data was *log1p*-normalized and scaled after the application of the DP algorithm.

Cell–cell communication analysis. Cell-cell communication analysis, based on the interaction between ligands and receptors, was performed using the Network Analysis Toolkit for the Multicellular Interactions (NATMI) Python package (https://github.com/asrhou/NATMI). To perform the NATMI algorithm, the gene expression matrix was normalized following the author's recommendations [15]. To evaluate and visualize the interactions, the detection threshold parameter was set to 20% (commonly applied to single-cell datasets) and, then, lowered to 0, to remove any

arbitrary threshold on specificity of the interactions. All other parameters were set to their default values.

Statistics. GraphPad-PRISM® 8.4.3 software was employed for the analyses of quantitative data, including the two-way ANOVA with Dunnett's test and the Fisher's exact test, corrected for multiple comparisons by false discovery rate (FDR) using a two-stage linear step-up procedure of Benjamini, Krieger and Yekutieli[16].

Supplementary References

1 Giambra, V. *et al.* Epigenetic Restoration of Fetal-like IGF1 Signaling Inhibits Leukemia Stem Cell Activity. *Cell Stem Cell*, doi:10.1016/j.stem.2018.08.018 (2018).

2 Maillard, I. *et al.* Mastermind critically regulates Notch-mediated lymphoid cell fate decisions. *Blood* **104**, 1696-1702, doi:10.1182/blood-2004-02-0514 (2004).

3 Zenatti, P. P. *et al.* Oncogenic IL7R gain-of-function mutations in childhood T-cell acute lymphoblastic leukemia. *Nat Genet* **43**, 932-939, doi:10.1038/ng.924 (2011).

4 Gonzalez-Perez, D. *et al.* Affinity-matured DLL4 ligands as broad-spectrum modulators of Notch signaling. *Nat Chem Biol* **19**, 9-17, doi:10.1038/s41589-022-01113-4 (2023).

5 Wolf, F. A., Angerer, P. & Theis, F. J. SCANPY: large-scale single-cell gene expression data analysis. *Genome Biol* **19**, 15, doi:10.1186/s13059-017-1382-0 (2018).

6 Slovin, S. *et al.* Single-Cell RNA Sequencing Analysis: A Step-by-Step Overview. *Methods Mol Biol* **2284**, 343-365, doi:10.1007/978-1-0716-1307-8_19 (2021).

7 Becht, E. *et al.* Dimensionality reduction for visualizing single-cell data using UMAP. *Nat Biotechnol*, doi:10.1038/nbt.4314 (2018).

8 Ilicic, T. *et al.* Classification of low quality cells from single-cell RNA-seq data. *Genome Biol* **17**, 29, doi:10.1186/s13059-016-0888-1 (2016).

9 Hanzelmann, S., Castelo, R. & Guinney, J. GSVA: gene set variation analysis for microarray and RNA-seq data. *BMC bioinformatics* **14**, 7, doi:10.1186/1471-2105-14-7 (2013).

10 Subramanian, A. *et al.* Gene set enrichment analysis: a knowledge-based approach for interpreting genome-wide expression profiles. *Proc Natl Acad Sci U S A* **102**, 15545-15550, doi:10.1073/pnas.0506580102 (2005).

11 Good, B. M. *et al.* Reactome and the Gene Ontology: Digital convergence of data resources. *Bioinformatics*, doi:10.1093/bioinformatics/btab325 (2021).

12 Traag, V. A., Waltman, L. & van Eck, N. J. From Louvain to Leiden: guaranteeing well-connected communities. *Sci Rep* **9**, 5233, doi:10.1038/s41598-019-41695-z (2019).

13 Wolf, F. A. *et al.* PAGA: graph abstraction reconciles clustering with trajectory inference through a topology preserving map of single cells. *Genome Biol* **20**, 59, doi:10.1186/s13059-019-1663-x (2019).

14 Haghverdi, L., Buttner, M., Wolf, F. A., Buettner, F. & Theis, F. J. Diffusion pseudotime robustly reconstructs lineage branching. *Nature methods* **13**, 845-848, doi:10.1038/nmeth.3971 (2016).

15 Hou, R., Denisenko, E., Ong, H. T., Ramilowski, J. A. & Forrest, A. R. R. Predicting cell-to-cell communication networks using NATMI. *Nature communications* **11**, 5011, doi:10.1038/s41467-020-18873-z (2020).

16 Benjamini, Y., Krieger, A. M. & Yekutieli, D. Adaptive linear step-up procedures that control the false discovery rate. *Biometrika* **93**, 491-507, doi:DOI 10.1093/biomet/93.3.491 (2006).

17 Wang, H. *et al.* Genome-wide analysis reveals conserved and divergent features of Notch1/RBPJ binding in human and murine T-lymphoblastic leukemia cells. *Proc Natl Acad Sci U S A* **108**, 14908-14913, doi:10.1073/pnas.1109023108 (2011).

Supplementary Figures

Fig. S1.

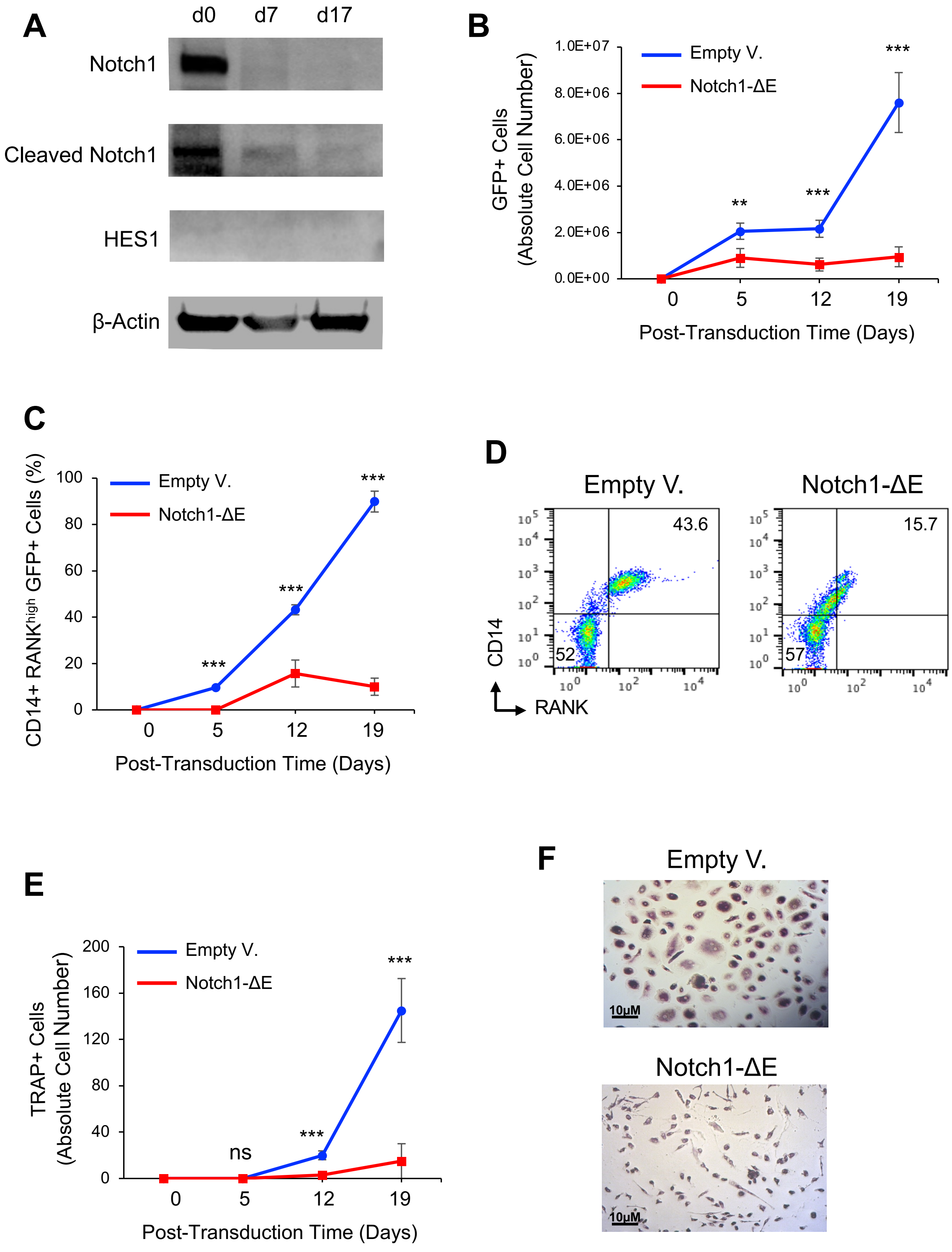

Figure S1. Constitutive activation of Notch1 signaling in total human peripheral blood mononuclear cells (PBMCs) suppresses RANKL-induced osteoclastogenesis.

A) Western blot analysis of total and cleaved intracellular NOTCH1, HES1 and β-Actin as a loading control in total cell lysates of total human PBMCs at day 0 and after *in vitro* growth in serum-containing growth media, supplemented with RANKL (50ng/ml), M-CSF (25ng/ml), TGF-β1(5ng/ml) and dexamethasone (1μM) for 7 and 17 days. The blots were cut prior to hybridisation with antibodies and their full-length blots cannot be provided. The uncropped version of the western blots is reported in Figure S19.

B-C) Flow cytometric analysis of abundance of total GFP+ cells (B) and CD14+RANK^{high}GFP+ cell fraction (C) in total human PBMCs, transduced with lentivectors encoding the active NOTCH1-ΔE isoform or empty vector as control. Abundance of transduced total GFP+ cells (B) and CD14+RANK^{high}GFP+ cell fraction (C) was tracked over time in culture after *in vitro* RANKL stimulation at the indicated time points by flow cytometry. GFP+ alive cells were discriminated using the LIVE/DEAD™ Fixable Near-IR stain. Means ± SD fraction of the initial transduction value are plotted for experiments performed in biological triplicates. ****, p<0.001 (Student's t-test); NS, not significant.*

D) Flow cytometry plots of NOTCH1-ΔE or empty vector transduced cells after 12 days of *in vitro* RANKL stimulation, showing the protein expression of CD14 and RANK cell receptors.

E) Absolute number of TRAP-positive multinucleated cells, derived from the total human PBMCs, lentivirally transduced with the active NOTCH1-ΔE isoform or empty vector as control and subsequently *in vitro* stimulated as described above. Number of TRAP-positive multinucleated cells was quantified microscopically over time in culture as indicated. Means ± SD values are plotted for experiments performed in biological triplicates. **, p<0.05; ***, p<0.001 (Student's t-test); NS, not significant.*

F) Microphotographs of TRAP-positive multinucleated cells, transduced as indicated after 19 days of *in vitro* RANKL stimulation.

Fig. S2.

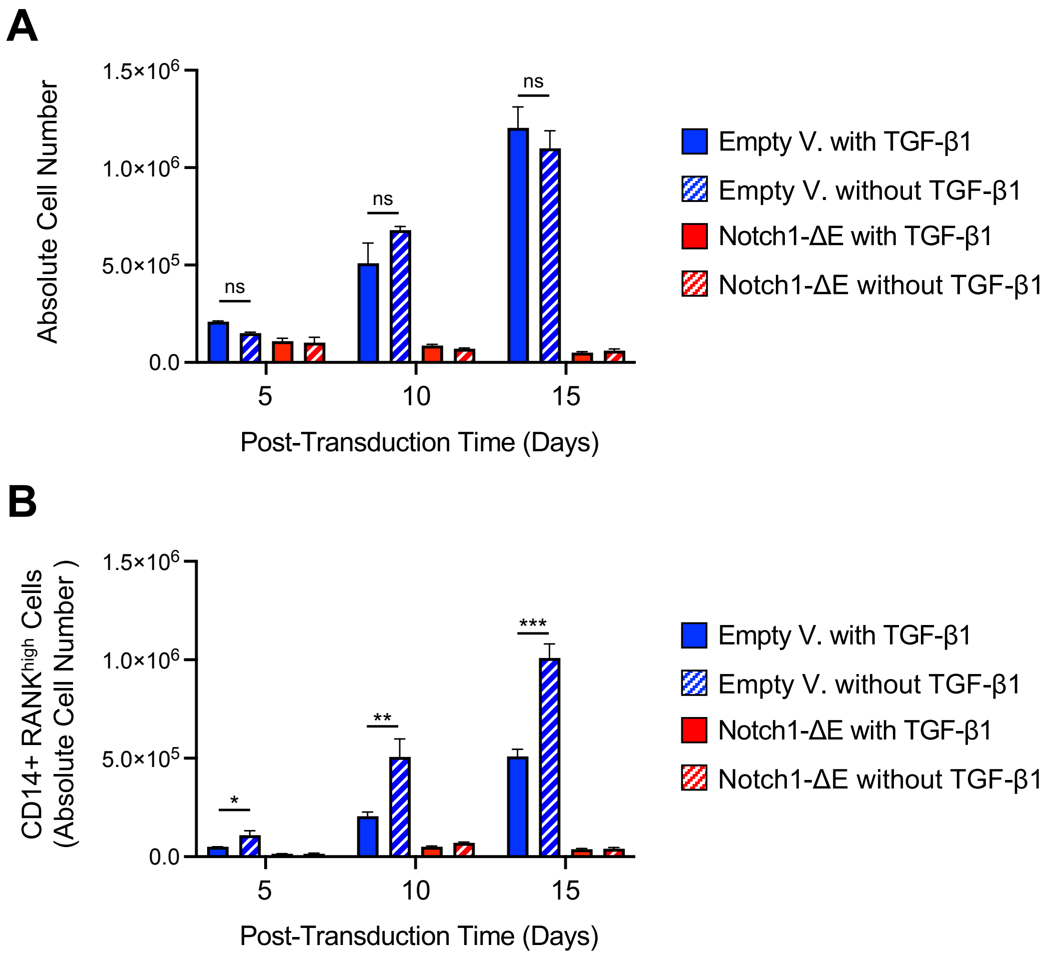

Figure S2. Constitutive activation of Notch1 signaling in human osteoclast precursors suppresses *in vitro* RANKL-induced osteoclastogenesis with or without TGF-β1.

A-B) Flow cytometric analysis of abundance of total cells (A) and CD14+RANK^{high}cell fraction (B) in human CD14+CD16- monocytes, isolated from peripheral blood mononuclear cells (PBMC) and transduced with lentivectors encoding the active NOTCH1-ΔE isoform or empty vector as control. Transduced cells were maintained *in vitro* with or without TGF-β1(25ng/ml) as indicated in serum-containing growth media, supplemented with RANKL (50ng/ml) and M-CSF and seeded at 2*10^6 cells/ml up to 15 days. Alive cells were discriminated by DAPI exclusion by flow cytometry. Means ± SD fractions of the initial transduction values are plotted for

experiments performed in biological triplicates. *, *p<0.05;* **, *p<0.01;* ***, *p<0.001 (Student's t-test); NS, not significant.*

Fig. S3.

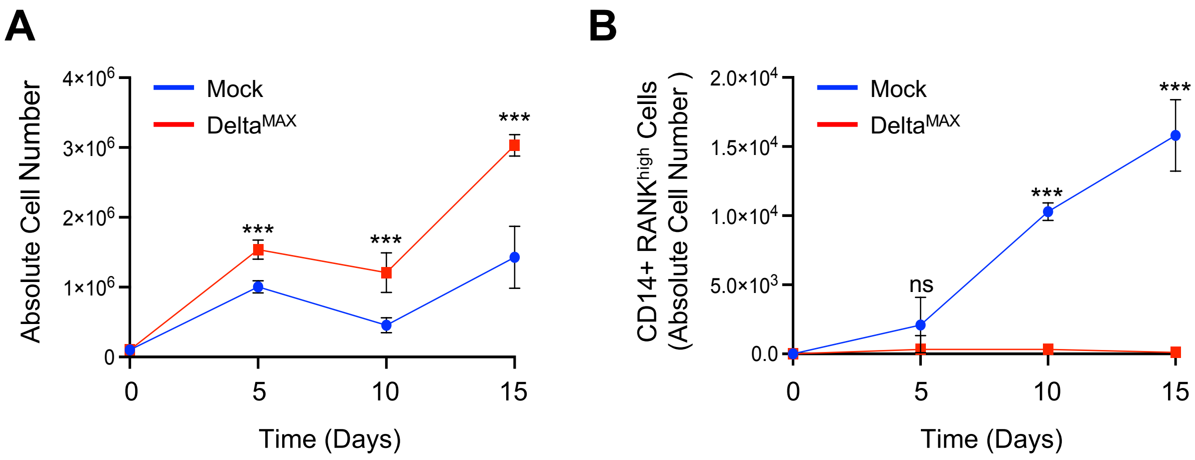

Figure S3. Activation of endogenous Notch signaling in total human peripheral blood mononuclear cells (PBMCs) by human Delta^{MAX} ligand stimulation suppresses RANKL-induced osteoclastogenesis.

A-B) Flow cytometric analysis of abundance of total cells (A) and CD14+RANK^{high}cell fraction (B) in total human PBMCs after *in vitro* RANKL stimulation in presence of μbeads, coated with recombinant human Delta^{MAX} ligand[4] for the activation of endogenous Notch signaling or mouse IgG1 isotype as mock control. Alive cells were discriminated by DAPI exclusion. Means ± SD fractions of the initial transduction values are plotted for experiments performed in biological triplicates. ***, *p<0.001 (Student's t-test); NS, not significant.*

Fig. S4.

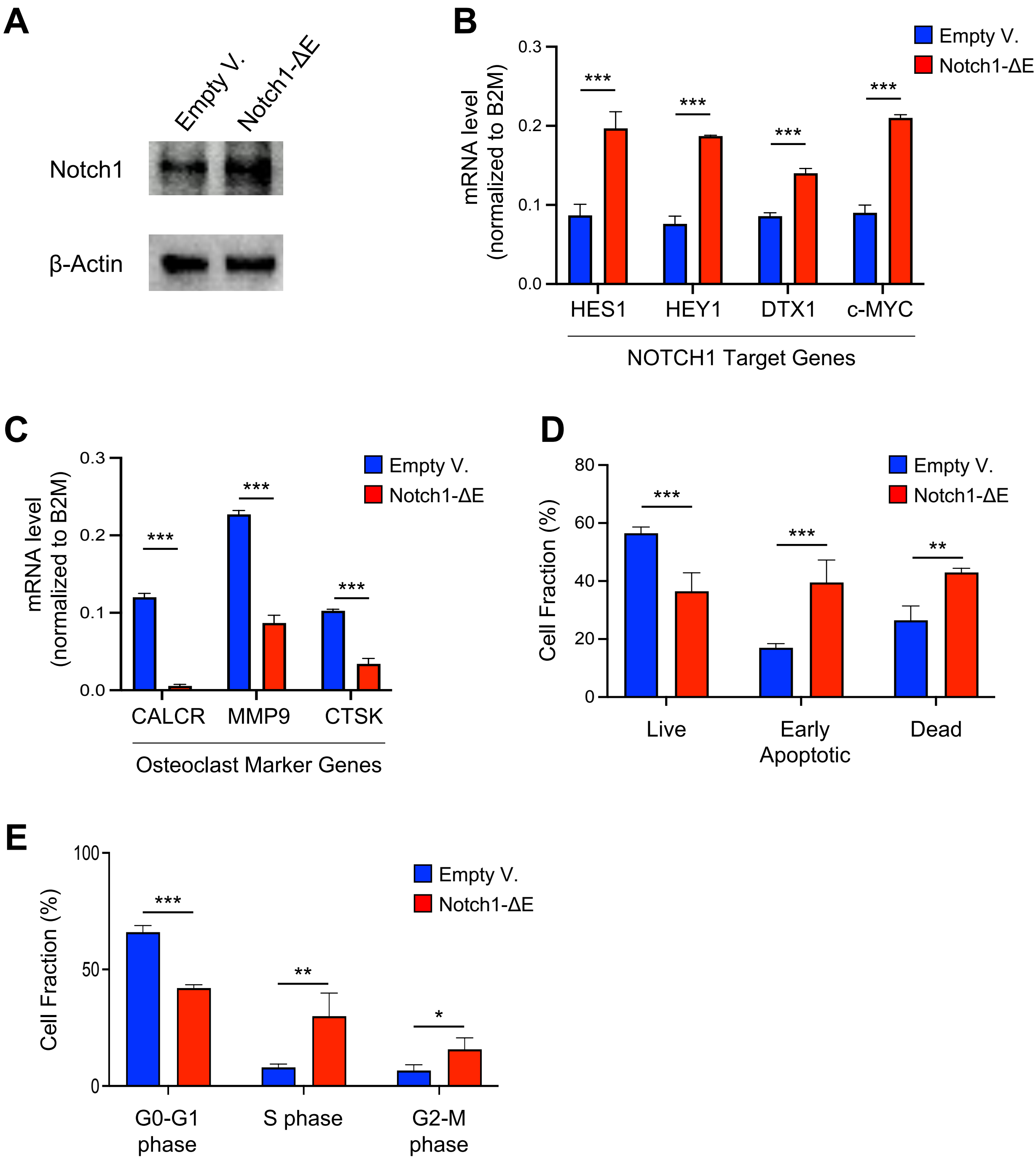
A
Empty V.
Notch1-ΔE
Notch1
β-Actin
B
mRNA level
(normalized to B2M)
0.3
0.2
0.1
0.0
***
***
***
***
HES1
HEY1
DTX1
c-MYC
NOTCH1 Target Genes
Empty V.
Notch1-ΔE
C
mRNA level
(normalized to B2M)
***
***
***
CALCR
MMP9
CTSK
Osteoclast Marker Genes
D
Cell Fraction (%)
80
60
40
20
0
***
***
**
Live
Early
Apoptotic
Dead
E
Cell Fraction (%)
100
50
0
***
**
*
G0-G1
phase
S phase
G2-M
phase

Figure S4. The constitutive activation of Notch1 signaling in human CD14+CD16- monocytes increased cell proliferation, but enforced cell death during RANKL-induced osteoclastogenesis.

A) Western blot analysis of total NOTCH1 and β-Actin as a loading control in total cell lysates of human CD14+CD16- monocytes lentivirally transduced with the active NOTCH1-ΔE/GFP isoform or empty vector as control. Transduced cells were *in vitro* cultured in serum-containing growth media, supplemented with RANKL (50ng/ml), M-CSF (25ng/ml), TGF-β1(5ng/ml) and dexamethasone (1μM) for 7 days before isolation of GFF+ cells by FACS sorting and subsequently protein purification. The uncropped version of the western blots is reported in Figure S19.

B-C) Analysis of real-time PCR gene expression of four NOTCH1 target genes (B) and three osteoclast marker genes (C) in human CD14+CD16- monocytes lentivirally transduced with the active NOTCH1-ΔE/GFP isoform or empty vector as control and subsequently *in vitro* RANKL stimulated for 12 days before RNA extraction. Y-axes, mRNA levels of indicated genes normalized to B2M expression. X-axes, gene symbols. Significance analysis was performed by one-sample t-test.

D) Flow cytometric analysis of early apoptotic cells by AnnexinV binding and 7AAD exclusion in human CD14+CD16- monocytes after transduction with NOTCH1-ΔE/GFP isoform or empty vector as control. Cell subsets were measured after 7 days of *in vitro* RANKL stimulation by flow cytometry. The graphs report the result of two independent experiments performed in biological triplicates. ***, $p<0.01$; ***, $p<0.001$ (Student's t-test).*

E) Cell cycle analysis by BrdU incorporation in human CD14+CD16- monocytes, following transduction with NOTCH1-ΔE/GFP isoform or empty lentivectors as indicated. Transduced cells were measured after 7 days of in vitro growth by flow cytometry. The graphs report the result of two independent experiments performed in biological triplicates. **, $p<0.05$; **, $p<0.01$; ***, $p<0.001$ (Student's t-test).*

Fig. S5.

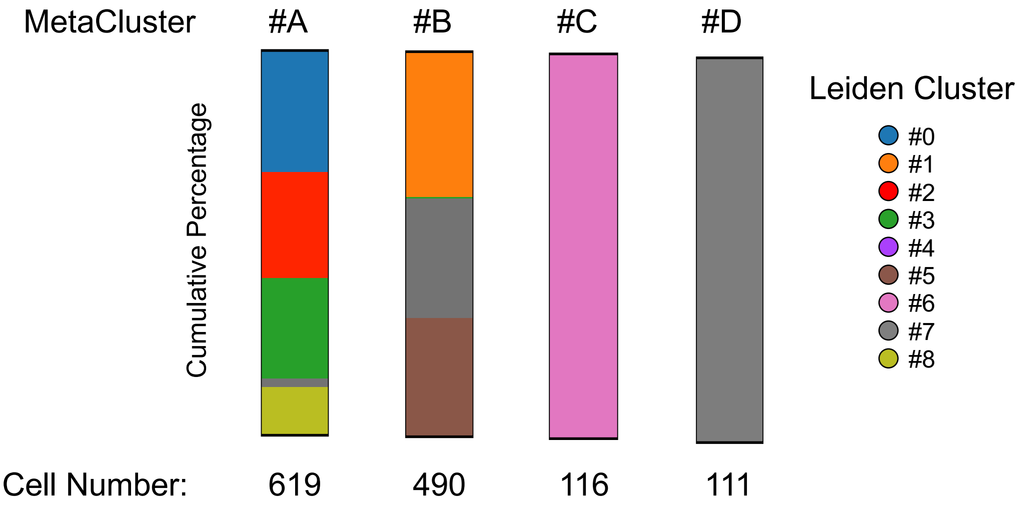

Figure S5. Cumulative cell percentage of Leiden clusters according to each MetaCluster (MC).

Fig. S6.

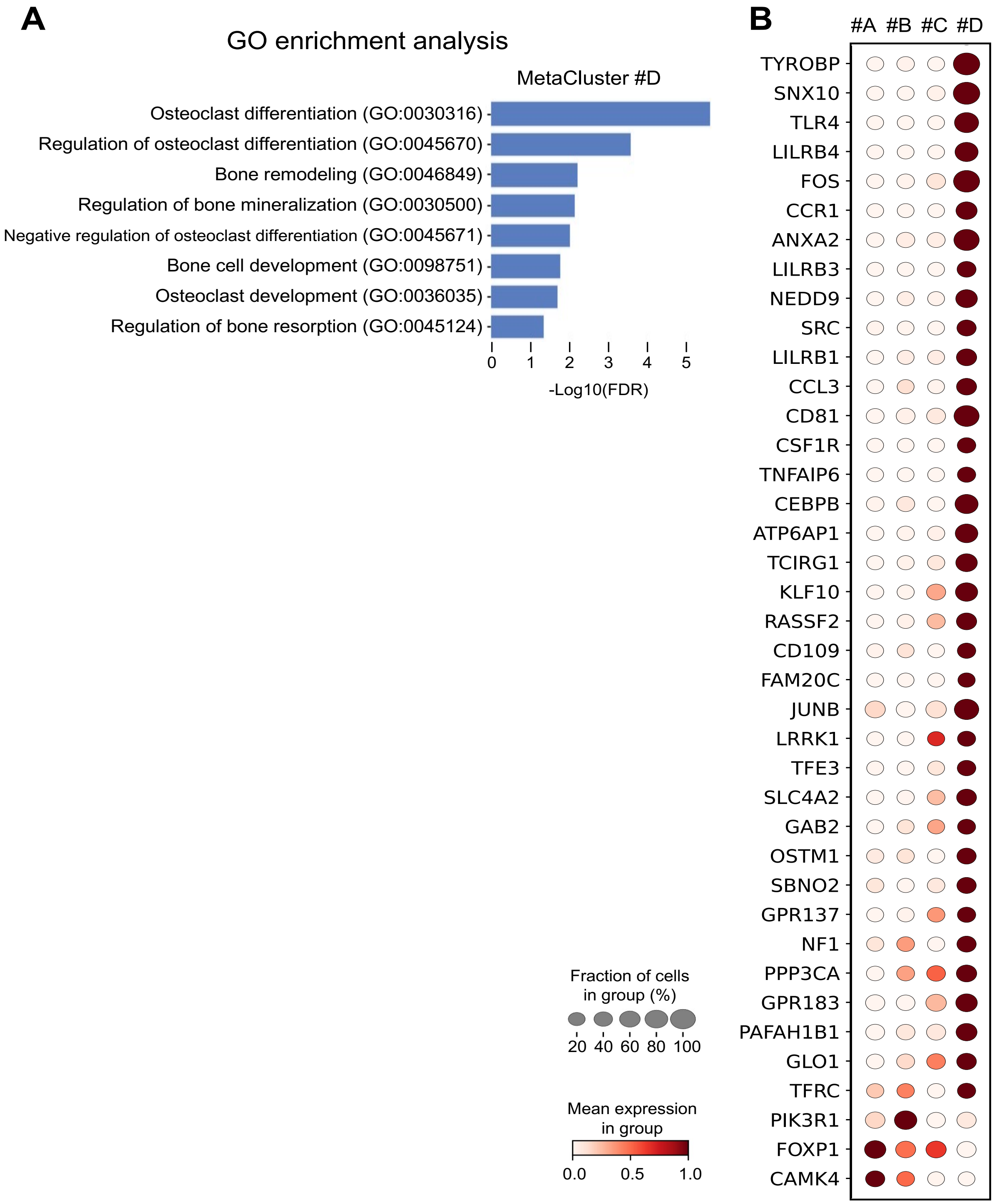

A
GO enrichment analysis
MetaCluster #D
Osteoclast differentiation (GO:0030316)
Regulation of osteoclast differentiation (GO:0045670)
Bone remodeling (GO:0046849)
Regulation of bone mineralization (GO:0030500)
Negative regulation of osteoclast differentiation (GO:0045671)
Bone cell development (GO:0098751)
Osteoclast development (GO:0036035)
Regulation of bone resorption (GO:0045124)
0 1 2 3 4 5
-Log10(FDR)
Fraction of cells in group (%)
20 40 60 80 100
Mean expression in group
0.0 0.5 1.0
B
#A #B #C #D
TYROBP
SNX10
TLR4
LILRB4
FOS
CCR1
ANXA2
LILRB3
NEDD9
SRC
LILRB1
CCL3
CD81
CSF1R
TNFAIP6
CEBPB
ATP6AP1
TCIRG1
KLF10
RASSF2
CD109
FAM20C
JUNB
LRRK1
TFE3
SLC4A2
GAB2
OSTM1
SBNO2
GPR137
NF1
PPP3CA
GPR183
PAFAH1B1
GLO1
TFRC
PIK3R1
FOXP1
CAMK4

Figure S6. Genes of osteoclast differentiation and development are enriched in cells of MC-#D cluster by Gene Ontology (GO) enrichment analysis.

A) Top significantly enriched GO biological processes related to the highly differentially expressed genes in cells of MC-#D cluster.

B) Dot plot representation of genes, related to osteoclast differentiation (GO:0030316) and regulation (GO:0045670) GO processes and significantly differentially expressed (adjusted p-value <0.05) among the MC-#D subset and other indicated MC clusters.

Fig. S7.

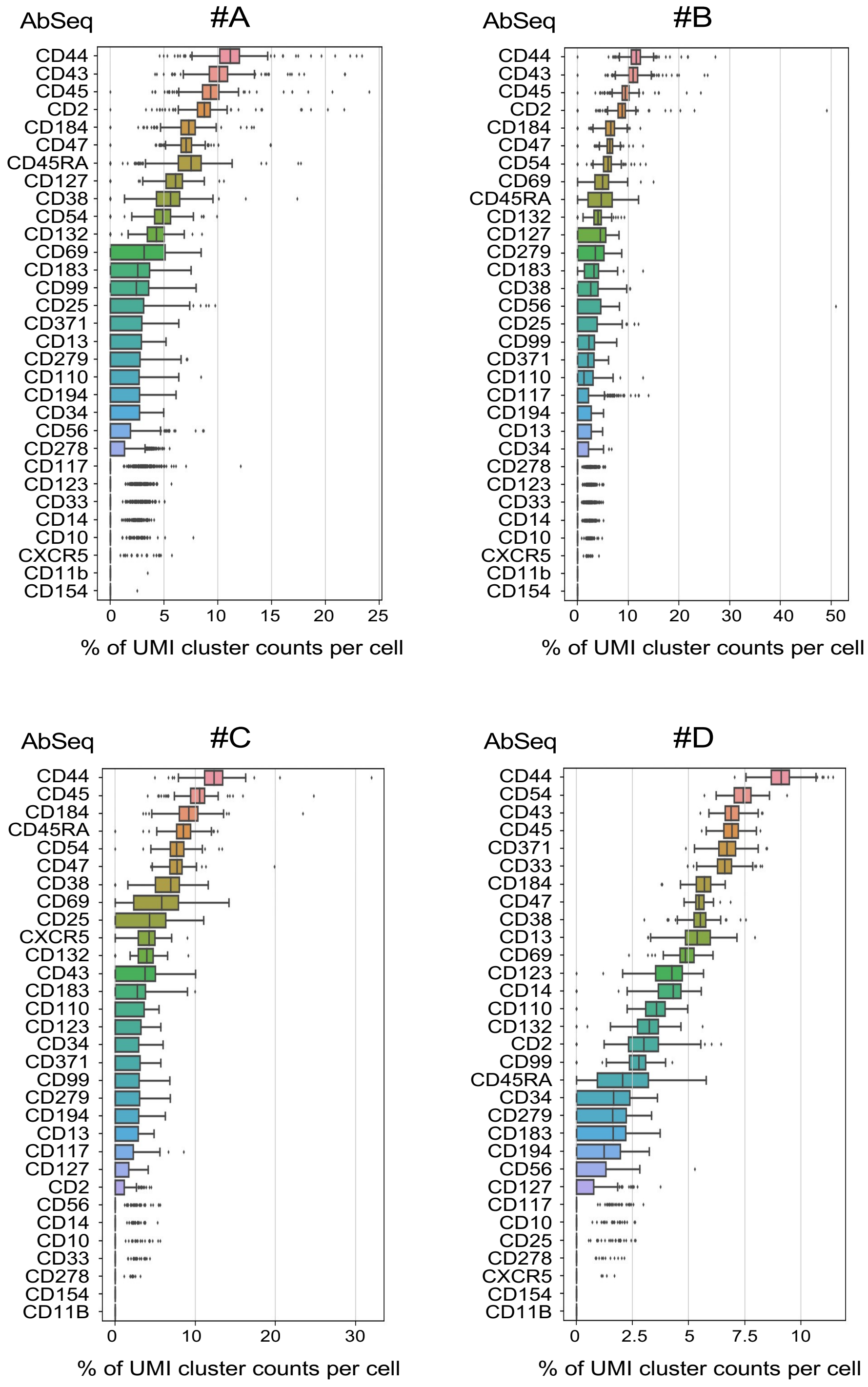

Figure S7. Expression level of indicated oligo-conjugated antibodies in each considered MetaCluster (MC) by AbSeq assay.

The boxplots report the percentage of UMI counts of indicated oligo-conjugated antibody in each cell, across the different MetaClusters (MC).

Fig. S8.

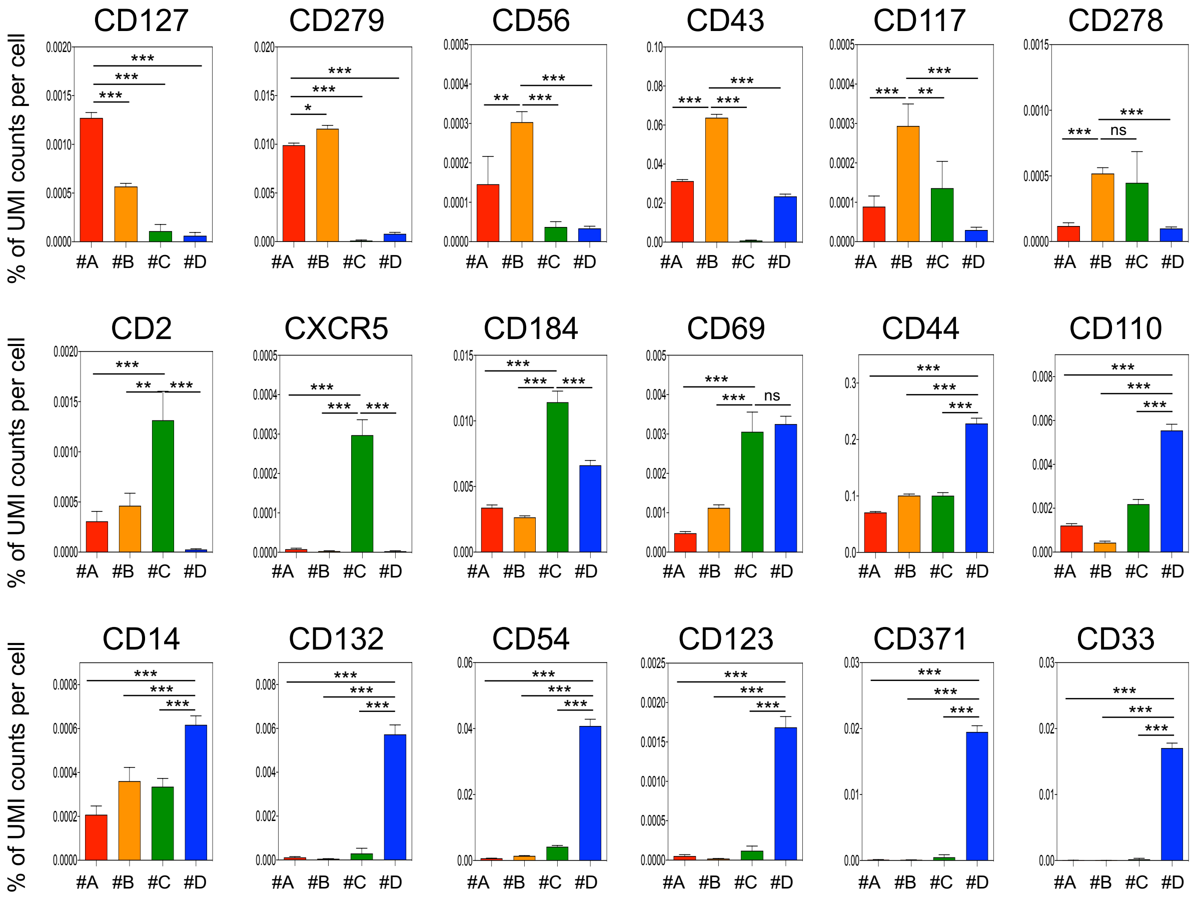

Figure S8. Comparison of expression level of indicated oligo-conjugated antibodies among the four MetaClusters (MC).

The percentage of UMI counts of indicated oligo-conjugated antibody in each cell, across the different MetaClusters (MC) is indicated in the boxplots. *ns, not significant; *, p<0.05; **, p<0.01; ***, p<0.001 (Two-way ANOVA with Dunnett's test, comparing the indicated cluster mean with the other values).*

Fig. S9.

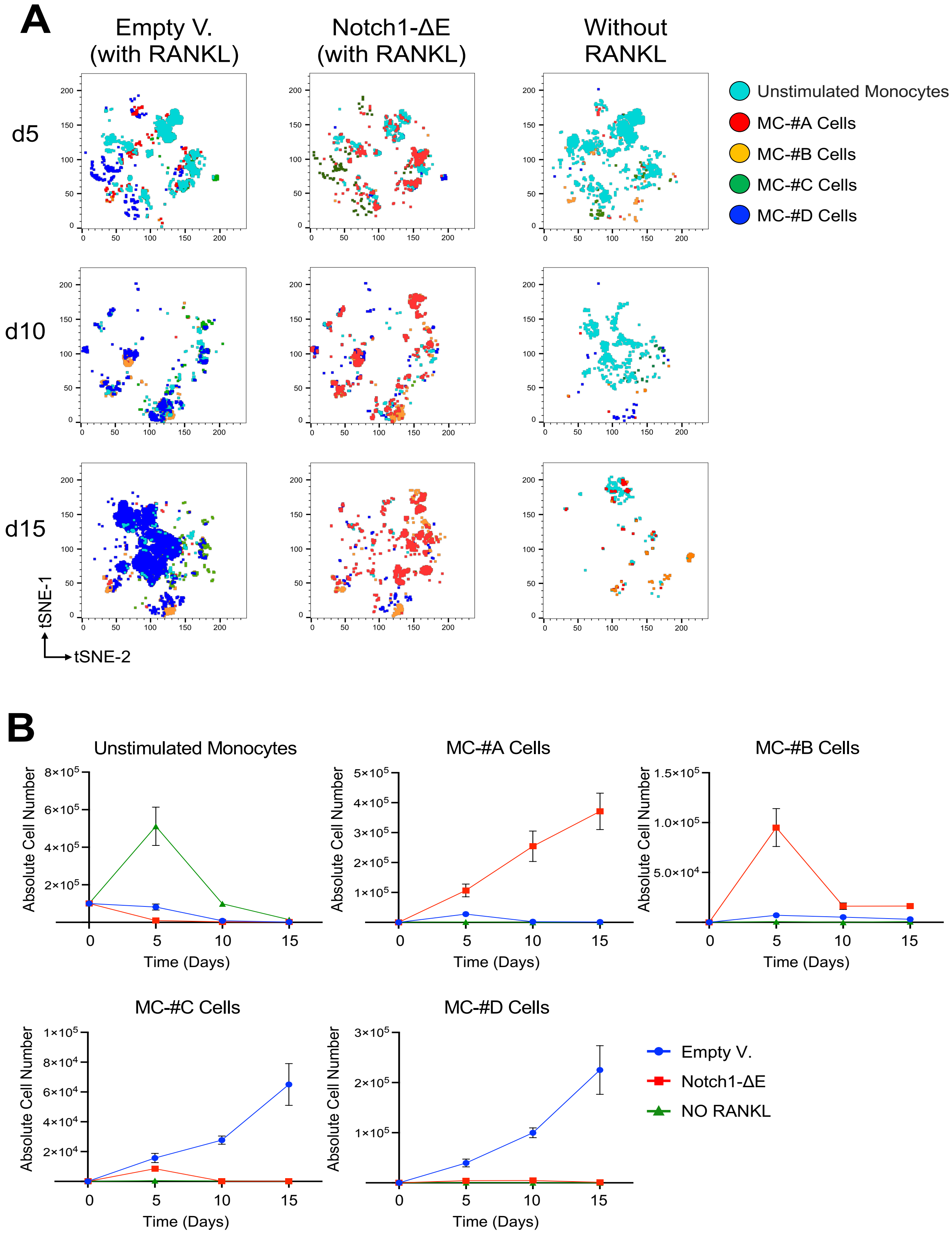
A
Empty V. (with RANKL)
Notch1-ΔE (with RANKL)
Without RANKL
d5
d10
d15
tSNE-1
tSNE-2
Unstimulated Monocytes
MC-#A Cells
MC-#B Cells
MC-#C Cells
MC-#D Cells
B
Unstimulated Monocytes
MC-#A Cells
MC-#B Cells
MC-#C Cells
MC-#D Cells
Absolute Cell Number
Time (Days)
Empty V.
Notch1-ΔE
NO RANKL

Figure S9. Multiparameter flow cytometry assessment of MetaClusters identified by scRNA-Seq profile during *in vitro* human osteoclagenesis.

A) tSNE plots based on the multiparameter flow cytometry assessment of human CD14+CD16- monocytes, transduced with NOTCH1-ΔE lentiviruses or empty vector as control with/without *in vitro* RANKL stimulation as indicated. Cells are colored based on their assigned MetaCluster (MC) as determined by the Leiden graph-clustering method (resolution=0.4). Unstimulated monocytes are also reported in sky-blue. Panel of cell surface markers and fluorophore-conjugated antibodies used in the multiparameter flow cytometry assessment as well as the gating strategies are specified in Supplementary Table 6 and 7, respectively.

B) Flow cytometric analysis of abundance of unstimulated monocytes as well as of different cell subpopulations of indicated MetaCluster (MC) in human CD14+CD16- monocytes, transduced with NOTCH1-ΔE lentiviruses or empty vector as control with/without *in vitro* RANKL stimulation. Means ± SD fractions of the initial transduction values are plotted for experiments performed in biological triplicates.

Fig. S10.

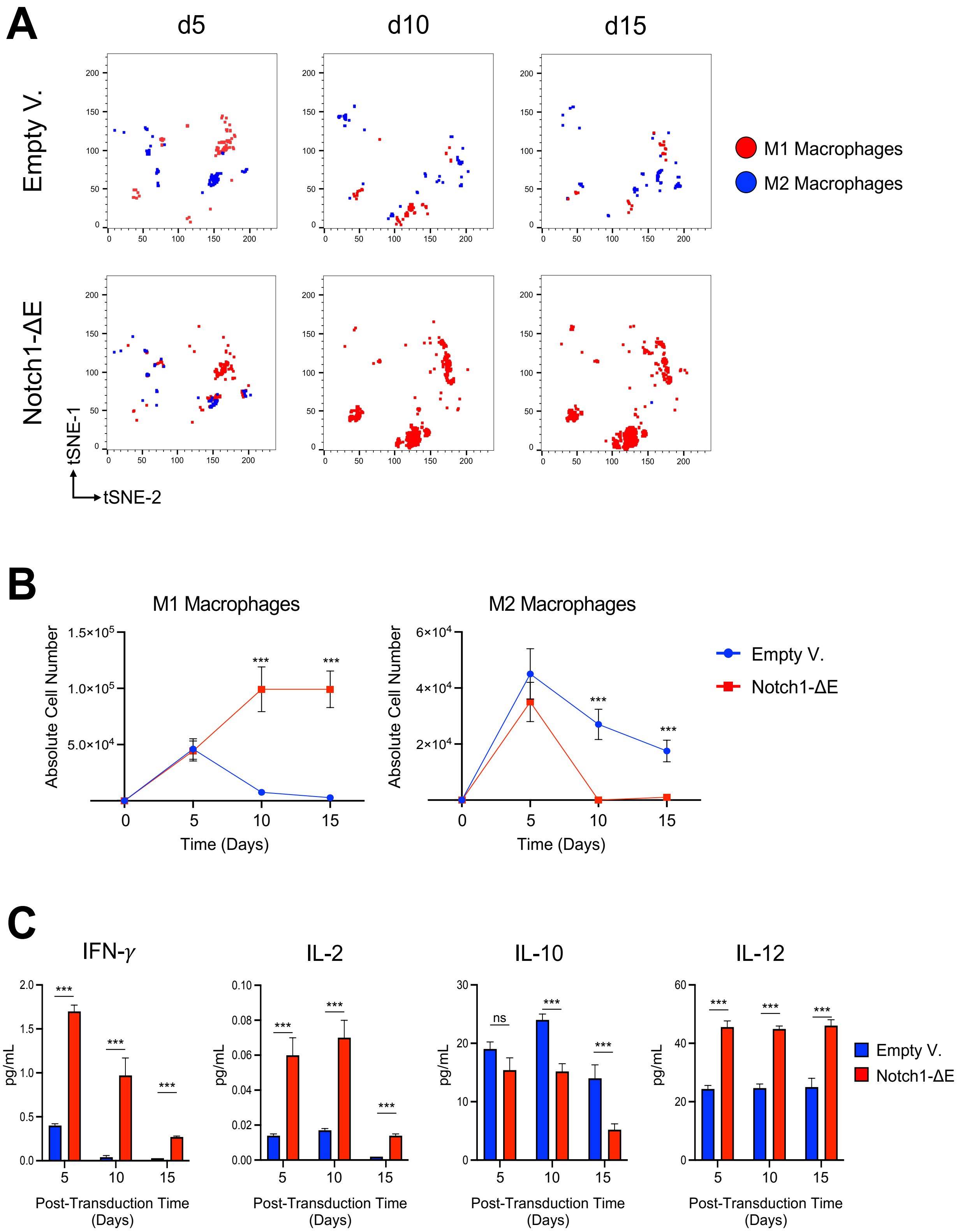

A
d5
d10
d15
Empty V.
Notch1-ΔE
tSNE-1
tSNE-2
M1 Macrophages
M2 Macrophages
B
M1 Macrophages
M2 Macrophages
Absolute Cell Number
Time (Days)
Empty V.
Notch1-ΔE
C
IFN-γ
IL-2
IL-10
IL-12
pg/mL
Post-Transduction Time (Days)
Empty V.
Notch1-ΔE

Figure S10. Multiparameter flow cytometry assessment of M1 and M2 macrophages derived from human CD14+CD16- monocytes, transduced with NOTCH1-ΔE lentiviruses or empty vector as control during *in vitro* human osteoclagenesis.

A) tSNE plots based on the multiparameter flow cytometry assessment of M1 and M2 macrophages derived from human CD14+CD16- monocytes, transduced with NOTCH1-ΔE lentiviruses or empty vector as control after *in vitro* RANKL stimulation as indicated. M1 and M2 macrophages are colored in red and blue, respectively. Panel of cell surface markers and fluorophore-conjugated antibodies used in the multiparameter flow cytometry assessment as well as the gating strategies are specified in Supplementary Table 6 and 7, respectively.

B) Flow cytometric analysis of abundance of M1 and M2 macrophages derived from human CD14+CD16- monocytes, transduced with NOTCH1-ΔE lentiviruses or empty vector as control after *in vitro* RANKL stimulation. Means ± SD fractions of the initial transduction values are plotted for experiments performed in biological triplicates. ****, p<0.001 (Student's t-test).*

C) Concentration level of soluble human cytokines and chemokines in the in the conditioned media derived from human CD14+CD16- monocytes, transduced with NOTCH1-ΔE lentiviruses or empty vector as control after RANKL-stimulation at the indicated time points by flow cytometry analysis based on MACSPlex Capture Beads (Miltenyi Biotec Inc.). Through this assay, GM-CSF, IFN-α, IFN-γ, IL-2 IL-4, IL-5, IL-6, IL-9, IL-10, IL-12, IL-17 and TNF-α soluble analytes were analyzed using a cocktail of various fluorescently labelled bead populations, each coated with specific antibodies. The only analytes with a concentration over the level of detection are reported in the plots. *ns, not significant; ***, p<0.001 (two-tailed unpaired Welch's t-test).*

Fig. S11.

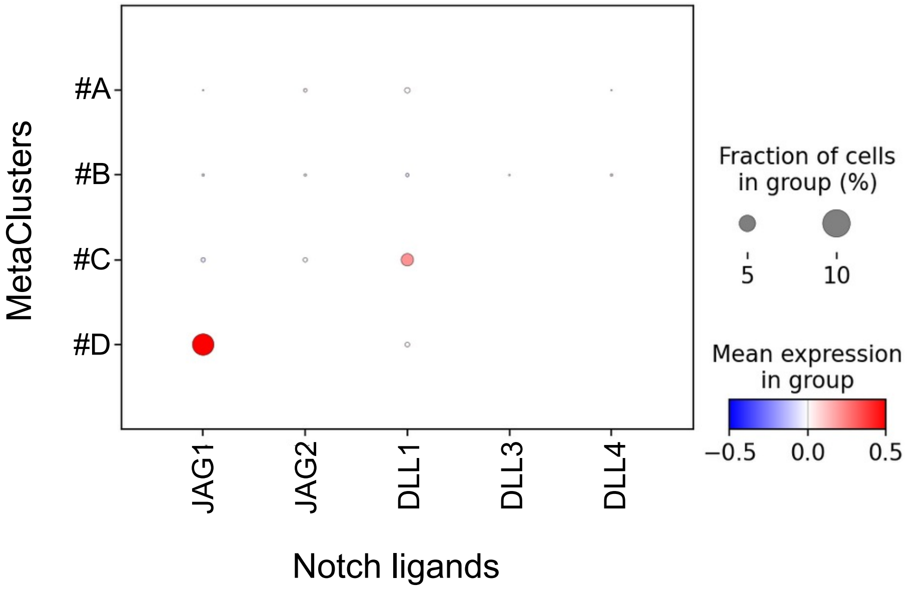

Figure S11. Dot plot of genes encoding five different Notch ligands (JAG1, JAG2, DLL1, DLL3 and DLL4) in the cells of MetaClusters indicated by scRNA-Seq profile.

Fig. S12.

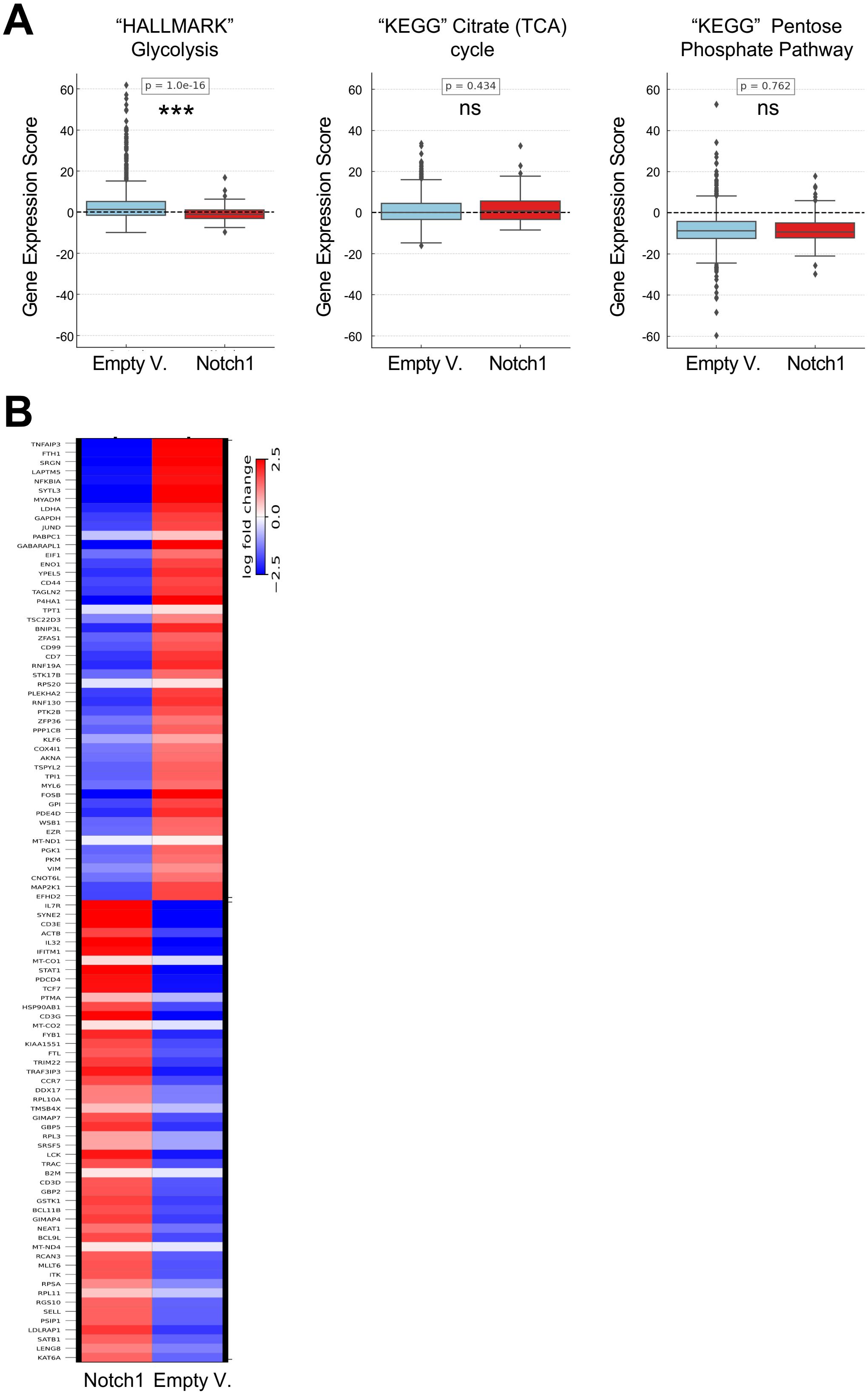
A
"HALLMARK" Glycolysis
"KEGG" Citrate (TCA) cycle
"KEGG" Pentose Phosphate Pathway
Gene Expression Score
p = 1.0e-16
***
p = 0.434
ns
p = 0.762
ns
60
40
20
0
-20
-40
-60
Empty V.
Notch1
B
TNFAIP3
FTH1
SRGN
LAPTM5
NFKBIA
SYTL3
MYADM
LDHA
GAPDH
JUND
PABPC1
GABARAPL1
EIF1
ENO1
YPEL5
CD44
TAGLN2
P4HA1
TPT1
TSC22D3
BNIP3L
ZFAS1
CD99
CD7
RNF19A
STK17B
RPS20
PLEKHA2
RNF130
PTK2B
ZFP36
PPP1CB
KLF6
COX4I1
AKNA
TSPYL2
TPI1
MYL6
FOSB
GPI
PDE4D
WSB1
EZR
MT-ND1
PGK1
PKM
VIM
CNOT6L
MAP2K1
EFHD2
IL7R
SYNE2
CD3E
ACTB
IL32
IFITM1
MT-CO1
STAT1
PDCD4
TCF7
PTMA
HSP90AB1
CD3G
MT-CO2
FYB1
KIAA1551
FTL
TRIM22
TRAF3IP3
CCR7
DDX17
RPL10A
TMSB4X
GIMAP7
GBP5
RPL3
SRSF5
LCK
TRAC
B2M
CD3D
GBP2
GSTK1
BCL11B
GIMAP4
NEAT1
BCL9L
MT-ND4
RCAN3
MLLT6
ITK
RPSA
RPL11
RGS10
SELL
PSIP1
LDLRAP1
SATB1
LENG8
KAT6A
log fold change
2.5
0.0
−2.5
Notch1
Empty V.

Figure S12. The constitutive activation of Notch1 signaling in human CD14+CD16- monocytes alters distinct metabolic gene signatures and gene markers.

A) Boxplots of gene expression scores for the "Glycolysis" (cod. M5937, HALLMARK), "Citrate (TCA) cycle" (cod. M3985, KEGG) and "Pentose Phosphate Pathway" (cod. M1386, KEGG) human gene sets as identified at the following link https://www.gsea-msigdb.org/ and indicated in human CD14+CD16- monocytes transduced with lentivectors encoding the active NOTCH1-ΔE isoform or empty vector as control for the scRNA-Seq assay. *ns = not significant; ***, p<0.001 (Mann–Whitney U test).*

B) Heatmap of gene expression scRNA-Seq data for the top 50 genes, related to Glycolysis" (cod. M5937, HALLMARK) gene set and significantly differentially expressed (adjusted p-value <0.05) among the NOTCH1-ΔE-transduced vs. control cells.

Fig. S13.

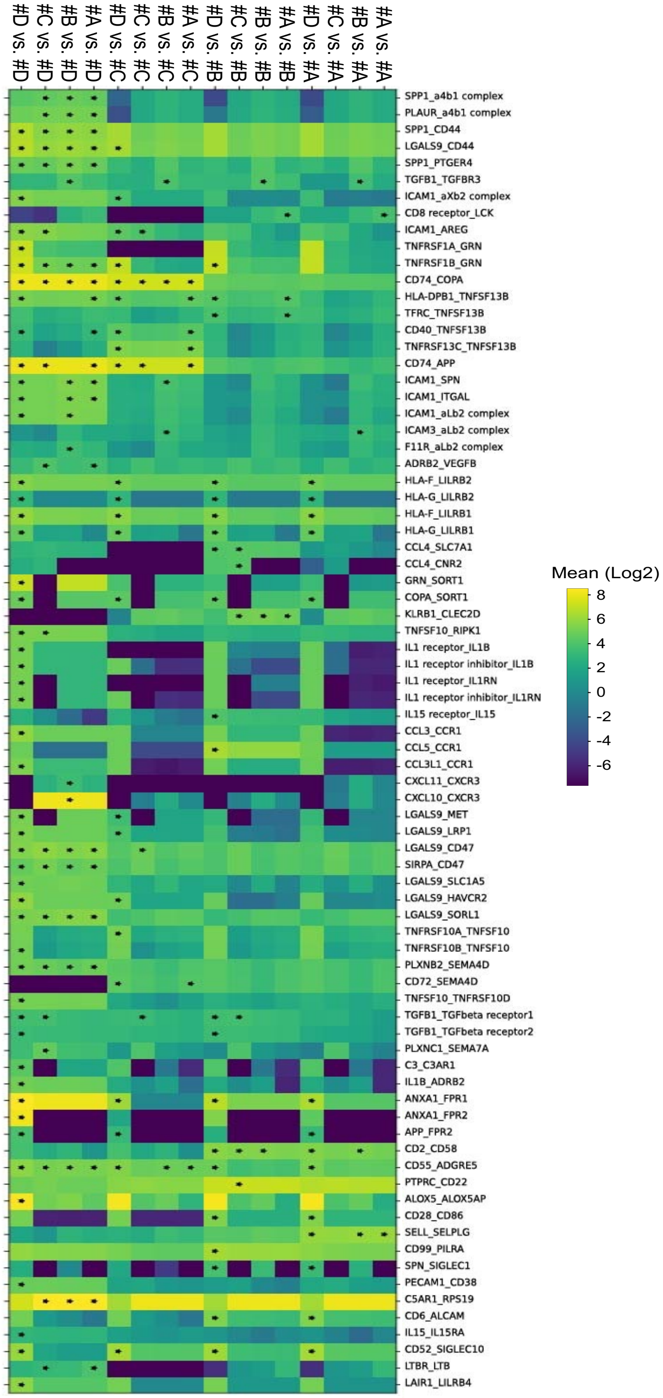

Figure S13. Heatmap of cluster-cluster interactions.

Heatmap represents the average expression of ligand-receptor pairs (in rows) among all combinations of cluster pairs (in columns). Highly significant ligand-receptor interactions are marked with stars. Average expressions are plotted in logarithmic scale.

Fig. S14.

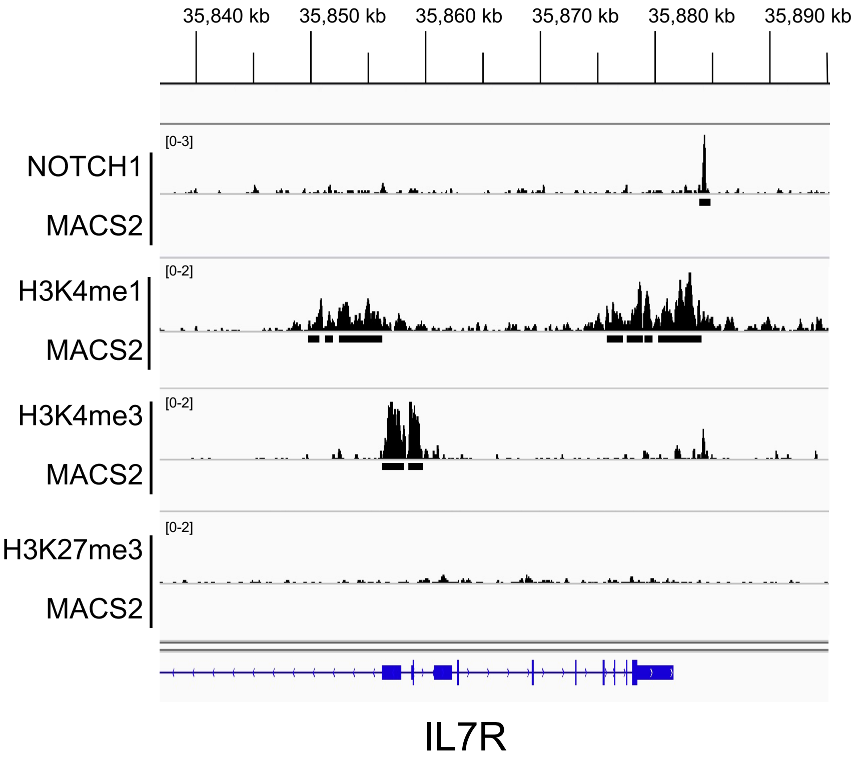

Figure S14. NOTCH1 binds the promoter region of the human IL7R locus.

ChIPseq tracks over the human IL7R 5' region from the human T-ALL cell line CUTLL1. Peaks of aligned reads over the IL7R locus are shown along with MACS2 peak calls (p-value ≤ 0.05). The active genomic region identified by the active enhancer markers, H3K24me1 and H3K4me3. Data are replotted from GEO: GSE29600[17].

Fig. S15.

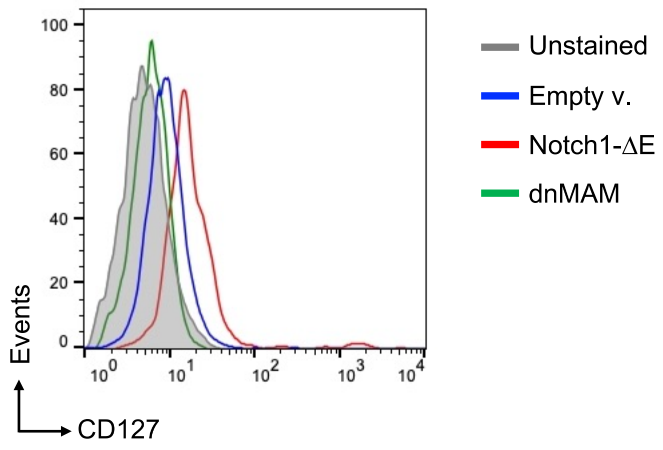

Figure S15. IL7R expression is directly modulated by NOTCH1 in osteoclast progenitors. Protein expression level of IL7R/CD127 cell marker by flow cytometric analysis in human CD14+CD16- monocytes transduced by lentivirus encoding NOTCH1-ΔE isoform, a dominant-negative form of Mastermind-like protein 1 (dnMAM) or empty vector as control. Cells were assessed after three days of *in vitro* RANKL stimulation by flow cytometry.

Fig. S16.

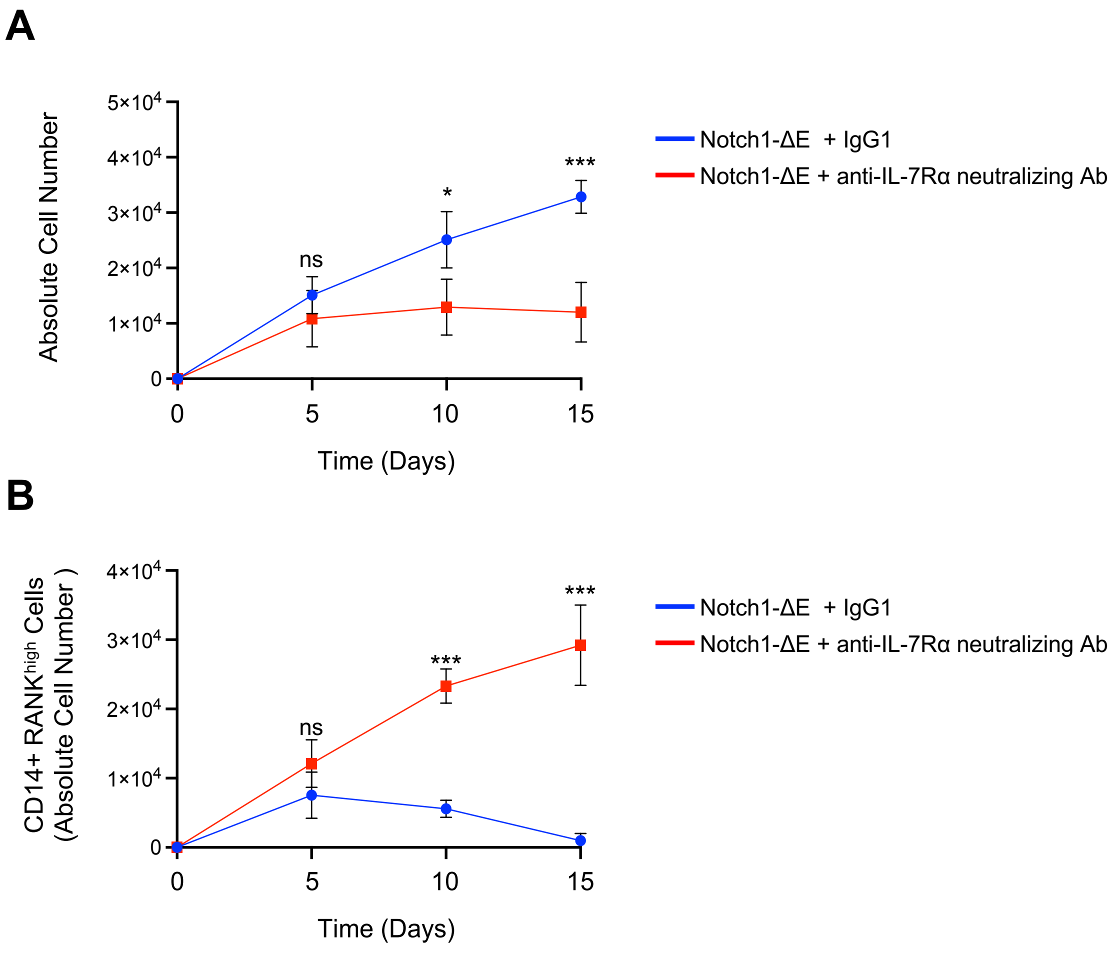

Figure S16. The inhibition of RANKL-induced osteoclastogenesis due to constitutive activation of Notch1 signaling in human osteoclast precursors is abolished *in vitro* upon the addition of a neutralizing antibody against IL-7R.

A-B) Flow cytometric analysis of abundance of total cells (A) and CD14+RANK^{high}cell fraction (B) in human CD14+CD16- monocytes, isolated from peripheral blood mononuclear cells (PBMC) and transduced with lentivectors encoding the active NOTCH1-ΔE isoform after *in vitro* RANKL stimulation in presence of neutralizing antibody (1 μg/ml) against IL-7R (CD127 Monoclonal Antibody (A7R34), cat. #14-1271-82, ThermoFisher) or mouse IgG1 isotype as mock control. Alive cells were discriminated by DAPI exclusion. Means ± SD fractions of the initial transduction values are plotted for experiments performed in biological triplicates. **, $p<0.05$; ***, $p<0.001$ (Student's t-test); NS, not significant.*

Fig. S17.

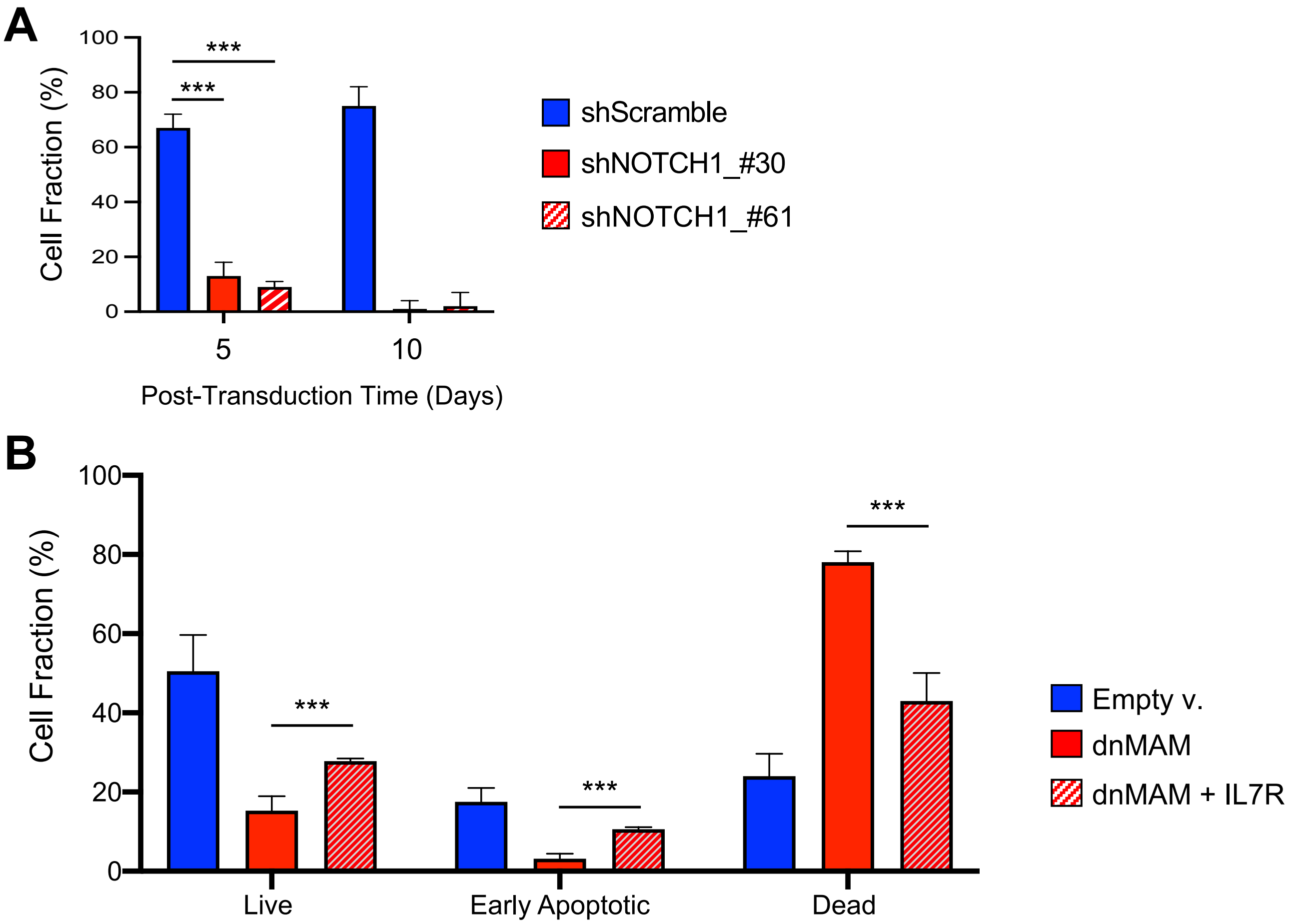

Figure S17. Constitutive activation of IL7R signaling rescues the apoptotic phenotype induced by the block of Notch1 signaling pathway.

A) Flow cytometric analysis of abundance of shRNA-transduced mTag2BFP+ cell fraction. Human CD14+CD16- monocytes were isolated from peripheral blood mononuclear cells (PBMC) and transduced with shRNA/mTag2BFP lentiviral constructs against NOTCH1 or scramble control as indicated, FACS sorted and *in vitro* RANKL stimulated. mTag2BFP + alive cells were measured at the indicated time points by flow cytometry for DRAQ7 exclusion. The graphs report the result of three independent biological experiments performed in triplicate. ****, p<0.001 (Two-way ANOVA with Dunnett's test, comparing the sh-scramble control mean with the other values).*

B) Flow cytometric analysis of early apoptotic cells by AnnexinV binding and 7AAD exclusion in human CD14+CD16- monocytes after transduction with dnMAM alone (dnMAM) or in combination with IL7R_P2mut (dnMAM+IL7R) construct, harboring the p.Thr244_Ile245insCysProThr mutation to induce constitutive signaling (Zenatti PP. et al., Nat Genet., 2011). Cells transduced with empty lentivectors were also included as control. Cell subsets were measured after three days of *in vitro* RANKL stimulation by flow cytometry. The graphs report the result of two independent experiments performed in biological triplicates. ********, p<0.001 (Student's t-test).*

Fig. S18.

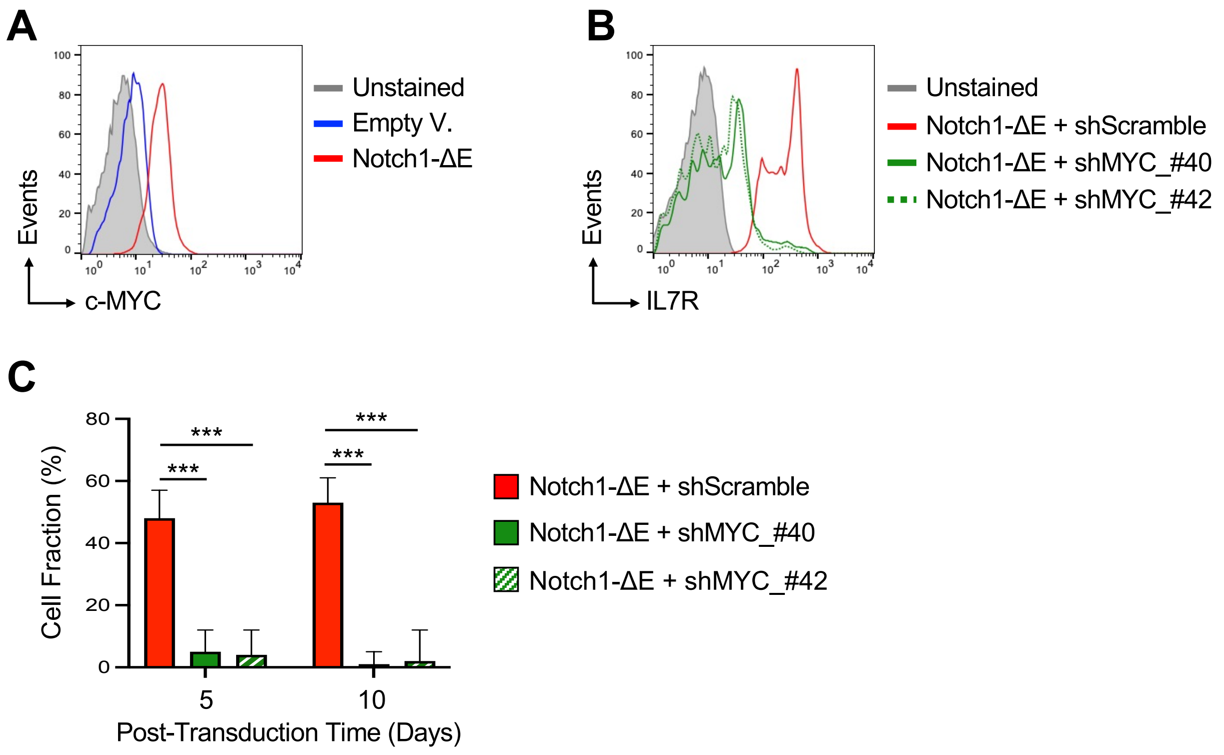

Figure S18. The NOTCH1 target gene, c-MYC modulates the expression of IL7R receptor and is crucial mediator of Notch1 signaling pathway.

A) Protein expression level of c-MYC by flow cytometry in human CD14+CD16- monocytes transduced with NOTCH1-ΔE or empty (EV) lentivectors with GFP selection marker. GFP+ cells were measured after five days from the transduction by flow cytometry.

B) Protein expression level of IL7R by flow cytometry in human CD14+CD16- monocytes, transduced with lentivectors encoding the active NOTCH1-ΔE/GFP isoform in combination with shRNA/mTag2BFP lentiviral constructs against c-MYC or scramble control as indicated and *in vitro* RANKL stimulated. GFP+ mTag2BFP+ alive cells were measured after seven days from the transduction and identified for DRAQ7 exclusion by flow cytometry.

C) Flow cytometric analysis of abundance of shRNA-transduced mTag2BFP+GFP+ cell fraction. Human CD14+CD16- monocytes were isolated from peripheral blood mononuclear cells (PBMC) and transduced with lentivectors encoding the active NOTCH1-ΔE/GFP isoform in combination with shRNA/mTag2BFP lentiviral constructs against c-MYC or scramble control as indicated, FACS sorted and *in vitro* RANKL stimulated. mTag2BFP+GFP+ alive cells were measured at the indicated time points by flow cytometry for DRAQ7 exclusion. The graphs report the result of three independent biological experiments performed in triplicate. ****, p<0.001 (Two-way ANOVA with Dunnett's test, comparing the sh-scramble control mean with the other values).*

Fig. S19.

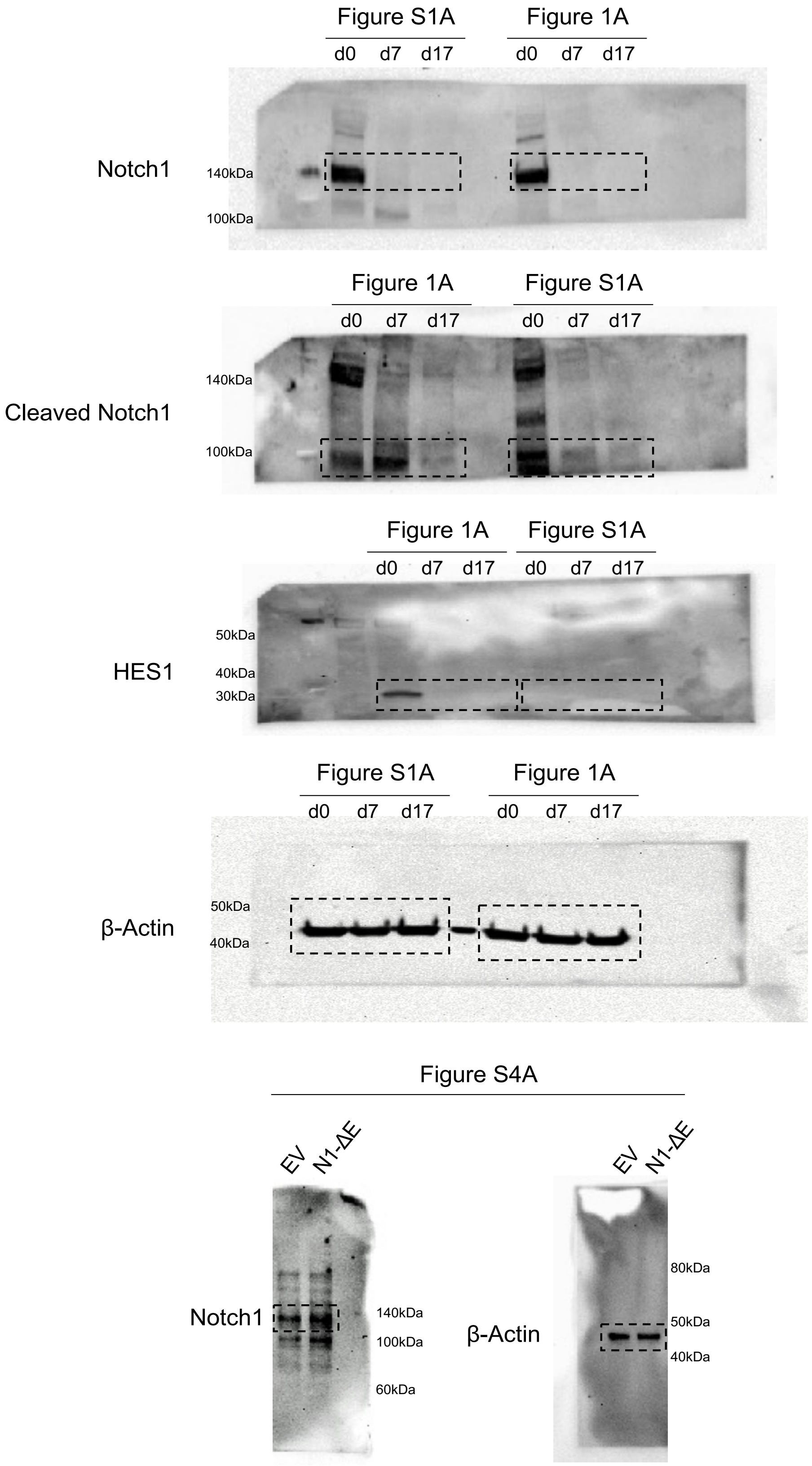
Figure S1A
Figure 1A
d0
d7
d17
Notch1
140kDa
100kDa
Figure 1A
Figure S1A
Cleaved Notch1
HES1
50kDa
40kDa
30kDa
Figure S1A
Figure 1A
β-Actin
Figure S4A
EV
N1-ΔE
60kDa
80kDa

Figure S19. Full length uncropped original western blots related to Figures 1A, S1A and S4A.
The blots of Figures 1A and S1A were cut prior to hybridisation with antibodies and their full-length blots cannot be provided.

Table. S1.

Marker	Ab Clone	BD Code
CD10	HI10A	940045
CD11b	ICRF44	940266
CD110	1.6.1	940302
CD117 (c-KIT)	104D2	940250
CD123 (IL-3RA)	TG3	940020
CD127 (IL-7Rα)	HIL-7R-M21	940012
CD13	WM15	940044
CD132 (IL2RG)	TUGh4	940230
CD14	MPHIP9	940005
CD154 (CD40L)	TRAP1	940053
CD183 (CXCR3)	1C6/CXCR3	940030
CD184 (CXCR4)	12G5	940056
CD185 (CXCR5)	RF8B2	940042
CD194 (CCR4)	1G1	940047
CD2	RPA-2.10	940046
CD25 (IL-2R)	2A3	940009
CD278 (ICOS)	DX29	940043
CD279 PD1	EH12.1	940015
CD33	P67.6	940255
CD34	581	940021
CD371	50C1	940212
CD38	HIT2	940013
CD43	1G10	940278
CD44	515	940364
CD45	HI30	940002
CD45RA	HI100	940011
CD47 (IAP)	B6H12	940082
CD54 (ICAM1)	HA58	940072
CD56	NCAM16.2	940007
CD69	FN50	940019
CD99	TU12	940214

Table S1. Panel of 31 cell surface markers, recognized by the oligo-conjugated antibodies in the Abseq assay.

Table. S2.

MetaCluster #A vs. Others

Gene Set	Size	ES	NES	NOM. p-value	FDR q-value	FWER p-value	Rank at Max	Leading Edge
KEGG_Hematopoietic Cell Lineage	24	0.37	2.18	0.000	0.064	0.062	1170	tags=83%, list=47%, signal=155%
KEGG_JAK STAT Signaling Pathway	36	0.31	2.12	0.000	0.040	0.079	422	tags=47%, list=17%, signal=56%
KEGG_Notch Signaling Pathway	40	0.26	1.96	0.014	0.056	0.212	790	tags=58%, list=32%, signal=83%
KEGG_WNT Signaling Pathway	57	0.20	1.76	0.019	0.107	0.536	785	tags=51%, list=31%, signal=56%
KEGG_Focal Adhesion	36	0.22	1.49	0.074	0.342	0.941	785	tags=53%, list=31%, signal=96%
KEGG_NOD Like Receptor Signaling Pathway	23	0.23	1.28	0.171	0.651	1.000	1287	tags=74%, list=52%, signal=151%
KEGG_Oxidative Phosphorylation	37	0.18	1.26	0.192	0.658	1.000	843	tags=51%, list=34%, signal=76%
HALLMARK_MYC Targets V1	30	0.30	1.94	0.008	0.062	0.101	230	tags=57%, list=28%, signal=75%
HALLMARK_Inflammatory Response	42	0.15	1.16	0.267	0.699	0.982	570	tags=83%, list=69%, signal=251%
HALLMARK_Estrogen Response Early	21	0.21	1.13	0.276	0.576	0.989	188	tags=43%, list=23%, signal=54%
HALLMARK_Estrogen Response Late	16	0.23	1.10	0.320	0.525	0.997	536	tags=88%, list=64%, signal=241%
HALLMARK_P53 Pathway	40	0.13	0.99	0.459	0.606	0.999	539	tags=78%, list=65%, signal=209%
HALLMARK_KRAS Signaling UP	29	0.14	0.91	0.554	0.640	1.000	488	tags=72%, list=59%, signal=169%
HALLMARK_Apical Junction	21	0.11	0.60	0.909	0.938	1.000	346	tags=52%, list=42%, signal=87%

Table. S2. related to Figure 3E. List of KEGG and HALLMARK gene sets determined in MetaCluster #A with respect to other cell conditions by Gene Set Enrichment Analysis (GSEA) of scRNA-Seq data. ES, enrichment score; NES, normalized enrichment score; NOM, nominal p-value; FDR, false discovery rate q-value; FWER, family-wise error rate p-value.

Table. S3.

MetaCluster #B vs. Others

Gene Set	Size	ES	NES	NOM. p-value	FDR q-value	FWER p-value	Rank at Max	Leading Edge
KEGG_WNT Signaling Pathway	36	0.17	1.97	0.004	0.135	0.230	1265	tags=78%, list=51%, signal=155%
KEGG_Splicesome	18	0.34	1.74	0.016	0.226	0.599	1240	tags=83%, list=50%, signal=164%
KEGG_Long Term Potentiation	18	0.33	1.70	0.031	0.188	0.675	855	tags=67%, list=34%, signal=101%
KEGG_Calcium Signaling Pathway	26	0.26	1.57	0.053	0.300	0.879	1085	tags=69%, list=43%, signal=121%
KEGG_MAPK Signaling Pathway	73	0.15	1.55	0.058	0.299	0.906	855	tags=49%, list=34%, signal=73%
KEGG_Hematopoietic Cell Lineage	24	0.25	1.45	0.085	0.412	0.971	225	tags=33%, list=9%, signal=36%
KEGG_VEGF Signaling Pathway	22	0.25	1.36	0.149	0.444	0.996	637	tags=50%, list=26%, signal=67%
HALLMARK_MYC Targets V1	49	0.23	1.88	0.008	0.183	0.189	1077	tags=65%, list=43%, signal=113%
HALLMARK_Protein Secretion	46	0.22	1.77	0.016	0.167	0.306	1149	tags=67%, list=46%, signal=123%
HALLMARK_mTORC1 Signaling	82	0.13	1.43	0.090	0.439	0.865	1320	tags=66%, list=53%, signal=135%
HALLMARK_Estrogen Response Late	38	0.20	1.41	0.102	0.371	0.874	236	tags=29%, list=9%, signal=31%
HALLMARK_Xenobiotic Metabolism	42	0.17	1.28	0.170	0.527	0.974	1719	tags=86%, list=69%, signal=271%

Table. S3. List of KEGG and HALLMARK gene sets determined in MetaCluster #B with respect to other cell conditions by Gene Set Enrichment Analysis (GSEA) of scRNA-Seq data. ES, enrichment score; NES, normalized enrichment score; NOM, nominal p-value; FDR, false discovery rate q-value; FWER, family-wise error rate p-value.

Table. S4.

MetaCluster #C vs. Others

Gene Set	Size	ES	NES	NOM. p-value	FDR q-value	FWER p-value	Rank at Max	Leading Edge
KEGG_Ribosome	70	0.31	2.94	0.000	0.000	0.000	334	tags=69%, list=40%, signal=105%
KEGG_P53 Signaling Pathway	7	0.54	1.80	0.016	0.150	0.570	263	tags=86%, list=32%, signal=124%
KEGG_Base Excision Repair	2	0.89	1.61	0.024	0.366	0.921	91	tags=100%, list=11%, signal=112%
KEGG_Spliceosome	5	0.56	1.56	0.063	0.346	0.970	372	tags=100%, list=45%, signal=180%
KEGG_Cell Cycle	13	0.27	1.15	0.281	1.000	1.000	354	tags=69%, list=43%, signal=119%
HALLMARK_E2F Targets	10	0.40	1.52	0.057	0.518	0.803	89	tags=50%, list=11%, signal=55%
HALLMARK_Glycolysis	22	0.27	1.51	0.075	0.368	0.819	572	tags=95%, list=69%, signal=297%
HALLMARK_UV Response UP	23	0.25	1.41	0.110	0.421	0.931	448	tags=78%, list=54%, signal=165%
HALLMARK_mTORC1 Signaling	35	0.20	1.39	0.101	0.361	0.938	553	tags=86%, list=66%, signal=245%

Table. S4. List of KEGG and HALLMARK gene sets determined in MetaCluster #C with respect to other cell conditions by Gene Set Enrichment Analysis (GSEA) of scRNA-Seq data. ES, enrichment score; NES, normalized enrichment score; NOM, nominal p-value; FDR, false discovery rate q-value; FWER, family-wise error rate p-value.

Table. S5.

MetaCluster #D vs. Others

Gene Set	Size	ES	NES	NOM. p-value	FDR q-value	FWER p-value	Rank at Max	Leading Edge
KEGG_Lysosome	56	0.34	2.92	0.000	0.000	0.000	688	tags=61%, list=28%, signal=82%
KEGG_Glycolysis Gluconeogenesis	18	0.37	1.88	0.006	0.112	0.347	618	tags=61%, list=25%, signal=81%
KEGG_Adipocytokine Signaling Pathway	22	0.30	1.73	0.032	0.153	0.627	846	tags=64%, list=34%, signal=95%
KEGG_Natural Killer Cell Mediated Cytotoxicity	45	0.21	1.71	0.028	0.139	0.662	975	tags=60%, list=39%, signal=97%
KEGG_RIG I Like Receptor Signaling Pathway	19	0.29	1.48	0.085	0.319	0.954	1391	tags=84%, list=56%, signal=189%
KEGG_NOD Like Receptor Signaling Pathway	23	0.24	1.35	0.142	0.430	0.992	279	tags=35%, list=11%, signal=39%
KEGG_Proteasome	17	0.26	1.28	0.153	0.500	0.998	1844	tags=100%, list=74%, signal=380%
HALLMARK_Interferon Alpha Response	63	0.29	2.75	0.002	0.000	0.001	1466	tags=87%, list=59%, signal=206%
HALLMARK_Interferon Gamma Response	132	0.20	2.68	0.000	0.000	0.002	1142	tags=65%, list=46%, signal=114%
HALLMARK_Glycolysis	49	0.32	2.60	0.000	0.001	0.004	948	tags=69%, list=38%, signal=110%
HALLMARK_KRAS Signaling UP	45	0.31	2.41	0.000	0.002	0.010	244	tags=40%, list=10%, signal=44%
HALLMARK_Hypoxia	80	0.21	2.18	0.000	0.004	0.029	948	tags=59%, list=38%, signal=92%
HALLMARK_TNFA Signaling via NFKB	131	0.16	2.05	0.006	0.010	0.072	864	tags=50%, list=35%, signal=72%
HALLMARK_P53 Pathway	72	0.19	1.84	0.010	0.028	0.223	370	tags=33%, list=15%, signal=38%
HALLMARK_IL6 JAK STAT3 Signaling	38	0.25	1.79	0.014	0.033	0.270	901	tags=61%, list=36%, signal=93%
HALLMARK_Oxidative Phosphorylation	58	0.20	1.78	0.014	0.034	0.296	1872	tags=95%, list=75%, signal=370%
HALLMARK_IL2 STAT5 Signaling	87	0.15	1.60	0.020	0.082	0.600	669	tags=41%, list=27%, signal=55%
HALLMARK_Estrogen Response Late	38	0.20	1.43	0.112	0.157	0.871	357	tags=34%, list=14%, signal=39%

Table. S5. List of KEGG and HALLMARK gene sets determined in MetaCluster #D with respect to other cell conditions by Gene Set Enrichment Analysis (GSEA) of scRNA-Seq data. ES, enrichment score; NES, normalized enrichment score; NOM, nominal p-value; FDR, false discovery rate q-value; FWER, family-wise error rate p-value.

Table. S6.

Laser	Detector	Fluorophore	Marker	Antibody Clone	Company	Catalog #
405	448/59	Brilliant Violet 421	CD163	GHI/61	Biolegend	333612
	525/50	Brilliant Violet 510	CXCR5	RF8B2	BD Bioscience	563105
	620/29	Brilliant Violet 610	CD127	A019D5	Biolegend	351326
	755 LP	Brilliant Violet 786	CD43	1G10	BD Bioscience	743615
488	513/26	GFP	GFP			
	664/22	Propidium Iodide	Live / Dead		eBioscience™	BMS500PI
	795/70	PE-Cyanine7	CD16	eBioCB16 (CB16)	eBioscience™	25-0168-42
561	579/16	PE	CD80	2D10	Biolegend	305207
	614/20	PE-eFluor 610	CD56	CMSSB	eBioscience™	61-0567-42
	692/75	PerCP/Cyanine5.5	CD33	WM53	Biolegend	303414
640	671/30	Alexa Fluor 647	RANK + Anti-Mouse/AF647	80704	R&D Systems + ThermoFisher	MAB683 + A-21235
	722/44	Alexa Fluor 700	CD45	HI30	ThermoFisher	MHCD4529
	795/70	APC/Cyanine7	CD14-biotin + SA-APC-Cy7	61D3	eBioscience™ + Biolegend	13-0149-82 + 405208

Table. S6. Related to Figures S9-10. Panel of cell surface markers and fluorophore-conjugated antibodies used in the multiparameter flow cytometry assessment of human CD14+CD16- monocytes, transduced with NOTCH1-ΔE lentiviruses or empty vector as control. APC, allophycocyanine; PE, phycoerythrin.

Table. S7.

Cell Type	Gating Strategy
Unstimulated Monocytes	CD45+ CD14+ CD16- RANK-
MetaCluster A (MC-#A)	CD45+ CD127high CD43+ CD56+ CD14+ CXCR5- CD33- RANK-
MetaCluster B (MC-#B)	CD45+ CD127dim CD43+ CD56+ CD14+ CXCR5- CD33- RANK-
MetaCluster C (MC-#C)	CD45+ CD127- CD43- CD56-CD14+ CXCR5+ CD33- RANK-
MetaCluster D (MC-#D)	CD45+ CD127- CD43- CD56-CD14+ CXCR5- CD33+ RANK+
M1 Macrophages	CD45+ CD14+ CD16+ CD80+ CD163-
M2 Macrophages	CD45+ CD14+ CD16+ CD80- CD163+

Table. S7. Related to Figures S9-10. Gating strategy for the identification of different cell subpopulations with the reported panel (Table S6).

Fluorescence Minus One (FMO) controls were used to set up all gates. Singlets were initially discriminated on SSC-H and SSC-A, followed by the exclusion of non-viable cells with Live/Dead fluorescent DNA dye. Unstimulated Monocytes were identified as CD45+ CD14+ CD16- RANK-. Cells from MetaCluster #A were recognized as CD45+ CD127high CD43+ CD56+ CD14+ CXCR5- CD33- RANK-; cells from MetaCluster #B were CD45+ CD127dim CD43+ CD56+ CD14+ CXCR5- CD33- RANK-; cells from MetaCluster #C were CD45+ CD127- CD43- CD56- CD14+ CXCR5+ CD33- RANK- and cells from MetaCluster #D were CD45+ CD127- CD43- CD56- CD14+ CXCR5- CD33+ RANK+. M1 and M2 Monocytes were identified as CD45+ CD14+ CD16+ CD80+CD163- and CD45+ CD14+ CD16+ CD80-CD163+, respectively.

Supplementary Table S8-in Excel sheet

Table. S8. Related to Figure 3A. List of genes differentially expressed in each MetaCluster (MC) as identified by the Leiden clustering algorithm.

Table. S8. Related to Figure 3A. List of genes differentially expressed in each MetaCluster

Gene	Cluster A	Cluster B	Cluster C	Cluster D
RPS14	787,023	549,8	450,345	561,324
RPS28	782,061	493,008	463,026	641,82
RPL30	767,531	518,478	452,069	483,514
RPS12	761,795	522,196	468,578	342,55
RPS27A	750,8	551,329	569,147	405,514
RPLP2	741,48	490,722	570,905	423,333
PABPC1	725,541	465,375	441,664	869,514
RPLP1	662,38	555,873	650,103	550,667
NAP1L1	662,161	536,69	670,724	459,937
RPL13	659,488	480,182	441,25	409,82
RPL38	609,383	434,627	352,664	568,712
RPL11	603,546	416,561	386,155	483,523
IL7R	592,418	562,082	8,18103	15,4685
RPS21	576,145	388,398	403,362	289,234
RPL14	560,682	465,567	402,31	318,775
RPS3A	533,061	381,831	376,181	310,468
RPL23	517,464	395,245	464,095	509,36
RPL12	500,708	371,002	382,603	343,009
RPS23	500,485	332,859	391,069	340,811
RPS19	494,239	483,986	531,017	290,955
RPL27A	491,645	393,833	317,819	326,09
RPL5	484,73	353,027	386,716	262,063
RPL3	430,507	284,32	281,052	216,288
RPL29	421,987	336,022	351,828	411,36
RPS2	391,124	303,922	384,216	262,505
RPS25	381,79	268,467	251,845	224,153
RPL19	380,215	255,118	291,069	284,045
RPS15A	379,284	271,543	187,345	189,793
RPL35A	345,047	284,531	233,897	312,82
RPL27	344,724	260,818	260,664	264,414
RPL10	343,861	266,559	243,25	310,721
RPL36	319,215	245,808	187,672	273,171
RPS3	318,557	258,18	188,552	189,378
RPL4	312,69	229,082	257,06	153,793
SARAF	309,95	180,71	135,629	275,523
RPL23A	301,717	251,565	250,983	191,225
ETS1	300,289	292,396	138,043	3,45045
RPL10A	291,283	171,782	215,026	147,009
NPM1	287,313	183,184	216,966	138,009
RPS26	257,638	212,147	161,371	144,279
RPL7	250,632	193,335	190,543	204,432

EEF1B2	239,632	160,424	203,328	101,045
RPS5	231,847	140,622	215,009	120,793
RPL22	224,092	136,522	157,509	145,477
RPL18A	213,729	153,443	218,095	167,189
ANP32B	204,9	159,786	121,267	161,396
DDX17	200,898	116,518	185,5	214,279
PTPRC	196,124	241,014	95,7414	118,775
RPL18	193,635	146,341	150,259	128,414
SPOCK2	174,79	119,833	60,8362	0,405405
RPLP0	168,029	147,937	149,302	74,2342
TRBC2	167,291	140,473	41,3103	0,477477
PPP2R5C	166,945	240,902	73,9741	107,532
RPSA	158,352	125,604	109,353	45,036
RPS15	130,359	98,4449	90,4655	108,838
NDFIP1	123,952	39,7327	7,09483	67,6396
AES	120,271	78,9184	65,931	63,8018
LEF1	118,906	17,6388	0,0862069	0,171171
KIAA1551	111,279	87,9429	75,5086	24,3784
ARL4C	106,947	137,155	33,1293	25,8018
SELL	104,16	33,6102	9,77586	4,4955
KLF2	103,173	73,8	50,3621	38,2162
GCC2	97,6769	73,5714	54,9828	48,1171
CNOT6L	96,5767	174,757	39,7241	24,3243
RASA3	94,7819	50,3143	11,4138	27,2432
RAC2	94,5929	58,5122	79,75	60,4955
PIK3IP1	91,1922	49,4367	59,3276	19,973
GIMAP7	90,0081	36,4857	0,112069	7,72072
SLC38A1	89,7399	84	80,1207	5,91892
IKZF1	88,8772	55,2653	13,7241	10,6937
CELF2	88,6704	72,7673	30,7328	87,027
LEPROTL1	86,5832	52,0102	7,93103	45,7297
PSIP1	86,2052	54,2775	66,6207	10,5225
WASF2	83,6155	49,9633	42,3879	57,5225
CCR7	83,609	8,6898	75,3621	5,35135
C6orf48	83,2827	57,0041	55,319	39,3874
TRAC	83,0129	53,6469	1,10345	0,684685
TCF7	80,6187	17,6224	8,13793	0,864865
RCAN3	77,9758	31,6143	30,7586	10,8649
SAMHD1	77,9128	48,551	3,67241	128,523
SLFN5	77,0032	82,498	12,319	50,0811
ABLIM1	76,8659	64,0571	29,4914	0,162162
AKNA	76,063	92,0694	29,8103	21,8108
SATB1	75,9774	37,5531	25,4741	12,5766
SYNE2	74,4588	70,7163	24,8707	2,75676
BIRC3	73,0323	43,8673	68,4052	41,8829
FOXP1	72,8094	48,402	55,8707	25,6216

ITK	70,0307	28,5367	0,0948276	2,67568
CD3E	69,7286	60,8592	0,181034	0,504505
RBMS1	68,9919	32,6837	12,8879	42,4595
ATM	67,496	36,6061	34,8966	23,1712
RIPOR2	65,0905	20,4735	22,4914	34,8649
BCL11B	64,7286	31,9286	0,0862069	1,72072
PDE3B	62,8659	47,4755	19,8103	0,144144
CCND3	62,5945	35,8265	41,2845	28,3423
TSPYL2	61,8805	65,2449	52,1466	5,57658
CD2	61,021	64,6041	0,0517241	1,67568
CD48	60,2827	27,3469	29,2069	8,01802
SYTL3	58,1405	94,7878	11,2845	16,9099
ADD3	57,58	40,5388	34,2155	14,8468
RGS10	56,42	22,1755	9,96552	54,7928
EMB	56,3926	80,5775	50,7241	15,3063
TOB1	54,8045	63,2367	7,71552	8,90991
PIM2	54,6979	27,7837	68,7241	13,009
S1PR1	54,3473	41,2061	48,1897	0,288288
CD7	53,5654	70,5061	0,137931	10,9099
KLF3	51,4636	28,5184	21,2328	49,8018
DGKA	50,8013	17,6245	15,8362	10,2252
IPCEF1	49,895	26,3245	0,0689655	2,14414
FAM102A	49,1632	51,1592	14,3966	1,63964
CD96	49,0468	87,1041	4,63793	0,171171
TRBC1	49,0468	51,9286	0,12069	0,234234
RASGRP2	46,1922	15,8939	22,4397	3,6036
LBH	46,1422	30,0224	19,6897	0,765766
KIF2A	46,1131	31,4143	21,2414	10,0541
EVL	43,3603	32,0163	13,9655	1,55856
R3HDM4	43,3053	22,9653	32,681	45,1261
IL6ST	43,084	13,8816	10,1207	48,4144
IL32	42,0258	28,8776	0,0948276	12,7117
FCMR	41,9144	9,32653	27,6466	0,0810811
SMDT1	41,0145	17,0857	24,7155	39,8198
C12orf57	39,9418	15,7347	5,87069	3,40541
LIMD2	39,4814	24,149	35,6638	12,1081
CRYBG1	38,8805	47,2286	9,25	26,8288
CD3D	38,7577	44,598	0,0862069	0,18018
CD3G	37,546	27,6816	0,0344828	1,01802
CLEC2D	37,2973	42,6918	38,3448	3,16216
CD52	37,0872	17,4816	36,6983	7,48649
APBA2	37,0275	11,1612	0,0258621	0,0810811
RETREG1	36,7286	6,78367	3,51724	1,97297
ZNF101	36,0953	9,66939	8,09483	4,09009
CAMK4	35,8384	18,0408	0,387931	0,027027
BTG2	34,5977	12,5918	33,9397	44,2432

GPR171	34,3764	50,749	0,137931	5,54054
STX16	34,336	14,5571	22,1638	27,2342
TRAF3IP3	33,4766	15,2776	13,8103	1,96396
PITPNC1	33,2294	29,2857	2,2931	2,54054
TNFSF8	32,9321	5,97347	4,7069	0,027027
IKZF3	32,1809	26,2959	19,0259	0,045045
STK17A	31,7027	32,4796	31,7845	3,81982
ITM2A	31,0065	12,8184	3,03448	0,018018
RPS4Y1	30,8853	21,2224	26,8966	12,2703
NUCB2	30,559	6,60612	2,37931	3,0991
PRMT2	29,9338	13,5816	11,4483	15,3964
MYC	29,9047	4,21429	4,81897	0,306306
KIAA0355	29,8239	15,0388	10,9052	4,36036
FBXO32	29,2165	14,1551	2,16379	5,16216
LAT	29,0614	14,7245	0,0603448	8,35135
NLRP1	29,0291	14,1306	15,8793	14
AL365361.1	28,9305	9,08571	6,14655	2,71171
CYFIP2	28,2052	17,1694	24,0172	0,324324
TESPA1	28,1115	15,2898	0,0517241	0,108108
SNHG25	27,9758	13,9306	12,3966	25,5496
ITPKB	27,7447	21,5571	19,5431	3,53153
MAL	27,6365	3,22041	0,0172414	0,036036
GIMAP4	27,1842	12,1673	0,0344828	11,3333
LMO7	26,8352	6,37755	3,30172	3,45946
YPEL2	26,5654	10,4837	2,78448	27,9099
LDHB	26,0065	13,6204	4,40517	2,86486
MSL3	25,6559	12,5388	5,2931	29,0631
SLAMF1	25,3667	8,73061	5,93966	5,97297
CRTC3	24,5638	12,0224	8,11207	23,2162
AAK1	24,2763	15,5673	0,198276	8,25225
C16orf54	24,0953	24,1796	2,06897	19,4324
SESN1	23,6753	7,36939	23,3362	27,3514
RNF138	23,6123	8,40204	16,069	24,7117
RASGRP1	23,5687	52,2163	4,15517	1,71171
KLF7	23,0436	6,76531	30,4483	18,1441
LDLRAP1	22,8611	4,67551	0,163793	3,7027
SOCS3	22,8158	2,30816	0,655172	29,4865
EZH1	22,1502	10,1367	7,48276	10,8288
CDKN2D	21,5929	6,72449	4,18103	28,3604
TRABD2A	21,0921	4,65918	2,58621	0,036036
GIMAP1	20,4087	11,8714	0,241379	1,95495
OXNAD1	20,3037	11,3939	2,80172	2,36937
CD4	19,8885	9,1898	0,0603448	5,83784
CD6	19,7835	17,2735	0,0258621	0,018018
SAP25	19,6656	7,53265	4,56034	5,6036
SYNGAP1	18,6963	4,20204	3,97414	1,38739

CDKN1B	18,6139	23,6612	3,53448	7,40541
LCK	18,4184	10,398	0,0431034	0,126126
THEM4	18,0695	13,7551	2,4569	2,5045
SCML1	17,4911	4,8102	3,86207	11,4685
ACTN1	17,1874	0,487755	0,0344828	39,8288
NBEAL1	16,3457	9,7898	13,7241	15,2973
PLAC8	16,3279	14,1143	1,13793	0,864865
GATA3	16,3005	26,8224	0,0258621	0,0630631
LTB	15,6527	5,32041	3,13793	0,0540541
JAK3	15,5444	4,41837	25,0517	3,47748
RASA4	15,4637	3,25918	5,18966	0,432432
JADE2	15,4507	5,6551	12,6724	3,23423
ARHGAP45	14,546	7,26327	6,64655	6,32432
SPATA13	14,4733	11,8653	1,37931	3,74775
LINS1	14,3086	6,48163	4,62069	0,468468
SERINC5	14,2908	5,02245	3,42241	3,26126
SCML4	14,1551	4,02245	0,0431034	0,018018
RAPGEF6	13,5735	3,80816	3,15517	4,86486
ITGA6	13,4798	1,88367	0,267241	2,67568
SLC7A6	13,2859	4,3898	1,75	1,33333
TECPR1	12,7351	2,87143	3,18103	3,98198
TNFRSF25	12,3005	3,63878	0,0172414	0,468468
ITGB7	12,0339	4,44286	0,801724	6,71171
TMEM173	11,9063	7,78775	0,0603448	1,3964
APBB1	11,8691	4	1,7069	0,027027
TXK	11,6753	1,83878	3,69828	1,62162
TBC1D4	11,3796	3,33673	0,0172414	0,018018
TRIB2	11,3086	5,09388	3,37931	1,03604
TTC9	11,3069	2,35714	20,069	0,018018
GIMAP2	10,9402	3,85918	2,33621	8,40541
CSGALNACT1	10,7738	2,73061	1,25862	0,00900901
LZTS3	10,6801	1,68367	2,67241	0,027027
CERS6	10,6155	1,22245	0	16,4324
RNF144A	10,462	3,08163	1,44828	1,96396
GIMAP6	10,3263	7,49184	0,0172414	10,2252
STAT4	10,1616	19,1796	3,77586	0,0720721
GRK6	10,1002	5,58163	2,62931	4,59459
NELL2	9,91761	3,57143	0,00862069	0,00900901
CDC25B	9,88045	2,08367	1,88793	5,30631
MAP4K2	9,69305	3,9	7,33621	3,54955
STMN3	9,64459	4,81837	1,03448	0,045045
TSHZ2	9,39418	1,10816	2,42241	0,018018
TMEM204	9,13086	3,05918	0,0948276	0,027027
IL11RA	8,98869	1,59796	2,17241	0,0720721
CHMP7	8,87399	1,52041	6,41379	1,7027
GIMAP8	8,86107	2,2551	0,0344828	5,38739

CDK5R1	8,7496	2,74082	9,13793	0,027027
VIPR1	8,05654	1,1102	0	0,027027
AL136454.1	7,97738	7,09592	6,98276	3,95496
PLAG1	7,75767	2,67755	0,810345	0,630631
IGF1R	7,75606	1,75918	9,28448	7
LRRN3	7,63489	0,883673	0,0172414	0,018018
LINC00861	7,57027	0,236735	0	0,0630631
CD27	7,50565	2,3898	3,68966	0
RPS10	7,4685	3,00816	5,07759	6,23423
NOSIP	7,42973	4,87347	0,594828	3,92793
PRKCA	7,34572	1,55714	0,0258621	4,63063
KRT72	7,042	0,481633	0,00862069	0,018018
GIMAP5	7,02908	2,56122	0	0
TAF4B	6,82876	0,54898	11,6638	0,027027
IFITM1	6,77544	2,48367	1,75	5,86486
BEX3	6,75444	0,873469	0,0431034	1,77477
SLC7A8	6,71567	0,00612245	0,0689655	1,94595
GPRASP1	6,25848	1,0102	0,0258621	0
ZNF836	5,99354	2,3102	4,05172	4,17117
FAM153A	5,71082	0,410204	0,00862069	0,018018
FHIT	5,36834	0,373469	0	0,027027
RNF157	5,04847	0,863265	0	0,036036
FBLN7	4,79968	0,932653	0,0172414	0,027027
PTK2	4,72536	0,0693877	7,15517	1,23423
DHX33	3,1147	2,30408	0,224138	4,43243
TIPIN	2,31664	1,58571	2,91379	0,432432
MPZL3	1,4475	3,34694	0,0344828	0,045045
MTRNR2L10	0,306947	0,130612	0,12931	0,477477
RPS27	1614,3	1417,84	1179,43	968,469
RPL34	1557,02	1199,57	947,009	1095,59
RPS29	1409,46	1046,6	859,043	673,622
RPS6	1364,11	1033,27	998,621	653,865
RPS18	1110,31	861,822	803,422	653,748
RPS20	1045,01	853,08	828,716	748,658
RPL39	870,879	715,565	794,776	802,469
TXNIP	1076,91	709,573	290,922	418,811
RPL37	866,43	695,357	633,603	851,324
RPL31	848,989	671,341	491,397	636,793
RPL9	927,245	652,01	595,302	592,018
RPL32	950,11	647,441	596,621	629,261
RPL13A	763,128	647,147	519,491	397,811
RPL21	793,346	633,839	530,181	408,766
RPS13	1079,59	618,571	653,56	944,198
RPS8	837,328	570,09	689,422	574,964
GZMH	0,00807754	8,61837	0	0
S1PR5	0,00807754	6,87959	0	0,00900901

EOMES	0,00969305	8,24082	0,00862069	0,00900901
GFPT2	0,0145396	11,698	0,0344828	0
PRF1	0,0694669	15,6673	0,00862069	0,00900901
KLRD1	0,486268	59,551	0,0258621	0,0900901
GNLY	1,08239	109,004	0,146552	0,252252
CST7	0,946688	33,8245	0	0,036036
MYBL1	0,363489	13,4122	0,00862069	0,018018
FGFBP2	0,82391	27,8469	0,00862069	0
TRGC1	0,436187	10,5224	0,0258621	0,00900901
F2R	1,54766	29,0592	0	0,018018
TRGC2	1,35541	25,1245	0,00862069	0
KLRB1	1,03716	20,9755	0,112069	0
CCL5	5,88853	91,2571	0,0948276	1,11712
FAM129A	4,51373	123,322	1,56897	3,63964
TBX21	0,36672	15,0939	0,103448	0,891892
AUTS2	2,70598	35,1143	0,948276	0,0990991
GNAO1	1,45073	14,2592	0,0517241	0,0720721
SH2D2A	2,23586	17,698	0	0,0540541
SLA2	5,82714	34,6082	0,0431034	0,045045
SYTL2	5,0727	29,7184	0,0172414	0,018018
PYHIN1	1,35864	18,9633	2,39655	0,045045
C1orf21	0,735057	22,451	0,0258621	3,99099
SAMD3	2,05331	9,32653	0,0172414	0,018018
SLC7A5	2,58481	58,002	2,61207	7,93694
BATF	2,36672	12,2327	0,163793	0,522523
CD8A	10,8304	43,7959	0,0517241	0,0990991
PERP	0,693053	15,2612	0,0344828	3,18018
CTSW	1,71244	6,51633	0	0,045045
HSPA6	0,0242326	5,85102	0,0172414	1,54955
GALM	2,66882	12	0,206897	0,504505
PTPN22	4,06785	25,0939	1	2,44144
EPHA4	2,13086	7,15102	0,00862069	0,00900901
TGFBR3	5,54927	17,6571	0,00862069	0,774775
ITPRIPL1	0,982229	7,22245	0,905172	0,81982
JAML	5,64459	18,3816	0,0258621	1,34234
OSBPL7	3,65913	18,2327	2,12069	1,20721
IL2RB	5,27141	16,149	1,00862	0,018018
MXRA7	1,05008	11,5082	1,43966	2
FYN	55,895	182,186	1,90517	13,4234
IL18R1	2,14055	14,1776	0,577586	3,54054
HPGD	3,89984	16,6775	3,14655	0,405405
NKG7	0,0161551	25,3245	0,37931	11,018
RUNX2	3,63813	19,851	1,10345	4,34234
RORA	19,6882	53,9551	5,26724	0,117117
CXCR3	1,77544	8,55714	2,24138	0
ERN1	4,15832	29,4755	2,01724	9,31532

MAF	4,94184	37,2918	0,0258621	15,8468
PARP8	20,1939	62,6367	7,11207	13,0541
CLSTN3	2,83845	10,5878	3,05172	1,32432
RNF166	4,6252	12,3673	0,439655	3,57658
DUSP16	14,5606	43,4694	0,0172414	18,0631
PLEKHF1	1,63328	10,5939	4,87931	1,57658
ATP2B4	9,83845	22,1224	3,32759	4,23423
DUSP2	8,37803	18,3735	6,38793	0,477477
TSEN15	3,97254	9,99184	3,24138	1,21622
PDE4D	30,6656	60,3388	6,9569	15,8559
CCL4	0,260097	26,8082	0	23,6396
PIK3R1	38,8481	109,159	26,1897	32,3243
GK5	4,99031	12,9347	2,85345	3,9009
YPEL1	1,10824	8,39184	3,08621	3,63063
TBCD	6,16317	12,3367	4,16379	1,94595
MS4A1	0,0565428	0,00612245	132,716	0,018018
IGKC	0,0484653	0,0142857	76,7241	0,018018
CD79A	0,20517	0,114286	292,06	0,0900901
IGHD	0,0726979	0,022449	81,2931	0,027027
IGHA1	0,0129241	0,00612245	24,7931	0,027027
IGHM	0,751212	0,0673469	335,922	0,0720721
LINC00926	0,180937	0,00204082	36,431	0,018018
PAX5	0,088853	0,0183673	23,9397	0,027027
TNFRSF13C	1,65751	0,891837	74,181	0,0720721
SEMA7A	0,166397	1,43673	30,0948	0,0630631
BCL11A	0,345719	0,887755	41,7241	1,90991
TCF4	0,0371567	0,363265	73,0345	5,20721
GNG7	1,20517	0,808163	24,431	0,135135
P2RX5	1,10501	1,55918	24,9655	0,027027
NR4A1	0,0452342	1,77347	18,9914	0,738739
PAWR	0,476575	1,75306	14,8621	0,027027
CDCA7L	2,79806	0,0367347	14,7414	0,00900901
SPPL2B	2,19709	1	19,7328	0,756757
CMSS1	1,5525	2,97959	19,1466	0
RAB30	5,30856	4,56327	41,3879	0,018018
FAM43A	0,962843	6,64286	37,1552	1,46847
KCNC3	0,119548	0,0244898	8,22414	2,22523
TSPAN33	1,48142	1,57347	24,6552	4,84685
IRF4	8,26817	5,56326	63,6121	6,94595
INPP5A	1,84491	0,108163	9,85345	1,27027
HIP1R	3,27141	1,35714	13,8621	0,0540541
TLE1	0,00646204	5,71633	22,9914	3,00901
IFT57	5,11147	3,19592	31,5862	3,8018
CXXC5	1,41195	4,0102	44,6983	12,3964
STRBP	6,78998	6,32653	32,6638	0,0990991
BCAS4	4,91276	0,857143	16,3793	1,27027

CD83	2,38288	1,12245	174,405	71,4595
TENT5C	4,03231	8,46122	27,6552	0,0990991
LINC00426	1,23586	4,64694	12,8448	0,00900901
TP53INP1	3,54927	6,63061	43,4914	9,86487
C7orf50	3,06624	3,93265	28,4397	6,14414
CD40	0,447496	0,581633	26,2069	11,8649
BCL2	24,6737	10,6776	77,6638	3,53153
CHPT1	3,17771	5,43878	39,9655	12,4685
PLCG2	0,00969305	0,991837	19,931	9,63063
LPIN1	11,0226	11,5898	49,7672	4,27928
CHD7	5,51535	2,7102	15,5603	0,189189
MIDN	6,7609	11,8857	61,2414	15,4144
MGAT5	4,43942	5,34286	19,5172	1,08108
MCM5	3,31664	2,24898	28,569	10,4685
YBX3	5,10339	7,57551	133,888	64,1892
INPP5D	13,2132	20,749	97,25	22,7568
POU2F2	13,8675	6,24082	74,7328	23,5315
ZNF844	3,92892	1,11837	10,319	1,0991
LINC01215	3,68821	1,49388	14,1724	3,54054
HHEX	0,00484653	2,90612	23,5	11,9369
GRASP	0,373183	1,62653	20,569	11,0631
PPP1R16B	2,76737	11,1184	22,0172	0,279279
RALGPS2	9,2294	2,25918	20,4397	1,86486
SHMT2	2,5315	0,881633	16,2586	7,27027
SP140	4,96931	9,48775	33,3707	7,63063
ORAI2	3,66882	2,0102	87,431	52,2072
BCL2L11	30,7076	33,0041	134,647	26,6486
RBM38	9,35703	9,40612	29,8879	1,52252
HVCN1	1,70759	0,757143	21,1552	11,955
RCSD1	25,6139	29,6918	100,034	17,2072
EZR	36,8788	64,6735	222,681	66,2072
ODC1	4,06947	10,902	22,1724	2,05405
HMGCS1	3,52181	3,1551	19,3534	8,26126
MUM1	5,12116	5,28163	19	4,41441
PARVB	0,0339257	1,23878	21,8966	16,0721
FAM53B	6,70113	10,8347	29,0948	5,52252
RHBDD1	3,15509	6,06939	17,6638	5
SLCO4A1	1,70759	2,01633	17,7241	10,6216
REL	14,2197	18,9694	123,138	67,5856
JUN	23,7787	49,9571	112,026	22,6216
ZNF318	5,43942	5,29184	24,4741	10,7387
WEE1	2,937	7,52245	62,1121	44,2523
FAM49A	0,0290792	2,90408	23,25	17,5946
VPS37B	1,59774	5,6	19,2931	10,027
SWAP70	0,693053	1,11633	51,3879	44,2793
RGS2	7,74475	43,7388	118,724	56,1982

UVRAG	5,92569	4,90408	24,2414	11,3694
UCP2	23,3974	12,3898	55,3362	15,9369
IL4R	6,45719	6,06735	26,6207	12,4685
MKNK2	22,1922	17,9735	75,1379	31,009
TCF3	7,85945	7,09592	22,1552	6,03604
ARID5B	11,1842	26,1163	44,9138	5,31532
ARHGEF12	0,470113	5,06735	8,12931	2,1982
CD37	44,8772	27,7286	140,793	64,4144
SMC6	15,9031	13,251	52,7672	22,2793
TLR4	0,00323102	0,00816326	0,00862069	134,658
DPYSL3	0,00484653	0,00816326	0	75,7297
CD86	0,00807754	0,00612245	0,00862069	131,405
OLR1	0,0355412	0,0346939	0,00862069	405,162
CCR1	0,00969305	0,00408163	0,00862069	110,441
IL1RN	0,00484653	0,0102041	0	72,1261
HCK	0,00484653	0,00204082	0,00862069	64,036
AQP9	0,0129241	0,0142857	0	101,423
FPR1	0,0226171	0,0204082	0,0344828	277,45
VCAN	0,0226171	0,0244898	0,0258621	254,784
TREM1	0,00484653	0,0102041	0	50,6847
TMEM51	0,00646204	0,00816326	0	47,3694
CLEC7A	0,0161551	0,0142857	0,00862069	118,252
SERPINA1	0,0145396	0,0122449	0	80,8198
MMP2-AS1	0,0113086	0,0122449	0	67,3694
LDLRAD3	0,0129241	0,00612245	0	51,4865
MCEMP1	0,00323102	0,00408163	0,00862069	42,2703
RBM47	0,0177706	0,00408163	0	56,8108
AC245128.3	0,0210016	0,00816326	0,00862069	96,2072
RAI14	0,0226171	0,00204082	0,00862069	82,2613
CD14	0,0161551	0,0326531	0,0258621	177,495
LILRB4	0,0323102	0,0142857	0,0172414	146,739
CLEC4E	0,0210016	0,0163265	0,0172414	124,784
APOBEC3A	0,0177706	0,0122449	0,0258621	117,036
RAB32	0,0258481	0	0	52,4054
RAB13	0,00646204	0,0122449	0	37,2432
MS4A7	0,0145396	0,0142857	0,0517241	150,243
ANPEP	0,0274637	0,00612245	0,0344828	117,586
S100A8	0,0145396	0,00612245	0,0258621	73,8198
TNS1	0,0323102	0,022449	0,0431034	153,09
SMIM25	0,00969305	0,0142857	0,00862069	47,6667
CXCL8	0,101777	0,357143	0,0862069	795,91
SERPING1	0,0193861	0,0204082	0,0258621	92,4414
CXCL10	0,148627	0,410204	0,232759	1078,03
SLC11A1	0,00969305	0,0163265	0,0258621	69,0991
PLXDC2	0,0193861	0,234694	0,0258621	359
CD163	0,0533118	0,0571429	0,0689655	217,55

TLR2	0,0274637	0,106122	0,0344828	194,874
DOCK4	0,00484653	0,0306122	0,00862069	49,009
TNS3	0,00807754	0,0142857	0,0172414	40,8468
CSF3R	0,0290792	0,0265306	0,00862069	63,973
TGFBI	0,00807754	0,00204082	0,00862069	17,8468
SEMA6B	0,0210016	0,0183673	0,0172414	53,5856
MAFB	0,0436187	0,279592	0,0344828	293,55
SLAMF8	0,0371567	0,00408163	0,0344828	56,5946
EREG	0,0452342	0,244898	0,0258621	229,811
DAPK1	0,00969305	0,0306122	0,00862069	30,6126
ALDH3B1	0,0565428	0,00612245	0	35,6036
CST3	0,206785	0,0346939	0,0603448	166,036
LYZ	0,350565	0,4	0,103448	462,036
LHFPL2	0,344103	0,0183673	0,0948276	243,351
GPNMB	0,247173	0,0183673	0	137,027
SORT1	0,0646204	0,00204082	0	33,4595
TNFAIP2	0,0306947	0,422449	0,0172414	226,189
SERPINE1	0,0646204	0,683673	0,0603448	344,027
CXCL3	0,365105	0,0183673	0,0172414	168,018
CD300E	0,174475	0,0285714	0,0258621	91,982
EPB41L3	0,248788	0,00204082	0	81,9459
C5AR1	0,932149	0,159184	0,0258621	361,892
SULF2	0,00969305	0,0714286	0,0172414	31,8378
C15orf48	0,0759289	0,702041	0,0344828	234,243
FGR	0,0242326	0,0367347	1,14655	313,847
SLC7A7	0,085622	0,0265306	0,0431034	39,8018
FCGR2A	0,0129241	0,306122	0,00862069	82,036
IGSF6	0,248788	0,00816326	0,00862069	63,991
CPM	0,324717	0,128571	0,00862069	103,027
THBS1	0,0371567	0,806122	0,0344828	188,856
TIMP1	0,463651	0,72449	0,508621	340,405
IL1B	0,00969305	0,0102041	0,62069	124,378
MYOF	0,00323102	0,259184	0,0172414	50,4054
FCER1G	0,741519	2,13469	0,112069	501,036
LRRC25	0,00323102	0,00408163	0,241379	38,3153
CDC42EP4	0,0113086	0,365306	0,00862069	57,2703
B3GNT5	0,0145396	1,07959	0,0172414	155,459
ZNF385A	0,235864	0,979592	0,87069	291,45
HAVCR2	0,668821	0,0857143	0,0344828	110,135
PTAFR	0,599354	0,638775	0,336207	204,297
LPCAT2	0,119548	0,591837	0,0258621	87,5856
CD68	0,287561	1,02449	0,0431034	146,207
TPST1	0,315024	0,0632653	0,0689655	48,1712
PLOD2	0,0355412	0,642857	0,0344828	75,8649
CXCL2	0,0210016	0,0326531	1,34483	142,063
MT1E	0,103393	0,863265	0,0258621	94,0631

SIRPA	0,00807754	0,15102	0,293103	42,5135
CXCL16	0,47496	1,09388	1,38793	276,216
C3AR1	0,0274637	0,734694	0	70,036
IER3	0,0145396	1,04082	0,0344828	98,3964
PLAUR	0,287561	0,857143	0,0431034	107,09
NOD2	0,203554	0,416327	0,0172414	53,8018
SLC31A2	0,95315	0,914286	0,586207	206,973
SHTN1	0,0743134	0,0142857	0,689655	65,5586
TFPI	0,00969305	1,17347	0	88,4955
SIGLEC10	0,0339257	0,477551	3,13793	255,117
NCF2	0,0145396	0,0408163	2,63793	184,82
SECTM1	0,434572	0,34898	0,0172414	51,955
MNDA	0,00161551	0,00612245	2,41379	135,784
STX11	2,80291	2,7551	0,0517241	306,441
LUCAT1	0,0226171	0,495918	0,0775862	30,7387
SLC25A37	8,80937	6,44286	11,7759	1316,58
TYROBP	0,248788	7,4449	0,715517	403,441
MAP3K20	0,261712	0,553061	0,0517241	38,2703
KCTD12	1,80775	0,787755	1,4569	176,568
SNTB1	0,830371	0,581633	0,103448	65,5946
SRC	0,630048	0,622449	0,00862069	52,982
EMP1	3,27141	1,54694	0,0258621	202,036
ALDH2	0,407108	0,108163	2,60345	122,216
SLC43A2	0,171244	0,0408163	6,13793	248,667
APOBR	0,0323102	0,922449	0,00862069	35,8288
BCAT1	0,268174	1,43265	2,7069	163,027
FBP1	1,84168	0,0265306	2,06034	145,135
PSTPIP2	1,49919	0,610204	0	77,7477
TFEC	0,0113086	0,00408163	3,9569	138,721
LRP1	0,109855	0,565306	0,922414	52,991
KYNU	0,0113086	0,00612245	3,43103	113,721
FAM129B	0,0468498	0,612245	0,586207	39,991
SOD2	22,5299	12,3388	15,6897	1610,12
IFIT2	1,3538	2,41429	7,57759	346,838
ST14	0,00807754	0,0122449	1,73276	52,6306
DMXL2	0,457189	0,767347	0,00862069	36,8288
KLF4	0,0242326	0,418367	17,6466	539,36
FLVCR2	0,453958	0,530612	0,465517	39,4144
ITGAX	0,0129241	0,220408	1,2931	41,2432
SAT1	13,9338	22,0878	50,1724	2302,61
GLUL	2,45396	6,96939	4,81034	372,649
DUSP6	0,612278	4,19388	8,75862	339,559
SNX10	2,87399	4,01429	20,7241	676,198
SPHK1	0,0145396	0,893878	0,474138	33,4775
FCGR3A	0,00323102	2,38571	0,00862069	57,3604
ACSL1	0,810985	0,814286	4,59483	145,856

PLEK	0,219709	7,59592	12,6379	473,694
CD300A	0,423263	2,63673	1,44828	104,333
VEGFA	1,25202	4,07755	9,06034	331,811
HMOX1	0,0145396	0,0102041	3,38793	77,036
GRN	4,23263	4,62653	13,5	504,297
MT2A	25,1793	21,5776	24,7069	1601,68
RAB20	1,4475	0,655102	0,0862069	48,4234
SPI1	0,164782	0,157143	7,01724	161,027
DPYD	4,4588	4,40612	0,00862069	194,342
SERINC2	0,292407	0,365306	1,92241	54,3964
ATF5	0,578352	0,146939	5,07759	121,126
LGALS3	1,46688	7,07551	11,9052	425,306
GK	1,47819	2,38367	0,0862069	81,3874
CTSL	7,17124	0,530612	0,0517241	158
DOK3	0,256866	0,726531	2,05172	60,2703
C1orf162	8,60097	2,92041	1,39655	254,784
OAS1	2,03554	1,6551	0,732759	85,8919
TIMP2	1,95638	0,414286	2,91379	102,342
PRRG4	0,00646204	0,0122449	5,18966	97,5045
ITPRIPL2	0,224556	0,520408	1,92241	48,8829
DOCK5	0,0193861	1,36735	0,0172414	25,7207
PYCARD	0,819063	0,440816	1,12931	43,7477
CTSB	15,6171	16,1306	21,8448	980,469
ALCAM	0,833603	1,76735	4,75862	132,757
PLXNB2	0,235864	0,17551	2,51724	52,4505
DSE	0,649435	1,59592	4,63793	122,676
CEBPD	0,0646204	20,798	0,0258621	362,324
RAB31	0,00646204	0,24898	10,2155	180,243
WARS	2,06624	0,891837	7,68103	180,892
LRRK2	0,00807754	0,0204082	5,25	89,0901
CAPG	1,00969	1,63673	7,26724	164,288
ENG	0,38126	0,906122	0,724138	32,1441
PLIN2	7,91599	19,6939	7,72414	559,874
AIF1	3,70275	0,212245	0	61,5766
IRAK3	0,0161551	0,516326	5,68103	96,5946
PILRA	0,295638	0,0795918	3,38793	57,6577
MXD1	3,88045	3,64898	6,09483	208,243
CORO1C	0,450727	0,918367	4,50862	88,6306
IFITM3	2,72698	4,17347	4,28448	167,775
AHR	8,1357	9,21224	3,93966	317,333
NCF1	1,85299	1,15306	23,8534	399,117
CCRL2	0,0161551	0,636735	1,94828	38,3964
IFI6	18,9628	14,4673	30,9828	913,982
ICAM1	0,862682	1,0102	7,76724	135,378
GBP1	16,4847	4,44694	18,8966	554,162
ALOX5	0,0258481	0,0387755	4,69828	66,1081

RSPH3	0,234249	1,57959	0	24,973
FNDC3B	2,23586	1,57755	0	51,2432
IFNGR2	4,01939	0,683673	20,4914	335,36
ADAM8	2,52181	5,7102	8,66379	223,486
ALOX5AP	9,35541	45,2265	33,8534	1165,03
RSAD2	7,37318	2,58163	1,59483	152,099
PDLIM7	1,31664	0,881633	1,2931	45,4324
CYBB	0,0355412	0,0306122	16,4483	213,973
LGALS1	0,938611	1,04286	6,23276	105,559
PLSCR1	5,10985	6,56122	9,63793	264,721
LGALS9	1,66397	0,6	6,78448	110,775
PHLDA1	0,495961	2,65102	0,00862069	38,5045
GAA	0,600969	1,28571	0,482759	28,8739
IFIT3	11,0775	3,02857	11,7414	312,063
LAP3	4,29725	6,36939	11,4914	260,459
ATP2B1-AS1	0,709208	2,00816	0,12931	32,6847
SCO2	2,49273	2,93469	10,2328	179,757
PSAP	35,1438	45,0816	78,7155	1820,32
GBP5	27,1389	5,56326	20,75	599,324
CREG1	6,46688	5,42449	7,25	212,721
PPIF	2,31987	3,07143	0,681035	67,1622
ACSL4	4,99838	10,4755	11,0172	291,162
NPC2	13,0372	10,851	27,4483	561,829
PECAM1	5,10016	1,14082	3,48276	106,333
FCGRT	0,995153	0,530612	2,06897	38,1712
MFGE8	2,8643	1,05918	0	41,6126
ARHGAP18	0,376414	1,20816	1,4569	32
CTSD	4,61389	7,17347	6,19828	188,847
TNFSF10	6,08562	4,22041	1,50862	123,604
OAS3	3,43619	1,86939	6,27586	121,162
P2RX4	1,04039	2,11837	0,0172414	33,0631
SGK1	3,79968	3,19796	9,90517	173,351
SPATS2L	1,00323	1,18367	0	22,036
NINJ1	2,46365	4,89184	9,36207	168,333
FTH1	158,981	381,088	254,819	7996,82
TYMP	0,242326	0,404082	8,49138	90,9099
LILRB1	0,00807754	5,2898	5,44828	106,829
ANXA1	34,6543	70,9612	1,68966	1062,06
MPP1	1,76898	1,98367	0,75	43,7838
DUSP1	6,38934	13,1592	17,8103	358,252
CD63	3,67851	8,51224	19,7414	296,243
CTSS	19,5994	12,4122	52,2414	773,405
AGRN	0,274637	0,273469	0,431034	8,92793
NLRP3	2,59774	3,78571	0	58,1261
METTL7A	0,177706	1,95714	9,46552	105,207
SCARB2	5,10339	3,53673	6,86207	139,874

TMEM170B	1,36349	1,87551	1,5431	43,0901
CYFIP1	0,663974	0,618367	1,36207	23,0901
GSN	0,235864	0,610204	1,62931	21,4595
ABHD12	1,66397	3,58163	6,16379	98,6306
ZMIZ1	1,13247	2,20204	7,19828	89,8829
SLC15A3	0,208401	0,22449	3,10345	30,1081
LAIR1	2,71082	3,90816	2,00862	73,2342
APLP2	7,51212	13,9347	13,1638	292,847
APOL1	2,8433	0,553061	1,86207	44,2252
CLMN	0,386107	0,0142857	2,78448	26,7658
CMPK2	1,47011	0,177551	0,198276	15,4775
ETS2	1,3441	3,03469	1,67241	50,6036
S100A11	32,5977	47,9469	2,5	693,279
CD109	0,617124	3,46327	0	33,7117
EMILIN2	0,0323102	0,985714	3,25862	34,5495
GRINA	6,81099	5,0551	16,25	227,063
ATF3	0,785137	4,25102	9,37069	115,82
SAP30	0,594507	6,65918	0,396552	60,8829
FOS	5,25363	15,4959	68,069	703,784
MT1X	7,00323	4,86122	4,74138	131,081
LYN	0,337641	4,90408	18,1293	181,775
SMIM3	7,57674	10,6796	6,0431	187,928
MID1IP1	4,84976	5,24082	1,37069	88,5315
HMGB3	0,368336	1,45714	0,681035	19,1802
NEDD9	2,04685	6	3,69828	89,3514
RNF19B	8,77383	11,8408	12,2069	247,91
PDE4A	0,122779	3,82041	0,146552	30,7297
CCL3	0,0145396	13,9327	1,36207	113,153
S100A10	41,6333	50,8857	35,1466	943,243
RHOQ	5,50404	8,79184	14,7672	212,847
HK2	7,88853	4,70408	14,9224	201,369
EPSTI1	6,0727	1,92857	14,6552	164,441
ARHGAP31	0,684976	0,677551	2,39655	27,2432
NAMPT	12,0323	15,3653	28,3621	400,081
OASL	0,840065	4,53061	0,0172414	38,6216
SDC4	0,292407	2,28571	0,00862069	18,4324
ANXA5	11,3441	24,7531	36,0086	512,541
QPCT	0,52504	0,204082	4,65517	38,0811
GNS	4,20678	3,7	2,86207	76,0541
NQO2	1,76252	1,27143	0,939655	27,9459
BRI3	24,8659	18,6571	26,6293	491,306
ANXA4	0,987076	2,92245	2,27586	43,2613
MT1F	1,97577	0,857143	0,284483	21,5946
CORO1B	1,56543	0,230612	1,30172	21,4505
FURIN	5,96931	13,1673	10,4914	203,955
PHC2	5,15347	7,07551	8,4569	142,054

ANXA2	5,10339	18,7571	17,9914	285,189
LDLR	2,75767	1,74082	0	30,3694
AK4	0,765751	1,95102	2,60345	35,8018
SH3BP2	2,12116	2,20204	2,68966	47,1171
VIM	266,68	204,108	239,862	4755,61
MYO1G	0,568659	0,738775	0,275862	10,5315
RCBTB2	2,21163	2,3551	0,301724	31,9009
ADAM17	8,16801	13,5163	13,431	226,559
CPPED1	0,526656	2,01224	1,71552	27,3514
TSC22D1	1,60097	4,3898	4,27586	65,7928
IRAK1	4,80129	5,26327	7,75	113,667
FAM214B	1,26656	2,06122	0,706897	25,7297
PTGER2	0,959612	9,65918	0	67,6126
SLC43A3	0,621971	1,27755	5,68966	47,8559
LIMS1	11,895	10,1796	11,4741	210
PIK3AP1	0,0145396	2,87959	19,2414	138,108
PLIN3	2,21648	2,68367	5,37069	63,9459
AC020916.1	0,588045	2,43673	5,31897	51,7928
ATP6V1B2	5,0517	12,7245	8,93966	164,658
SLC31A1	1,32472	1,19388	4,5	43,2252
NFKBIZ	13,7108	7,15918	10,181	190,108
HEXB	4,87884	7,09796	1,74138	83,009
OTUD1	3,56058	4,74898	4,67241	78,018
ISG15	6,35864	8,41429	10,7759	153,126
IL13RA1	0,00969305	0,0122449	31,5776	187,189
MSRB1	0,0226171	0,155102	2,42241	15,1802
SH3BP5	3,82552	4,97551	6,5	88,1712
RXRA	3,21486	5,28367	3,43966	68,4865
RNF144B	0,426494	0,0244898	6,03448	37,1171
CPD	10,6026	24,2286	9,73276	254,829
ITGA5	8,14701	9,77347	0,12069	102,802
NDRG1	4,43457	10,8918	8,78448	137,081
UNC93B1	0,463651	0,489796	2,75862	21
JDP2	0,00969305	0,00612245	3,81897	21,5045
PLXNC1	1,04362	2,25918	4,77586	45,1892
FTL	65,21	45,502	67,5776	980,279
SDCBP	19,5606	22,0388	19,069	333,459
MARCKS	0,0226171	0,0142857	25,3879	139,009
CASP1	3,60582	5,25714	4,49138	72,3333
QSOX1	2,11309	2,19592	6,72414	59,7207
RIPK2	8,23909	7,18571	13,7759	156,604
MYO1F	0,92084	7,45102	1,57759	52,9099
CTSO	2,62197	1,70816	2,67241	37,0991
AGTRAP	1,34733	0,577551	2,18103	21,7477
CEBPB	9,27302	20,7612	6,59483	192,856
TOM1	1,78352	2,07143	2,28448	32,2342

SLC16A3	2,37157	6,52245	17,75	138,928
TRIB1	0,0258481	1,27143	8,78448	52,2252
LACTB	5,2811	8,25918	23,5603	190,414
RNASET2	22,7754	15,4449	86,5172	639,054
MFSD1	6,45073	6,02245	7,28448	101
ALAS1	3,02423	1,88776	2,94828	40,1532
C3orf58	2,8853	3,17551	4,60345	54,0721
TNFRSF1A	3,0517	4,2551	0	36,973
SQOR	1,3441	2,56122	7,06034	54,9099
ABHD5	2,23263	2,35714	3,41379	39,8468
MR1	3,61551	3,24286	1,08621	39,3784
ACVR1B	4,16317	2,23061	0,431034	33,6126
DNASE2	1,56381	2,21837	3,94828	38,018
AZI2	5,94346	7,84898	0,336207	68,6937
LAT2	0,0678514	2,34286	5,56034	38,7387
GBE1	1,14055	3,61429	7,2931	58,3514
PLEKHO2	0,639741	1,01837	1,56897	15,5496
NCOR2	10,4556	12,5082	14,9569	181,973
MIR22HG	0,0759289	0,759184	6,23276	33,5405
S100A6	25,8821	27,6163	9,48276	298,405
PDLIM5	4,77868	7,67143	3,81034	75,9369
CKAP4	5,70275	2,52449	8,7069	78,4324
CD81	11,6494	20,1857	32,5	297,568
NCEH1	0,610662	3,77959	1,61207	27,6847
ZFHX3	0,801292	0,379592	4,0431	23,9189
ATP6V1C1	3,97738	4,51429	1,25	44,4324
FLNA	6,31825	14,098	3,09483	106,847
FRAT2	2,31502	2,60612	0,37069	23,8739
SRGN	64,8756	259,953	55,5172	1715,21
ADA	1,5315	4,56939	4,37069	47,2072
FHL3	1,47173	2,59184	0,810345	21,9099
LEPROT	9,10662	9,17347	8,30172	119,432
RTN4	15,6624	15,5837	13,6638	200,405
RASGEF1B	4,12762	4,26735	50,75	263,766
GBP2	39,8481	17,6653	26,7328	375,378
RHBDF2	1,08724	4,20612	11,7069	74,7297
MTRNR2L3	0,242326	0,144898	0,241379	2,74775
MAP3K8	0,599354	2,26327	15,2672	78,7928
MYADM	50,8691	46,4939	82,7414	780,144
CTNNA1	0,848142	5,95714	6,87931	59,009
SYK	0,00161551	1,49796	29,1121	131,495
VASH1	1,0727	0,877551	2,68103	19,8919
MDM2	6,021	13,349	6,81897	112,18
VAMP5	4,99838	5,1551	3,89655	59,9369
RNF13	9,32633	8,08776	4,64655	94
ASAH1	10,5347	10,002	27,3103	203,559

CACNA1A	0,0694669	0,197959	0,439655	3
ITGAV	1,29402	6,19388	5,09483	53,3333
FYB1	103,355	45,6306	0,0517241	630,568
RAB3D	0,962843	0,697959	3,06034	19,9009
DACT3	0,0355412	0,0265306	0,0258621	0,369369
MEGF9	7,42973	5,65918	2	63,1441
PIK3R5	9,34572	7,90408	2,90517	84,2793
MX1	11,9984	6,34286	20,75	163
GNAQ	6,81422	6,72041	0,00862069	56,1802
LCP2	17,1761	23,6327	2,81034	179,225
EAF1	6,33926	13,5347	7,13793	110,054
ZEB2	0,34895	22,8653	20,5086	177,739
FNIP2	3,29402	2,21429	0	22,3423
ZFAND5	21,5428	21,4551	30,9224	298,649
TNFRSF1B	3,91599	18,0592	4,57759	107,144
GPCPD1	14,9273	18,6163	22,319	225,216
CLIC4	0,00969305	0,00408163	9,43966	37,955
ELF4	3,49596	3,83265	6,14655	53,6847
GBP4	19,3441	8,96122	69,3621	388,45
TCIRG1	2,57027	3,57347	6,32759	49,5856
ATP11A	1,18901	1,42041	3,12069	22,7477
ERGIC1	9,63974	14,7939	28,5	209,577
AGAP3	2,98384	1,63265	0,784483	21,2342
FOXO3	18,0468	15,149	20,4224	210,676
TMEM120A	2,10662	2,60612	2,85345	29,6126
GNB4	0,00484653	0,102041	16,8966	66,2523
JAK2	5,68498	3,12653	3,67241	48,5676
ZC3H3	5,20355	2,5898	8,18966	61,8739
IFNGR1	7,37157	9,1898	25,0948	160,423
ATP6AP1	9,03877	10,3918	12,1638	121,64
SAMSN1	7,27141	30,8735	15,6638	205,676
NUDT16	1,97254	3,06327	1,31897	24,2613
RAB11FIP1	3,19871	5,84694	19,431	108,333
FLNB	2,06139	1,39388	1,19828	17,6847
FCHO2	0,615509	3,32449	6,92241	41,0631
LMNA	1,59289	9,06122	23,6724	129,405
SLC30A1	1,70113	3,72857	3,38793	33,1171
MBOAT7	4,51212	8,89388	4,94828	68,9279
ADA2	4,72536	6,43469	1,81034	48,5856
CAMKK2	1,9063	1,51224	0,267241	13,8018
MFSD13A	0,360258	0,810204	2,09483	12,2162
LAMP2	9,60743	8,7102	13,5776	119,216
GLIPR2	2,17447	5,99796	1,2931	35,3243
NFIX	1,60582	1,11429	0,637931	12,5135
SLC44A1	5,69144	9,42245	6,17241	78,8198
NANS	1,45234	2,47959	2,9569	25,4865

MX2	9,42326	3,39388	14,2931	99,8288
ADRB2	0,00969305	3,94286	1,52586	20,1712
BST2	5,1567	9,5449	14,9483	108,189
RUNX1	8,82391	7,86122	3,42241	72,6396
MTSS1	0,34895	3,73673	13,3276	62,8559
MAPK6	3,063	9,18163	10,9138	82,6937
VMP1	13,5977	14,5959	6,82759	125,009
SLC16A10	7,72859	1,75918	0	33,6937
FLOT1	4,27464	4,62653	8,81034	62,8288
DENND5A	3,47011	2,08367	17,6552	81,7838
APOL6	32,3441	24,3551	36,3793	323,198
PIK3CB	5,85299	2,62449	3,86207	42,8468
ARL4A	11,6155	10,2306	21,8276	151,604
ATOX1	0,723748	1,56531	2,57759	16,7297
HLA-DRA	0,583199	0,732653	214,595	741,955
EHBP1L1	1,5832	1,43061	2,07759	17,4865
PLEC	14,9628	7	3,76724	88,2883
BID	1,64297	0,585714	3,21552	18,6306
HEBP1	2,75929	0,991837	0,491379	14,4414
PDP1	4,33764	3,6449	3,58621	39,1712
GSTO1	14,4233	18,6449	19,8879	177,063
GAPDH	59,8449	133,163	176,793	1235,05
SPTLC2	5,78675	6,6449	7,93966	67,5586
TANC2	3,08401	5,99796	3,2069	40,6577
MAFF	1,25363	8,46735	5,88793	51,4324
CCDC88A	4,17124	4,03265	9,34483	57,5405
ETV6	5,73506	4,14082	13,6034	76,973
CTSZ	0,277868	0,297959	14,8276	50,2703
OSGIN2	3,7706	3,26327	3,66379	34,8559
SLC6A6	6,45396	13,5306	18,1552	124,063
CLIC1	4,01454	7,75918	18,4397	97,7207
CRTC1	1,60258	0,283673	0,775862	8,58559
DNASE1L1	1,5525	1,80204	0,672414	12,964
BCL6	6,11147	6,42245	9,9569	72,2342
PTTG1IP	14,8239	10,8286	10,0345	114,351
STXBP2	4,97254	5,86122	3,62069	46,3063
STOM	4,16155	7,15102	4,36207	49,964
PSEN1	4,1454	5,91837	5,44828	49,3874
SERPINB1	27,3732	15,8163	15,8879	187,982
ITGB1	12,748	33,0265	29,8707	239,946
VSIR	11,0372	3,91429	6,16379	66,9099
MMP24OS	7,11309	5,7	5,73276	58,5766
FGD2	0,0161551	0,0183673	11,3707	35,8468
PKM	40,8401	38,9816	94,8966	548,955
CTSA	2,48627	2,65102	1,52586	20,7658
DNAJB12	1,8853	1,53878	0,689655	12,7387

TENT5A	1,42488	3,97551	9,37069	45,5135
ATP6V0B	11,0081	9,55102	15,4914	111,072
MTRNR2L11	0,0533118	0,044898	0,0775862	0,540541
CD151	3,75929	2,26939	5,75862	36,2162
STK24	25,6026	38,7633	19,7155	258,027
FRAT1	2,3231	3,89796	0,931035	21,9009
SRGAP2B	1,35541	1,77347	1,18966	13,1171
PSME2	5,09855	7,43469	14,2069	81,1622
CIITA	0,0161551	0,0122449	6,06897	18,3694
CHMP2A	4,57027	6,51837	2,75	41,6667
IFI35	1,49435	0,728571	3,25862	16,4324
IFI44L	12,5105	4,0449	31,7155	144,685
UBE2D1	7,61066	9,68775	8,06897	75,8739
COTL1	7,9063	8,95306	17,3103	102,162
ITGB2	5,87399	10,9714	0,181034	50,7568
MTHFD2	4,51212	2,93469	1,11207	25,4955
C2orf68	6,35218	4,29388	3,97414	43,4955
TPD52L2	3,05493	3,3449	3,58621	29,5045
BACH1	9,55412	6,70408	26,6121	126,225
APBB1IP	23,1745	17,7408	8,87931	145,964
SLC35F6	2,32795	1,6	0,0431034	11,6216
GLRX	7,46365	5,80408	3,9569	50,2613
IFI44	6,63328	6,08571	7,76724	59,5315
BCL3	11,6527	6,21633	7,68966	74,0991
AP2S1	2,99192	3,90204	2,72414	27,8739
H2AFY	17,6284	19,1633	20,4224	164,64
STK38L	3,3231	1,74286	6,86207	34,2883
PLBD2	1,77383	2,49388	2,16379	18,4595
RALA	25,6543	16,3306	11,319	152,928
CSGALNACT2	14,4313	17,0776	13,0603	127,144
RTL5	1,34249	4,65306	2,62069	24,5315
THEMIS2	6,75767	16,649	37,6552	173,685
OTULINL	4,72536	2,05306	0,00862069	19,1712
NAPA	15,9628	19,5245	17,3707	149,198
NUCB1	6,68336	3,28367	4,30172	40,2342
SPON2	2,01777	10,5673	0,603448	37,1351
IRF7	11,7868	8,7551	24,4224	126
VAMP3	7,80937	9,23878	6,11207	64,8468
CTSC	3,5315	9,3	3,62069	45,9279
NAGA	2,79645	1,77551	4,80172	26,027
SNX20	5,42649	4,95714	0,698276	30,6577
GPR108	5,89338	10,198	12,2931	78,4955
KAT8	2,87561	0,963265	1,56897	14,955
NADK	2,66882	2,89388	10,6207	44,7477
IRS2	26,2181	30,8837	56,9138	315,009
SGPL1	2,36187	3,01429	1,33621	18,5225

ID2	15,1405	24,451	5,7069	124,793
TBK1	2,87561	5,50408	5,83621	39,0991
MCOLN1	0,610662	1,29796	0,732759	7,26126
PLK3	10,0129	7,44286	5,18103	62,1441
VIM-AS1	1,70759	1,69592	1,97414	14,7387
LIMK2	2,63328	2,58775	2,66379	21,5496
NMI	18,336	11,8898	20,1121	137,126
ERO1A	19,664	20,7347	19,1983	161,559
MANBAL	1,19548	1,00816	0,00862069	5,99099
CD74	10,0824	15,1122	348,828	1010,5
PNKD	2,78837	3,03061	2,47414	22,4054
PTPN12	4,2084	8,7898	24,4828	101,198
TCF7L2	2,47819	2,58775	7,66379	34,2883
EMP3	23,8546	15,9429	23,7328	170,892
TAP1	48,7205	30,1633	76,6466	418,063
SRGAP2	1,80452	1,82041	6,13793	26,1532
GCA	1,71405	0,62449	9,58621	31,7207
PRKAG2-AS1	1,92892	1,47551	3,19828	17,5495
TUBA1C	2,72698	5,37755	5,49138	36,0631
CTSH	0,226171	2,02041	53,9224	148,874
LDHA	69,4943	110,553	119,853	793,261
UPP1	12,8643	14,7306	2,30172	78,9459
HAPLN3	5,25363	0,255102	3,0431	22,5766
ERCC1	1,65751	1,26122	2,57759	14,4865
NFIL3	0,827141	5,63673	0,362069	17,973
HLA-DRB1	0,289176	3,69388	70,3017	195,198
ZC3H12A	6,0937	10,798	9,27586	68,7477
STX12	7,89176	4,64082	3,94828	43,1441
RASSF2	2,4168	3,86531	17,3707	61,8559
RALB	3,44911	3,42857	7,69828	38,1171
TRIP10	0,513732	1,3449	2,06897	10,2703
KLHL8	2,9063	3,68571	3,4569	26,2613
OS9	16,3166	22,849	26,3017	170,991
TMBIM1	12,7674	10,4449	19,5345	111,414
KIAA0930	4,48788	3,92041	3,62069	31,3243
ATP6V1F	15,7415	18,1612	48,9828	215,495
ATG16L2	2,73344	2,12245	7,08621	31,009
LPCAT3	1,80452	1,38776	1,36207	11,8198
LAPTM4A	8,49919	8,96326	17	89,3243
GM2A	1,87884	1,46939	8,36207	30,3333
FEZ2	1,75121	2,32653	3,0431	18,4054
FAM110A	3,60258	4,69592	3,23276	29,7928
VPS54	2,34895	1,65714	2,43966	16,6486
PTPRJ	9,28918	13,298	1,62931	62,5225
ADGRE5	10,0792	12,4449	7,25	76,8468
LY6E	10,4669	7,67959	9,97414	72,3784

SLC26A6	0,420032	2,28571	2,08621	12,2973
PRKAG2	8,87076	8,98367	7,90517	65,964
HSD17B11	10,4184	11,3735	12,4483	87,6577
MVP	3,99838	1,92449	6,81897	32,5315
ST8SIA4	3,6979	5,75918	5,78448	38,8378
GNG5	11,1583	16,9102	17,9483	117,153
RP2	1,70759	1,25102	11,8276	37,5946
ERLIN2	2,99192	2,24694	0,939655	15,6757
STK3	0,998384	1,91837	2,7931	14,4775
SOAT1	7,82552	5,09796	6,77586	49,6757
TFE3	3,2504	3,0898	5,39655	29,5766
FAM102B	1,29079	4,00612	3,62069	22,4685
TUT7	13,5509	29,3449	13,7241	142,631
GCH1	2,08401	4,49184	5,11207	29,3784
MAN2A2	4,78191	5,42449	2,24138	31,2793
HPS5	2,2811	1,91837	4,96552	22,973
TOP1	17,4039	19,0878	25,0948	154,279
ANKRD28	4,59451	8,16531	1,68966	36,1892
OAS2	9,52342	9,99592	11,7672	78,2883
STAT2	14,0258	8,80408	37,181	150,153
ZFAND3	9,57027	10,4408	6,98276	67,3423
MCUB	34,769	22,9939	24,9138	205,396
YWHAG	8,53796	10,8898	17,3534	91,3604
MGAT1	17,5105	18,1735	14,7586	125,189
CNDP2	8,32472	7,02245	17,3362	81
NCOA4	26,5267	23,4082	19,0259	170,901
RIN3	3,62843	1,94694	2,64655	20,3514
DHRS3	4,79645	3,44082	0,0258621	20,4505
TPP1	33,1163	37,2571	37,9828	265,604
CLIP4	3,66721	3,99388	0,00862069	18,7207
SHKBP1	6,48304	5,34898	6,85345	45,6036
CTNND1	1,28433	1,32857	7,65517	24,9459
QKI	14,8061	13,8347	30,0948	142,468
CLEC2B	17,3409	37,8714	10,3534	158,847
B2M	1408,95	1292,62	1718,11	10692,1
LRRC59	3,55897	8,15918	3,78448	37,4685
SMIM13	2,78675	4,23673	1,86207	21,4595
RAB1A	14,5897	16,8429	16,319	115,324
HSBP1	4,2084	2,83265	7	33,8829
SVBP	3,00323	1,44286	1,05172	13,2613
RNF149	17,7044	19,0082	8,16379	108,216
KIAA0513	9,28594	15,151	12,9741	90,1622
SAMD9L	20,5945	6,92041	25,6638	127,982
PSMA4	10,4669	14,298	8,98276	81,018
POR	5,67044	3,66531	1,99138	27,1712
RELT	1,19063	3,26939	8,94828	32,1081

PELI1	9,47657	8,7898	7,77586	62,2162
LATS2	0,642973	3,94898	4,43103	21,4955
SRD5A1	0,996769	1,98571	5,7931	20,8739
IRF5	0,0161551	0,763265	10,069	25,7748
PLD3	5,79321	8,92653	3,06034	42,1712
CRYBG3	2,67851	2,08571	5,11207	23,3874
AGPAT1	2,00485	2,13265	1,77586	13,973
SLC11A2	8,37157	6,86939	4,7931	47,2973
PARP14	15,0872	11,5898	58,9914	202,126
FAS	2,92407	8,0898	5,67241	39,3514
PGK1	49,1486	63,2878	51,1983	385,901
MLKL	4,91922	3,7449	2,25	25,7027
CALCOCO2	20,0436	13,9327	26,6897	142,811
TOLLIP	3,85137	3,87959	2,46552	23,9369
NOTCH2	2,46042	2,62041	10,1983	35,8559
IDH1	2,82068	2,54898	0,293103	13,2613
SERPINB6	3,92407	2,91224	5,56034	28,9369
PAM	1,71567	6,1898	3,98276	27,6577
KDELR1	19,1195	21,8857	20,0603	142,054
STX3	2,44588	4,14082	3,38793	23,0901
SPNS1	1,70436	3,70816	2,47414	18,2162
PANX1	0,909531	2,49796	3,93966	16,9279
UBE2L6	10,916	5,57755	25,2931	96,036
HLA-DMA	0,353796	1,3898	11,5	30,4144
G6PD	1,71244	1,78776	1,96552	12,5225
MCL1	68,1147	90,449	79,5862	543,91
ZNF267	8,13086	16,6204	24,9052	113,351
TRAF3IP2	3,29725	2,6449	3,36207	21,0721
PTPRE	4,23748	7,35714	0,241379	26,7928
TBC1D14	3,88207	4,97347	2,63793	25,955
YWHAE	13,9354	18,0184	29,8362	139,288
TIFA	6,1357	6,34694	5,93103	41,3784
PICALM	8,87884	17,1592	7,69828	75,5856
HMGA1	20,8918	14,5327	36,1983	160,216
AC100810.1	1,83683	0,967347	2,69828	12,3063
PAK1	2,69467	1,33878	3,43966	16,6847
ANO6	5,43619	4,92857	5,59483	35,6126
ARL8B	15,5121	23,449	16,681	124,108
CNIH4	7,47173	8,73878	5,07759	47,4595
ADIPOR1	7,4685	13,0796	10,4828	69,1712
APOL3	8,41842	4,75102	3,63793	37,4414
NPTN	3,7706	3,96939	7,43103	33,7658
VRK2	2,49273	5,25918	7,44828	33,8108
HERC5	8,62197	12,3306	12	73,2613
TRMT6	7,96123	8,46326	6,75	51,4775
IRF8	0,358643	0,6	70,3707	158,18

PTK2B	34,3118	54,9612	25,7069	254,64
STARD3NL	2,56058	4,38367	3,48276	23,0901
ENO1	60,7496	78,798	100,44	531,207
TM9SF2	8,30048	7,43469	12,8276	62,9009
KAT5	0,898223	1,61224	2,24138	10,4505
CTBS	8,72375	6,65918	6,88793	48,9279
MYD88	3,38126	3,60612	9,25	35,6306
CARD16	2,36834	2,19184	1,38793	13,045
ATP6V0D1	4,98223	7,7551	20,9569	73,8739
SKAP2	1,08562	8,45306	34,5776	96,3784
RTN3	27,3845	38,3	26,5431	201,477
AMPD3	1,87399	1,22245	8,26724	24,8198
B4GALT5	1,98384	5,61224	6,36207	30,4054
SNX27	5,41034	6,09388	8	42,3964
REC8	3,89661	1,5102	4,37069	21,2342
CASP4	10,2536	15,8673	18,9828	97,9009
ZDHHC12	2,42973	1,48367	1,03448	10,7207
YIPF1	2,18901	4,23673	0,267241	14,4955
POMP	13,7641	23,6653	22,0086	128,685
CLN8	6,6042	2,96939	6,87931	35,6126
IRF1	107,901	32,7469	225,207	790,18
KLF10	6,22132	7,29388	40,2845	116,09
TMEM189	3,82714	3,06531	3,7931	23
EIF4A1	2,10985	2,65102	2,26724	15,0901
SFT2D1	4,02908	2,76939	8,53448	32,9189
FAM49B	14,7367	16,5816	34,75	141,162
RAC1	34,4814	37,749	43,1379	245,874
TET2	2,73183	5,23061	10,5517	39,3333
PITPNA	4,42811	10,4449	7,68966	47,7027
SPAG9	6,94184	10,8082	9,60345	57,8108
CC2D1A	1,57997	0,918367	0,810345	6,98198
ZDHHC20	13,6397	14,4041	8,56897	77,1802
ARPC5	62,0921	50,1735	56,7672	354,856
SLC2A3	17,6575	22,6	29,681	146,82
IPMK	4,60097	4,8	2,63793	25,2432
MAP1LC3B2	0,663974	1,73878	0,137931	5,32432
CASP7	2,96446	2,17143	7,59483	26,6757
SLC38A10	2,06624	2,37959	2,36207	14,2072
SLC25A24	3,53958	2,8102	7,75	29,3694
IL17RA	16,8934	9,15714	6,75	68,2432
CD58	2,56704	12,8	9,05172	50,8018
GORASP1	1,91438	1,21429	2,2069	11,0631
MTHFR	3,56058	2,86735	2,75	19,018
CAPN2	9,94346	14,3898	5	60,7748
MEF2A	6,79321	5,87347	14,9914	56,991
HIF1A	18,3958	14,4939	27,2931	123,937

NT5DC2	0,966074	1,82041	7,90517	21,973
XKR8	1,4475	3,47755	2,47414	15,1892
RPN1	4,13409	7,4	6,91379	37,8649
DUSP5	0,0161551	4,81837	17,6724	46,0811
CTDSP1	11,6317	8,32041	7,7931	56,7387
CKLF	1,89015	3,12653	1,89655	14,1351
YWHAH	3,64943	5,54898	6,69828	32,4865
UBXN2B	3,6559	4,57755	8,49138	34,1441
SQSTM1	17,3312	15,7469	37,3621	143,55
UBC	19,874	33,1	32,6466	174,441
WAS	22,2391	23,1816	27,25	147,955
RNF181	2,31987	1,98367	1,9569	12,7207
PML	2,90468	1,96735	10,6983	31,5405
ITM2B	88,9935	59,2388	41,4914	383,991
STX6	3,92407	3,9551	6,61207	29,2703
ZSCAN16-AS1	1,92569	2,70204	1,94828	13,2523
EFHD2	9,13247	32,4878	12,6724	109,297
MAP3K2	17,1519	24,8612	32,6207	150,18
LAMP1	16,9483	18,8367	16,8017	105,333
TOR2A	1,55412	2,72857	2,39655	13,3694
PARP9	31,5945	15,9204	41,1897	177,505
H3F3A	66,5444	68,1857	116,621	502,955
HLA-DMB	0,00484653	0,636735	23,431	48,018
PTPN6	7,95153	1,7551	6,90517	33,1351
STAT1	69,5089	15,2531	64,5948	297,658
PRNP	14,6042	24,402	14,9138	107,261
FOSL2	3,74798	32,5653	3,55172	79,1532
DAP	2,70921	4,47347	2,9569	20,1081
ITPRIP	6,9483	5,4	3,21552	30,8378
SESTD1	1,85137	2,58775	3,5	15,7207
HK1	11,7706	12,4755	6,62069	61,0541
CHP1	2,97577	6,41224	3,37069	25,2162
ATP1B3	12,3554	27,1694	24,3707	126,18
CDK16	0,949919	2,63061	2,23276	11,4775
PRPS2	1,95477	1,60612	0,818965	8,63063
VKORC1	4,06462	5,92653	0,551724	20,7568
ATP6V0E1	37,7464	44,5163	57,9397	275,712
CGAS	1,80291	4,92449	10,819	34,4595
GPR160	3,2601	1,34694	6,41379	21,6396
TRIM25	5,57674	3,60816	4,00862	25,8649
RDX	5,03231	11,902	11,5517	55,8378
ARPC1B	2,16155	2,08163	1,93103	12,0901
BCKDK	1,8126	3,28367	5,60345	20,9369
SPPL2A	3,92569	9,47347	17,2845	60,027
RNPEP	5,69951	4,50612	5,4569	30,5676
PREX1	9,01131	23,1633	17,4397	96,7117

TGFBR1	1,52181	3,06531	9,37931	27,1892
AP1S2	14,5363	14,6122	23,1552	101,73
TAGLN2	55,1179	56,5469	148,534	506,036
PDHX	2,03069	2,09184	1,93103	11,7658
FAM241A	2,49758	0,583673	4,5431	14,7928
TMEM38B	5,35541	4,62449	14,1466	46,7117
MTMR14	2,62359	2,50408	0,327586	10,5586
TRAFD1	5,15347	5,83265	6,56034	33,9369
BAZ2B	2,79645	3,54082	6,00862	23,7748
FKBP1A	8,22132	12,4673	13,5259	65,8649
CTNNB1	13,4879	15,6061	18,7845	92,0631
P4HA1	29,9305	34,6898	69,3362	257,27
TFG	6,1874	5,53469	3,81034	29,7387
PRDX3	7,84814	8,22653	10,1983	50,2793
RILPL2	6,7496	8,20816	14,0776	55,3874
ARRB2	48,4023	55,5429	11,6379	220,333
DDX60L	7,64459	8,33061	6,78448	43,3694
YTHDF3	5,43619	6,96326	4,63793	32,4595
TRPS1	2,95477	1,9898	2,77586	14,6937
BIN1	8,49758	7,4898	4,18966	38,3964
DPYSL2	4,28756	8,08367	5,10345	33,2252
IVNS1ABP	12,8142	14,2673	7,97414	66,6396
SKIV2L	2,74637	2,12857	3,77586	16,3964
HLA-DQA1	0,0339257	0,489796	31,25	60,0901
COMT	6,17932	4,94082	5,2069	30,7568
BSG	5,75444	12,6	7,62931	48,9369
IFNAR1	22,1696	20,2673	31,4397	139,135
SLC35B2	2,58158	4,0898	11,1121	33,4775
GDE1	5,49435	15,4388	6,44828	51,4144
HLA-DPB1	5,45396	20,7224	42,0431	128,027
ATP2C1	2,38288	3,57551	3,7931	18,2883
NCKAP5L	1,51373	0,64898	1,62069	7,09009
NUP62	6,39418	9,03061	9,56897	46,8288
NUMB	8,70921	8,19184	13,2672	56,4414
CYB5R4	3,63328	2,58571	7,80172	26,2252
KDM1B	2,57189	2,17551	3,38793	15,1802
RAP2B	13,9596	16,3796	7,39655	70,3333
CD53	65,7108	54,7592	50,7328	318,793
BRAP	2,99677	5,2551	2,87069	20,7027
DERL1	5,51212	9,3102	12,069	49,8378
TNFSF13B	10,4087	4,06531	1,06897	28,7928
AHNAK	20,7997	34,7796	56,5172	207,631
GSTP1	14,0162	17,8367	30,4914	115,477
DUSP3	1,86914	2,97143	5,98276	20,045
HADHB	7,11632	7,43878	6,42241	38,5495
CITED2	10,6284	13,8327	3,2069	50,7477

SLC1A5	0,990307	6,86327	1,30172	16,7838
HLA-C	356,039	514,639	463,052	2443,46
IDH3G	3,31987	2,16939	1,63793	13,036
LTA4H	12,1922	10,4245	27,431	91,4865
JUNB	38,1793	16,4469	32,9655	160,009
GAB2	0,957997	2,81429	6,86207	19,4144
MED8	7,42649	3,72857	5,61207	30,6036
APOL2	5,21325	4,77143	6,31897	29,7117
NRIP1	10,3199	10,5265	23,7586	81,036
ERP44	12,6995	15,8265	27,7414	101,955
PSMB9	9,81422	12,4388	30,3621	95,2973
TANK	6,91599	8,10204	8,74138	43,027
ATP6V1D	1,55735	0,918367	1,31897	6,85586
DRAP1	13	10,751	6,42241	54,4775
DNAJC3	4,69952	8,41837	11,9224	45,1982
RBCK1	14,5864	10,5837	11,7155	66,4414
BNIP3L	33,5234	28,249	65,8879	229,937
SCAMP4	1,28756	2,04286	3,60345	12,4595
HDLBP	13,084	20,3796	14,9569	86,8468
PGD	6,8336	5,2449	11,819	42,8108
FAM120AOS	13,1309	10,2143	7,27586	54,8468
DTX3L	16,2827	9,76531	20,2672	82,955
SCAMP1	4,68336	5,9102	3,69828	25,5946
IL6R	9,6462	1,87551	2,13793	24,3964
IKBKG	4,4378	7,40612	6,85345	33,3423
VPS26A	8,03554	9,47347	7,94828	45,3694
C4orf3	44,0937	36,402	39,8362	214,279
SH2B3	3,44911	3,12857	3,43966	17,8018
IFIH1	8,8336	6,96735	30,8276	82,8468
AL731577.1	0,0678514	0,112245	0,12931	0,54955
SLC17A5	2,59451	3,18367	2,75	15,1261
DARS-AS1	1,81099	1,21633	0,00862069	5,36937
FOXP4	1,80452	1,8551	7,77586	20,2162
ADAM28	0,0113086	0,397959	41,7672	74,4865
RAP1A	28,5299	37,3735	28,1207	165,784
SEC11A	17,5977	30,3204	27,4828	132,712
FUT4	3,24394	1,05102	2,87069	12,6036
CHMP4B	24,9047	23,5163	27,9224	133,955
PAPSS1	3,26171	5,14898	5,7931	24,9009
GNPDA1	8,22294	7,02449	0,655172	27,8468
TAP2	21,3344	14,0082	18,75	94,4865
DENND1B	1,5832	3,00816	4,30172	15,5315
MAP1S	1,15509	1,3551	1,11207	6,32432
PPP1R15A	42,1341	38,9694	71,7931	266,937
TALDO1	4,57351	3,84898	4,56897	22,6396
CCDC9	2,14216	3,32449	2,87069	14,5135

TMEM30A	13,3829	9,30408	12,4914	61,1712
RARRES3	9,79968	4,06939	1,49138	26,7027
RGCC	60,2585	44,2571	26,1897	227,108
XBP1	8,72536	13,2653	1,17241	40,2342
SPOPL	3,38772	11,0449	12,9052	47,4324
HLA-B	837,551	883,126	999,328	4707,48
RNF130	37,3247	28,6347	13,7414	137,811
ALG3	3,20032	4,56735	0,655172	14,5586
NCSTN	8,76575	8,21837	12,931	51,6126
PEA15	10,8465	15,7633	40,5862	115,928
SBNO2	4,81099	3,17755	4,55172	21,6216
PDIA3	24,4039	31,449	22,6293	134,874
TMSB10	107,745	59,4286	67,4828	403,243
IL15RA	1,49435	0,236735	1,4569	5,47748
ZCCHC14	4,44265	4,54898	2,68966	20,0541
RNF213	41,6074	29,5163	56,0776	218,315
TMEM205	2,09531	4,54898	4,25	18,6577
RAB5C	6,57351	9,88367	14,0086	52,1712
PPP2R3C	5,68498	6,25714	7	32,3874
BORCS7	4,81422	3,22857	2,64655	18,2703
BISPR	1,25687	2,18367	4,38793	13,3514
CHMP3	14,9645	11,7898	12,0517	66,1622
HLA-A	567,313	756,278	583,517	3250,97
NDUFS4	6,937	5,65306	7,56897	34,3333
TMEM127	5,45234	9,12653	11,6034	44,5405
MTRNR2L1	2,21002	2,26327	1,12069	9,51351
PPP2CB	2,65751	3,29592	3,26724	15,6486
NFKBIA	115,386	118,618	83,25	538,144
WBP1L	3,17124	3,16531	1,08621	12,5405
VASP	20,0582	14,8143	31,0345	111,342
UBE2R2	35,0307	36,4551	37,569	183,784
LAPTM5	99,5137	114,518	336,017	922,757
HLA-DPA1	2	13,4551	95,9828	186,946
MAPKAPK3	1,31502	3,3898	0,0862069	8,02703
PPP1R9B	2,55897	2,36327	3,5	14,0721
SPTSSA	2,57835	4,09796	2,12931	14,7117
C20orf194	2,69305	0,304082	6,28448	15,5045
HIST1H2AC	1,58966	3,68163	3,14655	14,0541
TAPBP	20,3425	14,7347	46,8879	136,604
ZSWIM6	4,40549	8,7449	6,94828	33,4955
CDKN1A	1,18255	2,44694	46,0086	82,7117
PSENEN	5,67044	5,66531	14,5948	43,1802
CCDC28A	4,43619	9,1898	4,69828	30,4955
HLA-DQB1	0,192246	1,48163	57,069	97,7207
CHMP5	7,45557	9,78163	9,56034	44,5315
TMEM219	10,3748	9,58775	7,50862	45,6126

HOTAIRM1	1,56866	5,39184	0,594828	12,5135
MED25	3,79483	3,80816	5,4569	21,6036
P4HB	7,6252	11,0306	7,19828	42,7207
EMC7	4,80452	8,80816	10,5086	39,8559
PRKCD	4,2504	2,48776	19,6293	43,5405
NEU1	3,61066	5,54082	7,37931	27,2883
ELK1	3,12278	1,15306	3,12931	12,1892
KDELR2	2,08239	4,36531	5,49138	19,6486
F11R	3,39257	4,9551	3,08621	18,8108
APP	11,0695	5,29592	10,5172	44,2072
GAB3	1,03877	5,76939	0,0517241	11,2793
LRPAP1	3,40549	4,54694	2,44828	17,0901
XAF1	65,6349	40,6551	120,716	372,991
VPS29	3,74636	3,19184	4,86207	19,3514
ATP6AP2	23,5444	22,3102	20,4224	108,649
GPI	29,1858	37,6429	42,25	178,225
SFXN3	6,19871	6,22857	2,11207	23,7477
ACTN4	16,1373	30,9204	23,75	115,64
RBPJ	14,0792	28,9082	31,7759	121,991
RIC1	2,97415	1,27143	10,4138	23,9189
TNIP1	4,42488	12,2102	9,69828	42,9369
PLEKHM2	5,50081	4,85714	9,77586	32,7297
POLR2J	7,41519	11,6776	6,34483	41,2432
HTATIP2	9,31987	9,01224	13,6724	51,8468
MFSD12	3,86753	1,92857	0,568965	10,2973
SEC24D	3,9483	5,06531	6,13793	24,5045
SIAH1	4,56866	2,02245	4,21552	17,4775
OAZ1	54,1292	79,0694	157,853	470,649
CALM2	48,7706	45,1143	37,6897	212,667
PGM2	3,59289	2,59592	1,76724	12,8378
HM13	5,74475	7,9102	7,25	33,6216
VAT1	3,66397	2,58163	5,48276	18,8559
CCDC117	2,54766	3,41837	5,84483	18,982
METRNL	1,7609	8,81224	2,34483	20,7568
PFN1	134,961	140,769	132,905	656,288
RAB7A	44,601	47,4	31,4224	198,162
RAB8A	9,21002	10,8551	19,4569	63,4324
SEMA4B	3,00808	4,62245	15,3103	36,7568
JMJD1C	15,3457	20,9673	27,3707	101,829
NKIRAS2	2,53635	2,6449	5,25	16,6757
DNAJA1	20,9192	26,0265	45,8534	147,982
SHC1	2,24556	4,4898	2,64655	14,955
SPART	10,79	5,11429	6,48276	35,6667
UBE2E1	2,32633	4,7449	6,75	22,009
MTRNR2L6	0,0969305	0,1	0,301724	0,792793
CLTA	13,3813	13,5367	10,5603	59,5676

RPS27L	14,0824	14,0612	13,5431	66,0811
BBIP1	3,38449	1,63878	2,65517	12,1712
RTF2	6,42973	5,33878	9,28448	33,2793
INSR	0,101777	0,0714286	18,6724	29,7748
GNPTG	5,57189	6,79592	5,50862	28,2252
SH3KBP1	25,0598	27,3633	26,3534	124,315
NR1H2	3,77221	2,09592	5,92241	18,6036
MYO9B	7,03069	9,53265	13,8017	47,9009
BASP1	0,20517	0,583673	117,284	186,234
SNF8	4,3958	3,41429	4,44828	19,3153
RAB5A	14,7722	14,002	17,3017	72,4955
DYNLT1	13,8546	11,351	11,3276	57,3964
NFE2L2	16,7561	18,6673	19,5259	86,2793
SMG9	2,4168	0,867347	4,76724	12,6396
UBQLN2	15,454	11,0449	8,0431	54,2162
INAFM1	3,05493	2,94286	4,52586	16,5135
ARPC3	26,7108	33,7224	50,4138	173,892
C15orf39	4,03069	4,32857	2,87931	17,6216
SSBP3	18,2859	14,6612	7,75862	63,8108
PNP	11,4701	15,7918	14,8966	65,955
PSMB8	11,1535	13,6082	12,7931	58,7117
GBP3	4,68498	3,91224	2,59483	17,4775
MTHFD1L	0,781906	2,66735	7,23276	16,6667
GOLGA7	16,6817	19,5939	10,819	73,2703
ACER3	1,47658	2,8	3,89655	12,7117
FOXN3	23,6785	20,9837	28,4138	113,514
SPG21	8,76414	10,7694	11,8793	48,7387
TMEM59	15,5557	19,5102	17,4741	81,4595
CD44	63,0743	69,4612	62,2414	301,622
CDK19	6,88207	5,49388	15,4828	43,0901
PFKFB3	11,3845	11,498	11,6379	53,3784
SDF2	2,84653	3,92449	3,97414	16,5946
CYB5R3	5,43619	9,14286	14,1121	44,2883
NSRP1	13,4717	14,9612	10,7931	60,4414
BMF	1,59612	0,283673	5,63793	11,5766
TXNRD1	6,13247	5,17347	6,64655	27,6126
ETV3	3,91599	7,83265	4,87931	25,5496
EIF1	214,291	207,118	327,466	1148,72
EIF4E2	7,12924	8,00408	7,88793	35,3063
EPB41L2	1,26333	1,82245	4,76724	12,009
TRIM69	4,81745	3,28367	8,68966	25,6667
FOSB	12,8756	15,5388	89,569	180,126
TPM4	7,48304	11,9327	17,069	55,4865
NUB1	28,2391	27,8469	35,7155	139,495
ASAP1	7,35864	10,9796	11,3017	45,027
PGRMC1	4,31341	4,3898	4,10345	19,4505

M6PR	25,2342	27,3388	25,5	118,514
CYBA	3,00969	4,82653	4,9569	19,4144
IER5	6,05008	9,68163	19,8621	53,9279
EIF2AK2	23,3021	17,3939	37,9741	119,063
ASPH	2,56543	0,842857	2,87931	9,4955
MGST3	7,12278	14,649	7,33621	43,8829
MYL6	21,7948	30,8878	54,6121	161,568
DNTTIP1	1,36026	5,10816	2,13793	12,955
GNB2	3,33118	4,32041	3,66379	17,018
LLPH	4,26333	4,52449	4,12069	19,4054
UQCRC1	3,7916	4,2449	1,47414	14,2883
ULK1	8,48142	9,00204	12,1983	44,5676
CSRP1	6,05977	8,01837	3,14655	25,8468
PCBP1	66,3005	72,7306	85,8103	337,207
NFIC	11,2359	9,4551	8,2069	43,3243
BCL10	10,7819	9,69184	11,3621	47,7027
GALNS	1,93215	2,25102	3,81034	11,964
C1orf43	11,3958	11,8224	19,5086	63,9459
SH3TC1	4,43296	3,20408	3,60345	16,8198
TRIM22	43,7027	17,0959	69,9914	195,459
CAST	61,2795	67,4816	73,569	301,721
SPTLC1	6,19225	4,69796	8,85345	29,3784
ARHGAP21	3,78191	6,51429	1,34483	17,3063
PPP4C	13,7221	14,5694	10,1293	57,1171
SLC26A11	4,79645	4,01429	1,85345	15,8018
BAZ1A	44,5412	21,8816	28	139,811
ATXN1	5,96446	14,9286	7,11207	41,4234
CNNM4	1,23586	1,19592	2,69828	7,58559
ATP2A2	10,4459	12,1224	6,61207	43,1261
SLC35E3	2,39257	6,54898	8,73276	26,0811
ACTR2	104,606	135,167	133,138	549,964
PATL1	15,4426	17,2755	17,3276	73,7928
EIF5AL1	0,0613893	0,414286	0,12931	0,891892
MIS18BP1	8,7189	19,049	24,8448	77,5045
MTRNR2L8	342,964	311,547	457,155	1637,59
OSTM1	8,93053	9,96531	6,92241	38,027
ITPRID2	6,19709	4,84694	11,3276	32,9369
USB1	6,06947	5,59796	3,55172	22,4054
RAB18	6,33603	8,6551	5,41379	30,036
ATF6	16,7544	11,8163	9,49138	56,027
NOP10	14,1276	15,1449	16,5345	67,3063
RHOG	23,769	29,1796	32,0776	124,883
PHACTR1	0,0193861	0,022449	38,6207	56,7568
MARCKSL1	9,61389	6,35102	17,8017	49,5676
NF1	5,79321	13,3571	3,12931	32,7027
SENP5	3,46042	3,24082	3,77586	15,3784

DVL3	4,43134	7,13878	7,2931	27,6486
HELZ2	3,77544	3,70408	8	22,6847
DYNC1LI1	7,7399	9,55714	8,85345	38,2703
PIM1	76,1082	32,3327	61,8621	248,982
ABHD2	6,59289	7,76122	8,92241	33,991
BCL2A1	0,481422	1,87143	19,8534	32,4234
AP3B1	6,57835	8,35306	10,1638	36,5946
PSME1	23,4362	16,2245	47,0948	125,766
MTRNR2L12	281,635	249,296	357,819	1287,78
TMEM167B	11,5153	23,3245	21,3448	81,3333
STAT3	18,7851	17,4816	23,069	85,8559
MBD2	23,3942	35,2694	62,5259	175,351
AGPAT3	9,20194	4,04286	8,12069	30,8198
AKR1A1	8,67205	8,24286	20,7155	54,2703
TPI1	69,8158	70,0551	117,164	370,477
CAPZA2	21,8223	19,6898	24,3276	94,8378
TRIO	0,103393	0,844898	16,2586	24,7838
REEP3	3,12601	3,27959	4,26724	15,3604
LRP10	21,5057	19,1	16,4397	82,0721
HLA-E	344,956	304,31	318,414	1390,93
LRRK1	0,0193861	0,0122449	9,64655	13,9099
ARRDC3	10,7512	8,26531	5,2931	34,9009
DIAPH2	3,02423	0,769388	11,5172	21,964
S100A4	6,80291	15,9653	10,3448	47,4234
WDR1	10,6769	8,11224	7,46552	37,5495
RIOK3	9,35218	15,602	24,3017	70,3604
NDUFB3	8,3231	9,21428	4,96552	32,1171
HLA-G	0,739903	0,822449	1,12069	3,82883
SLC4A2	8,50727	8,84286	19,1466	51,982
CDC42EP3	12,0194	30,2204	10,1897	74,5496
ZNFX1	8,64459	7,06939	9,75	36,1982
GYS1	4,6559	3,63265	6,60345	21,1441
MAP1LC3B	19,9063	45,3	42,2414	152,486
ZCCHC2	11,7754	7,21429	23,819	60,5135
BHLHE40	3,50242	38,0592	50,7328	130,414
RNF146	1,91922	2,82653	2,75862	10,6036
SERINC1	35,9305	37,7551	30,3017	146,784
ABR	1,43942	1,82041	4,19828	10,5225
CYB5A	4,84653	3,26122	5,93103	19,7838
IL27RA	5,98223	4,11837	5,72414	22,2793
GNAI2	41,6672	46,5408	36,5431	175,108
MTPN	28,0953	40,7816	43,3621	157,396
OGFRL1	7,19709	12,7265	33,2241	74,4234
SNX3	25,1179	24,8918	42,5431	129,532
ATP6V1A	3,84006	5,67551	6,49138	22,3964
TSPO	4,02746	4,29592	3,78448	16,9369

MRPL52	8,75444	7,86735	4,60345	29,6667
VAMP8	18,9321	26,5755	23,2931	96,0721
PTEN	34,9628	29,6653	32,1552	135,135
CARD19	7,25363	5,43265	3,0431	21,9279
KIAA2013	3,39741	7,95714	6,18103	24,4234
ILK	3,47496	5,50816	8,06897	23,7477
CPEB4	1,40388	3,86735	3,55172	12,2703
CIAO2A	5,58805	7,77959	11,181	34,1261
SMS	7,20517	5,06122	15,1638	38,1261
TRIOBP	3,02262	4,36735	10,2414	24,4505
IGF2R	5,84006	12,0429	7,56034	35,2793
CHST15	0,55412	0,00408163	15,5517	22,3333
SH3BP1	6,69628	4,5	1,53448	17,6486
SUSD6	7,14055	9,9	15,5259	45,0811
GUK1	7,03393	6,33469	6,81897	27,9369
CD46	16,4669	20,5429	22,7241	82,5856
TRAK1	1,05816	3,70612	4,93966	13,4054
MIR210HG	2,85137	1,4898	7,89655	16,8919
COLGALT1	8,98061	6,83061	10,1983	35,8468
PRDX5	8,62682	10,9592	10,9914	42,1171
RNH1	7,41034	8,00204	8,58621	33,0541
MPRIP	15,2342	11,9061	7,77586	48,0631
MYL12A	36,3603	27,5184	20,1897	115,676
MAP3K11	1,51212	1,00816	1,35345	5,32432
CUEDC2	5,42003	3,98571	2,28448	16,0631
LRRFIP1	30,0743	42,2531	52,8276	171,937
PSMB3	6,78191	10,5102	16,9569	47,027
GLB1	4,5315	7,02449	11,9224	32,1892
PLP2	84,5993	83,5449	135,5	416,27
XIST	5,68659	8,62857	2,90517	23,5856
CDK9	4,48627	3,64694	2,84483	15,027
ATG3	5,68013	8,5	7,44828	29,5676
PHF21A	6,25363	11,7959	19,0948	50,5946
EVI2B	49,9661	86,4143	35,5517	234,171
CHTF8	10,0824	7,38367	9,26724	36,3964
DAZAP2	48,1405	45,8918	59,3707	208,838
TUBGCP2	3,34733	4,47143	4,68103	17,009
CMTM6	20,3037	16,6878	59,4483	131,117
UBA1	16,4039	21,902	18,4138	77,0811
WASHC5	5,33441	6,36327	9,12069	28,2523
KLHL5	1,09047	0,857143	11,7845	18,6216
PSMB2	12,2552	13,8694	23,2672	66,8378
PARP12	3,75121	3,98367	4,65517	16,7568
WDR45B	7,1454	11,0082	14,0948	43,6126
TXNDC17	4,6042	4,29184	5,82759	19,9099
TMED5	11,8514	19,7592	14,0431	61,7297

EMC3	5,58805	10,3694	4,55172	27,7297
FGFRL1	2,05977	1,23878	4,05172	9,93694
AKAP13	42,9499	59,5755	56,8534	214,964
AURKAIP1	7,67044	9,61837	8,84483	35,1622
STMP1	13,3667	13,549	14,7414	56,036
DECR1	6,79645	8,42653	4,40517	26,3874
ARF1	47,21	58,2551	53,7414	213,874
ACVR1	1,36349	2,63469	4,34483	11,2072
MPV17	4,03877	2,97347	4,01724	14,7748
TXN	7,19225	15,8551	25,7931	65,3784
RRAGD	3,25363	5,99388	0,491379	13,027
ZBTB43	11,2811	20,249	13,9483	60,8108
BANF1	13,9079	13,7673	19,9224	63,6216
UCHL3	1,48788	3,58775	2,75	10,4595
REXO1	1,22132	2,3898	1,74138	7,15315
NRAS	5,7916	6,67959	13,2414	34,3333
CANT1	2,50081	3,90204	6,68103	17,4595
MED19	3,70598	3,77143	6,18103	18,2252
ERAP1	10,4249	5,57551	10,4741	35,3243
UBE2Z	16,3958	18,0184	23,7328	77,5045
MTMR3	8,17771	6,53673	9,10345	31,7117
SMAP1	11,1309	14,4082	9,5431	46,6487
LMBRD1	9,84006	5,7102	17,4741	43,8468
RNF145	8,77221	12,249	18,6466	52,4054
PSMD8	6,53473	10,0286	8,00862	32,4324
CAP1	42,3199	42,0163	44,4138	169,847
DDX58	7,13086	4,64082	7,80172	25,8198
MAP3K13	2,61712	1,66327	5,00862	12,2342
DDAH2	2,03393	2,88571	1,48276	8,42342
PNRC1	82,9111	69,8837	81	307,541
SPTBN1	11,147	10,4735	18,7672	52,973
ZNF217	7,56704	7,30408	5,07759	26,1532
SERPINB8	5,28918	9,11429	8,16379	29,5586
TET3	3,37157	3,50204	8,12931	19,6486
RTCA	5,937	7,05918	2,69828	20,5405
CD55	32,4766	14,6204	44,8793	120,36
TRAPPC1	9,34249	9,33469	14,0948	42,8829
CISD2	5,44588	6,32041	4,14655	20,8108
KANSL1L	3,96607	1,26122	5,87069	14,4955
PIGS	4,44588	8,6551	4,48276	22,9369
CPT1A	14,9047	12,4143	12,431	51,8108
SGMS1	2,45557	5,37347	4,77586	16,3964
PDCD6IP	17,1195	23,3735	22,2155	81,5315
PSMB7	5,04039	4,83061	3,93966	17,9459
HSPA5	29,9628	39,8939	25,2759	123,568
POLE4	5,12278	5,31633	6,36207	21,8018

MOB1A	44,189	52,9388	64,1983	209,324
KBTBD2	15,1567	11,4408	12,6379	50,8649
GABARAPL1	12,0501	56,3061	3,18103	92,7387
ACTR3	42,9111	53,4694	69,0345	214,054
WDR45	3,08562	3,23061	5,75862	15,6216
DUSP22	6,00808	5,55102	12,5172	31,0991
TMEM50A	26,4523	48,1939	32,5	138,387
DENND3	0,424879	1,38776	16,7241	23,9369
GSAP	2,46527	1,36531	14,5776	23,7658
HDGF	29,3166	36,0531	29,9914	123,054
PABPC4	40,1228	34,3163	49,5862	160,036
SDHD	4,59128	3,01837	4,93103	16,1802
SEL1L	6,70113	7,25714	14,5172	36,7387
CEP170	6,07916	5,66531	14,6897	34,0991
EIF4A3	2,39095	3,31633	7,06897	16,4595
TICAM1	0,872375	1,49388	3,65517	7,75676
RMC1	3,14378	3,20408	5,09483	14,7387
CMIP	19,3635	52,6224	20,5345	119,153
CLPTM1	5,04362	9,25918	4,75862	24,5135
CYSTM1	5,07916	7,8102	13,8276	34,3063
UBE2D3	53,0889	62,6837	64,7155	231,459
RAB27A	15,5267	32,5653	10,7069	75,2973
ORAI3	2,00808	3,59592	15,8103	27,4144
MLX	5,90792	3,86531	2,37069	15,5405
SRPRA	17,3748	17,0837	13,681	61,6036
CAPZB	61,8465	51,3408	33,5776	187,721
ERGIC3	8,3231	5,27959	6,17241	25,2613
CASP10	6,38772	3	3,00862	15,8108
HIPK3	21,3296	21,6633	30,8793	94,0811
CMTM3	20,5347	19,2571	3,59483	55,2432
KIAA1191	5,1454	4,84898	5,08621	19,1982
TMED7	5,08239	8,37755	6,87931	25,8739
YIPF3	6,30533	9,14082	9,12069	31,2252
GPX4	25,7092	24,3367	44,8534	120,577
YPEL3	44,1599	52,0612	61,7586	200,586
DDX60	6,979	6,24694	6,87069	25,4955
METTL9	24,3005	26,0837	13,4224	80,9459
ADI1	6,00808	4,00816	4,31897	18,1802
GPR137	3,20194	3,49796	9,48276	20,5135
TMEM14C	4,94346	4,64694	7,97414	22,2523
SLFN11	2,03069	5,67551	6,26724	17,7027
BROX	7,46365	11,5959	16,5776	45,1441
AC068631.2	1,13247	0,842857	2	5,02703
CCT5	5,19063	7,35714	11,0259	29,8018
ARPC4	10,5412	10,5531	16,319	47,2432
NUP58	4,51535	5,14082	6,11207	19,8919

VAPA	21,1858	21,7429	29,7328	91,3153
DNAJB6	8,99838	18,6408	11,4569	49,0991
GRB2	33,4297	42,5163	71,9569	185,441
TXNDC11	8,15186	9,47143	16,3879	42,6216
KLHL6	9,39418	5,64286	20,3966	44,3874
SH3BGRL3	173,932	153,176	124,431	565,432
ABL2	5,77383	3,23878	6,82759	19,8288
NFYC	5,0727	6,96939	3,02586	18,8559
SZRD1	6,62197	9,9	15,1207	39,5405
SLC30A7	5,07108	10,0163	3,98276	23,7748
VCPIP1	10,8304	13,1755	11,2241	43,9189
MPZL1	1,81583	2,46939	4,30172	10,7027
SH3GLB1	16,1551	24,7551	30,6293	89,1532
PACSIN2	5,65105	4,55306	3,15517	16,6216
TSG101	5,42003	8,30204	9,81034	29,1802
MED13L	13,0646	10,6878	17,069	50,6126
PER1	7,76252	7,58775	10,6207	32,1802
PPP1R18	30,5024	39,8	23,2845	115,937
TMEM33	9,01939	14,998	11,2328	43,6667
BLOC1S2	12,391	16,002	25,3879	66,5045
ATP6V1E1	14,6704	21,898	14,5776	63,2432
ETF1	8,96607	15,8408	14,069	48,0631
POLR2E	5,01292	5,19592	7,50862	21,9009
ARHGEF3	16,8772	14,7714	3,57759	43,5405
SKI	33,727	25,4265	23,3276	101,928
SP110	56,9031	24,0531	67,6207	183,405
GRK3	1,4168	0,677551	11,1379	16,3333
GLE1	3,39903	3,36327	2,9569	11,982
OAZ2	2,75444	4,14082	6,46552	16,4685
ZYX	31,4233	34,5592	13,9138	98,4054
C5orf15	4,53312	4,81633	4,72414	17,3333
GMFB	5,63813	4,23061	9,06034	23,2883
PLEKHB2	8,98061	10,8714	7,46552	33,4955
TMEM179B	4,6559	2,27143	4,06034	13,4505
AC097534.2	7,51858	18,8061	2,52586	35,3063
ARAP1	2,41519	1,74082	2,44828	8,08108
C3orf38	8,09693	8,5	12,5259	35,5856
RNF11	9,48788	13,3939	14,4655	45,5405
FKBP5	107,971	113,029	55,7586	336,919
RAB1B	9,93376	17,6673	21,7759	60,045
TTC7A	2,74313	1,60816	6,62931	13,3333
ZNF281	13,8562	18,5224	12,2155	54,1351
GID8	12,2165	7,4	13,6983	40,4324
SCPEP1	0,980614	1,48163	9,90517	15,009
ADCY7	4,44426	9,34286	2,26724	19,4595
AZIN1	13,8223	20,5837	20,4397	66,2883

ECPAS	11,1922	15,3673	11,8534	46,4234
ZDHHC7	5,81583	15,7612	4,11207	31
ATP10D	0,594507	2,4	8,85345	14,2883
VAMP7	4,78998	6,93061	8,58621	24,4865
SH3GL1	9,38772	7,7449	5,9569	27,8378
CDC73	12,9774	16,0469	22,9138	62,6126
COPE	10,0162	12,5878	7,48276	36,2432
APH1B	1,80291	2,64082	2,38793	8,22523
MSN	54,2246	89,0898	54,9397	238,694
RNF214	1,93053	2,93673	4,43103	11,1802
CSNK2B	10,6462	10,4122	9,66379	36,9279
TBC1D9B	7,14055	5,11224	6,93103	23,0541
MICALL1	1,78191	0,797959	2,63793	6,27027
SLC15A4	3,28271	8,4449	20,75	39,009
GHITM	16,3344	25,7163	18,2586	72,4054
BIN3	3,35218	1,71429	1,81897	8,26126
GRIPAP1	8,50404	6,83265	8,40517	28,4595
RAB8B	13,2423	13,8306	26,8362	64,6126
EDARADD	0,0872375	0,504082	0,137931	0,873874
ARF3	12,7851	11,051	14,1552	45,5135
VPS41	4,32956	4,76939	5,78448	17,8108
TMBIM6	80,42	100,353	130,155	371,243
RCOR1	5,97577	4,25306	6,09483	19,4595
CHCHD2	22,8174	28,7959	28,7672	95,4414
WBP2	14,979	16,8735	16,2414	57,0991
TSPAN3	7,68498	3,43061	12	27,4414
RAB10	16,0614	17,3204	21,3879	65,009
TAGAP	34,1809	32,9429	12,6207	94,6306
SLC38A2	45,0048	43,0163	49,1466	162,568
WTAP	24,7997	19,7286	32,2845	90,8829
USP3	9,6769	13,9816	22,3879	54,4324
BCAP31	16,0307	16,7469	14,069	55,3423
ATF7	5,31825	7,30816	7,32759	23,5586
FPGS	4,29079	3,67347	7,62931	18,4054
TLN1	32,0792	34,6735	56,6121	145,577
PARVG	7,80452	4,58163	7,06897	22,9459
FHOD1	3,42165	3,40612	4,02586	12,7748
FAR1	2,59612	4,53061	8,28448	18,1081
SPPL3	10,2827	7,64286	6,91379	29,1802
STAU1	22,6123	27,4551	12,4052	73,2793
UBXN6	4,53958	3,62449	4,4569	14,8018
CD82	2,2811	6,05918	48,2069	66,2703
ADAM10	14,1357	17,1755	17,1034	56,7297
SELENOS	6,96607	14,2367	8,07759	34,3063
PSMB1	21,0775	22,1571	23,3707	77,9189
RNF5	6,3651	8,99184	5,59483	24,5045

MCRIP1	3,83845	4,63878	5,69828	16,5676
CAPZA1	42,7754	69,598	57,3793	198,333
BMPR2	10,9305	18,6408	26,6983	65,7387
FAM107B	78,9725	61,5184	98,3448	278,775
HUS1	5,95153	5,89592	2,68103	16,955
ZBTB16	3,64136	3,66122	7,27586	17,009
DPH3	8,91115	11,2735	10,25	35,5045
LMO4	4,76575	13,398	6,0431	28,2162
TMEM259	10,7124	10,198	11,8103	38,045
AP2M1	19,7851	18,5551	15,0345	62,045
SRSF9	19,0646	20,5245	21,6552	71,1532
CD2BP2	3,33118	4,07755	3,18103	12,2883
MTCH1	21,5073	23,5857	25,5948	81,964
MKNK1	7,47173	7,96122	14,6293	34,8198
PFKP	11,1809	16,8735	17,9828	53,1622
TMEM109	2,91438	4,92449	3,72414	13,3423
LITAF	22,9144	36,2061	25,0431	97,0811
GLIPR1	12,769	28,8939	30,7759	83,5045
RIPK1	5,67205	5,61837	8,49138	22,7658
RTL8C	9,56704	5,98775	21,8448	43,009
ARL8A	7,31502	6,61224	7,68103	24,8468
CAPNS1	20,811	21,851	33,0948	87,1081
LSM3	8,74798	8,93265	13,0172	35,2883
PDE4DIP	9,90307	9,53061	7,65517	31,1081
B4GALT1	6,85622	18,7449	28,9828	62,6667
AP2A1	3,49596	2,52041	1,81897	8,99099
OSER1	8,83199	6,15918	14,2328	33,5225
TRPC4AP	5,64136	9,56735	9,66379	28,5135
SGK3	4,92569	5,10204	7,99138	20,6486
SMIM14	7,92246	9,74898	23,3276	46,973
TWSG1	1,51212	2,13469	2,00862	6,47748
TMEM268	2,86107	3,39184	5,06897	12,955
EDEM2	2,2391	2,52857	4,63793	10,7568
USP15	23,8433	20,0857	20,1207	73,2342
RASSF3	17,1874	10,8918	15,9138	50,2703
TAF12	9,7609	6,61224	3,59483	22,8018
C9orf72	7,31018	6,38163	9,63793	26,6396
PPP1R11	7,33926	9,27551	6,93966	26,8829
KLF11	2,21325	2,37347	8,50862	14,9279
NECAP1	4,63166	6,88775	7,28448	21,4144
HMG20B	3,94992	7,11224	3,66379	16,7658
PRKACA	11,2795	11,0898	8,40517	34,982
TGFB1	3,36834	13,449	11,5259	32,2162
TMEM131	7,37803	13,8367	13,4138	39,3423
C1D	6,76575	9,76735	9,0431	28,955
CUL1	9,49273	7,23469	11,0086	31,3874

PPP1CA	4,98546	5,29388	12,2586	25,4955
PSMA1	5,08078	5,19184	8,98276	21,7658
TMEM9B	7,7609	14,2714	8,43103	34,3874
MAF1	6,95638	4,51837	7,34483	21,2432
TYK2	9,19709	10,7429	19,1552	44,0991
HLA-F	45,4168	35,8551	45,3879	142,676
UBL3	18,5493	30,798	23,7845	82,3514
NFKBIE	1,13409	1,45714	25,8534	32,009
WASHC2C	5,99677	4,16327	12,1379	25,0631
RHOA	47,9564	59,2184	70,6638	199,748
PELI2	1,87722	3,43061	5,78448	12,4505
PCGF3	4,92084	7,33061	10,0345	25,009
SETD7	1,6252	5,27143	6,13793	14,6216
E2F3	5,00646	4,76735	6,62931	18,3694
H3F3B	114,115	103,706	106,06	362,126
AGO4	3,03231	5,86531	4,42241	14,8649
ANAPC11	4,39903	6,66735	6,47414	19,5495
REEP5	12,3748	19,9714	23,6897	62,4505
BTBD1	17,6753	17,302	12,6983	53,1171
SYPL1	11,6656	8,57143	13,1983	37,2252
DTX2	4,10016	1,91224	5,43103	12,7387
TIPARP	22,147	24,8061	31,569	87,3423
EIF5A	22,5541	23,9673	35,2155	90,8198
SUMO3	15,168	8,69592	17,9224	46,4234
WBP4	2,53796	5,10816	10,6207	20,2883
ENY2	18,9289	17,5551	20,3966	63,1532
DHX34	1,61389	1,53673	5,4569	9,54955
POLR2G	9,89984	11,9367	9,62931	34,8739
RGS19	3,65105	3,59388	5,71552	14,3604
CAMTA1	10,916	10,5408	13,3707	38,5676
MAP2K1	17,5525	22,4184	16,9828	63,0631
RAP2C	7,95638	11,7122	16,3879	39,9189
GINM1	7,01292	4,82041	11,7155	26,045
RGS1	14,4992	54,7878	104,207	191,766
WDFY1	6,81583	8,80408	16,8534	35,8739
MAFG	2,80937	1,92245	8,2931	14,3604
CALCOCO1	9,83199	13,1082	9,0431	35,2432
CSTB	31,0775	32,6061	49,5517	124,649
MECP2	12,0468	15,2082	8,93103	39,8018
ARL5B	8,55897	7,49796	8,52586	27,018
NEAT1	129,695	60,4959	99,2155	318,009
SAMD9	27,895	25,9224	23,1983	84,5946
SURF4	5,95153	17,1796	10,7069	37,1622
FAM91A1	6,27141	6,38163	15,4914	30,8919
TMX1	13,7625	15,4082	22,931	57,1441
MAP2K3	4,39903	5,87143	6,42241	18,2883

NIPSNAP3A	1,83522	1,25306	1,22414	4,72072
KXD1	6,82391	9,58367	8,88793	27,6847
CD59	3,66882	6,45306	12,5345	24,7838
COL4A3BP	16,4103	14,098	11,7069	46,1351
NBN	10,7124	11,3306	26,1983	52,6847
TMEM167A	9,94184	10,1796	19,2586	43
IL10RA	7,4168	13,7837	6,50862	30,2342
CUX1	4,45073	8,38163	11,0517	26,0541
LCOR	15,6042	11,8714	32,2759	65,1712
DEK	48,2876	63,4	67,6293	195,324
LYRM2	8,3958	8,40612	14,3017	33,8378
FKBP8	9,51696	12,3327	6,12931	30,4144
CTDSP2	27,5121	20,6653	27,5517	82,1802
ORMDL2	7,48304	7,11633	17,069	34,3604
CBX3	58,7512	44,6755	55,1983	172,099
SNX14	6,05008	5,50816	6,73276	19,8378
PDE6D	3,89822	4,41224	6,50862	16,0721
SNX13	4,48788	4,97143	3,07759	13,5946
CANX	43,8174	62,6082	51,7069	171,459
IQGAP1	49,9289	92,2735	107,336	270,514
STX5	2,61874	2,47551	11,0862	17,5315
GALC	2,67044	4,94082	5,76724	14,4955
DBI	11,3199	14,0959	17,5086	46,4775
TKT	14,706	9,91429	10,6121	38,1441
NFAT5	9,28271	9,4449	6,52586	27,3243
MAPRE1	12,0727	12,7959	15,3103	43,3604
RAB35	8,47173	15,8898	3,91379	30,5045
GLUD1	11,7641	11,0816	7,19828	32,3784
TANGO2	1,85299	3,07959	2,16379	7,63964
ARNT	0,605816	2,30204	2,59483	5,91892
NRBF2	4,1874	6,5102	9,93103	22,1712
ARL6IP5	43,3263	40,1306	39,6034	132,261
VDAC1	10,874	15,1347	18,4224	47,7117
CCDC47	6,59289	9,45306	8,59483	26,4595
APH1A	10,6543	8,36122	9,42241	30,5315
LASP1	15,3926	22,5429	15,5259	57,3423
SYF2	39,5816	42,0755	36,4052	126,631
SIPA1L1	4,64459	12,4429	22,569	42,4775
DYNLL1	6,47334	10,4531	14,569	33,7027
ECHDC1	8,93861	8,48367	6,28448	25,3604
CD164	36,6349	32,9347	42,6121	119,982
LAMTOR4	15,3199	10,1265	17,8362	46,2342
FNIP1	6,55089	8,4	8,21552	24,7297
SETD3	11,8158	11,8816	6,69828	32,4144
SQLE	2,06462	2,60816	9,75	15,3784
POLD3	6,21809	5,3551	3,12069	15,6396

MAPKAPK2	9,85299	14,0224	10,3276	36,3874
RYBP	22,0291	19,5347	32,4397	78,7297
TMEM165	3,51858	5,15918	4,86207	14,3964
CCNK	13,6866	22,4102	19,4052	58,8829
TMED9	11,4685	17,402	18,3448	50,0901
RBX1	12,8498	12,7673	7,08621	34,6937
RAB2A	23,5202	21,1837	48,6724	99,018
ATP2B1	9,69144	10,4735	21,4138	44,0631
PEF1	3,64782	2,4551	3,00862	9,64865
NT5C2	13,7302	16,0102	12,9914	45,2072
SNX2	5,57027	8,69796	27,5172	44,1351
PTPN18	7,80614	12,9102	24	47,1441
BLOC1S1	4,13893	4,09796	8,76724	17,9099
NSF	1,55735	9,90816	10,6897	23,2703
EIF4H	50,4168	53,9837	43,2414	154,964
TP53BP2	12,4168	12,6878	11,2155	38
GPR183	42,5961	42,851	74,2328	166,468
DYNLT3	9,67044	7,79592	8,96552	27,5496
PTPN1	21,0985	21,7857	46,3707	92,991
WSB2	1,50081	3,8	2,59483	8,22523
MANBA	7,22617	6,08571	21,681	36,4505
CFL1	176,05	170,51	227,241	597,036
LSP1	9,35218	20,7653	29,431	61,9279
LRRFIP2	5,68336	8,87347	6,37931	21,7658
RER1	14,1922	13,2245	9,73276	38,5495
TRIM5	3,95315	5,96326	4,65517	15,0991
HMGB2	61,4507	97,4571	40,5431	206,604
CHIC2	9,78514	16,349	8,06897	35,3514
INSIG1	7,74636	7,4	36,1207	52,973
RBM23	11,4152	8,7551	22,6379	44,1982
ROMO1	8,05331	10,9735	14,4138	34,4865
GMIP	2,7496	2,4	4,64655	10,0991
ATP1A1	25,1519	18,7673	14,3534	60,009
FAM32A	7,08401	8,17755	6,84483	22,7387
ASNA1	1,13247	3,15714	2,59483	7,07207
COQ10B	12,3489	12,0633	19,8276	45,3874
LAMTOR5	8,13247	8,58367	12,2586	29,6937
DNPEP	4,32795	4,2449	8,03448	17,018
MLLT1	8,40226	9,90816	8,30172	27,2523
NUBP1	1,80937	0,795918	4,34483	7,11712
RSU1	15,454	12,898	7,5431	36,7297
PAIP2	31,0565	33,1694	28,1897	94,5315
SCFD1	6,12439	6,82245	4,12931	17,4595
TAF10	9,3651	5,76122	10,8621	26,5676
ARPC2	88,2601	99,0388	83,1121	276,252
SLC36A4	6,62036	3,98367	7,18103	18,1532

TM9SF3	16,7851	22,2633	26,5862	66,955
PCYT1A	4,31987	5,01633	3,67241	13,2523
ZBTB7B	1,4378	1,87551	2,14655	5,55856
COX17	6,06785	10,3673	12,681	29,6126
STK40	2,3021	7,03469	5,07759	14,6577
ATP5PD	39,2423	38,4531	28,9914	108,459
SNRPB2	17,3393	23,1224	16,4397	57,8018
CDIPT	9,38611	9,70408	10,1121	29,6577
CAPN1	6,08239	6,57755	11,2845	24,3063
TMSB4X	490,9	542,871	322,991	1376,6
TOR1B	3,40226	0,544898	2,31034	6,34234
CRK	4,33441	4,02245	6,56034	15,1081
KLF6	236,462	343,873	552,862	1146,53
COX6B1	22,9031	20,7469	27,5431	72,009
KCNAB2	27,2746	23,7408	10,4828	62,1982
ARL6IP1	24,6074	28,9857	29,5603	84,0721
SEMA4D	28,2213	17,3286	27,2672	73,5586
SHISA5	27,0226	24,6327	26,6897	79,0721
SSR1	30,4055	34,8408	29,319	95,4324
FMNL1	20,1826	28,2061	37,5345	86,6667
CHD9	13,7431	15,3551	24,0948	53,5856
RSRC1	6,17932	10,1469	8,90517	25,4054
PPP6C	18,4426	19,8388	15,2672	53,8919
DDX3X	40,4669	39,8633	64,2931	145,396
COPA	21,475	24,398	27,5776	73,8198
HPS1	5,81099	6,14082	11,7759	23,8378
FAM120A	27,5557	29,5449	44,181	101,495
RHBDD2	13,0178	14,0551	4,59483	31,7027
HADHA	28,0711	24,4327	16,5259	69,0991
GLTP	5,50404	9,39184	12,4052	27,3153
GTF2I	33,0678	37,0286	52,7672	122,811
TMBIM4	5,55573	2,53673	2,94828	11,036
PSMF1	13,4766	16,4673	15,2845	45,1351
ARL6IP6	4,88368	4,55306	11,2069	20,5946
SKIL	41,231	53,5714	128,103	222,288
MAN1A1	3,063	7,59388	9,84483	20,4054
LAMTOR1	5,84814	6,73265	6,92241	19,3964
ACTB	204,68	145,345	313,784	660,108
NPEPPS	17,8918	17,9755	24,7241	60,2342
KIF13B	5,83199	16,0633	9,62931	31,3333
AKT1	11,8869	16,2714	13,6293	41,4955
STX4	6,47657	4,16531	4,89655	15,4234
NDUFB4	17,5751	19,1224	16,8103	53,0631
MYL12B	54,6171	68,0775	40,5431	161,676
EPN1	7,46688	6,32857	9,58621	23,1532
EIF5	39,021	38,9469	53,9828	130,649

PSMA3-AS1	12,6058	8,5	8,83621	29,6306
TSPAN14	46,3667	34,6694	13,7759	93,8108
FLII	5,20517	6,76531	6,2069	17,982
GSK3A	9,23102	14,8388	17,681	41,2703
GGA1	9,27787	12,0143	14,3276	35,2072
COPB2	12,9208	12,5041	15,1293	40,0721
CRB1	0,124394	0,14898	0,146552	0,414414
AMD1	11,748	11,5449	18,6121	41,3514
FADS3	0,371567	2,38775	5,60345	8,25225
ZNF706	17,4669	11,5755	22,4914	50,8018
RARA	3,43296	7,56326	20,431	30,9369
BCL7B	12,0743	8,03061	20,1897	39,5676
ANKFY1	6,37157	5,06939	11,4138	22,4324
HIPK2	9,36187	5,48775	21,9655	36,1261
REEP4	3,2811	7,02653	2,78448	12,8468
TNFRSF14	3,8433	2,1551	7,62931	13,3694
OGFR	2,44426	3,18571	2,62931	8,0991
CCDC71L	2,58158	2,97959	3	8,38739
YIPF5	8,57189	20,0429	12,9914	40,6216
OSTF1	4,18417	8,43061	9,4569	21,5405
HIGD2A	5,97738	5,10612	9,12069	19,6757
SPCS3	21,8788	34,6673	13,7069	68,3784
RELA	35,9047	33,0306	29,7328	95,9279
GSDMD	9,03231	6,26735	10,6034	25,1712
SNAP23	14,0226	15,7429	19,9397	48,2432
PHKB	3,20517	8,28571	6,77586	17,7207
CARD8-AS1	3,38126	2,6449	11,6121	17,0901
PRR13	26,3877	30,9694	36,819	91,2162
SFPQ	47,0452	49,5755	62,8534	154,459
LIMK1	2,39418	2,78163	7,09483	11,8829
RALY	10,7496	10,7143	13,7328	34,0811
DCAF6	4,73829	5,65918	10,4052	20,1171
ZFAND6	10,357	13,2408	19,3793	41,5586
TAB2	21,6931	14,6469	24,0517	58,3694
NAGK	4,021	3,82653	5,87069	13,2523
KCTD5	1,958	3,23469	4,77586	9,62162
ARAF	7,91761	7,32245	10,1207	24,4595
ATP5F1C	14,8805	9,11429	14,8276	37,3874
VOPP1	6,44103	6,57143	34,5948	45,8288
PCBP2	118,662	104,712	135,741	345,613
RAB11B	28,8433	34,2041	56,2241	114,712
COX8A	14,2084	15,1653	18,0259	45,5135
RNF40	6,95477	11,6367	12,0517	29,4054
APOPT1	1,12278	3	3,03448	6,85586
RAD23B	12,3796	25,3122	12,319	47,9009
NAF1	4,22617	3,63061	9,41379	16,5405

GALNT1	19,2973	19,551	27,819	63,7928
BLM	1,77383	3,5551	3,63793	8,57658
TAOK1	16,504	19,3347	21,569	54,8919
SKP1	30,7544	27,6959	28,8276	83,4414
HIPK1	23,2859	17,849	11,9828	50,7748
SERF2	40,5784	40,9102	46,7931	122,604
EIF4E3	10,9095	10,2327	4,74138	24,7027
ARF4	12,4378	18,5898	14,0517	43,009
PDXK	3,62036	7,93673	3,71552	14,5676
PGAM1	7,89822	15,4143	21,3966	42,6396
GDI2	22,0468	31,5878	49,8707	98,5946
HNRNPH2	7,48304	10,3429	7,67241	24,2432
ACAP2	22,7981	28,198	25,819	72,8468
UBE2J1	7,98546	8,84286	11,2931	26,6486
TRIM26	7,15993	8,17755	7,13793	21,2793
CCNL1	50,0695	43,7143	73,4828	157,955
TGIF1	14,2504	23,7306	11,9224	47,1081
ELMSAN1	8,8433	8,09184	11,9741	27,2883
STT3B	18,6107	20,4735	17,25	53,1261
COPG1	10,0614	9,9	12,2241	30,3514
TXNL1	11,1502	11,3694	7,68966	28,4775
ZFP36	159,084	170,394	364,293	653,901
UBQLN1	15,7108	13,1265	14,9224	41,2342
PXK	5,98061	11,7367	17,6207	33,2883
ACBD5	4,70598	12,1041	15,3103	30,2523
STX7	2,23263	4,03469	12,4397	17,5946
FGD5-AS1	16,6155	12,8429	11,7328	38,7387
ARFIP1	2,36187	4,93878	7,99138	14,3694
TMEM8A	2,76737	4,13673	7,50862	13,5405
BTF3L4	6,99838	8,11837	11,6379	25,1261
RNFT1	2,52504	3,11429	4,62069	9,63063
SNN	6,4475	0,728571	7,56897	13,8378
XRN1	29,9806	26,6245	37,8621	88,5946
ATP6V1G1	21,7964	20,949	24,931	63,4054
SUMO1	9,6559	13,8633	10,181	31,5676
MORF4L1	60,9628	60,9612	80,3966	189,198
NXF1	13,378	16,9082	18,7586	45,8378
ING3	11,3344	10,8224	16,3448	35,982
WWP2	4,06139	7,57551	7,85345	18,1892
TM2D1	5,38934	2,95102	8,68103	15,8739
TNFAIP3	203,226	378,29	143,095	674,901
CIB1	14,1405	15,2224	21,25	47,0631
TES	34,2456	34,4653	39,1724	100,315
SUMO2	46,0711	53,7388	46,9741	136,306
SMC1A	10,0646	9,56735	14,7069	31,8829
GPSM3	22,4976	11,0388	10,2069	40,6126

MARK2	17,2908	18,4224	22,6034	54,0991
NRBP1	16,7577	17,0755	15,6466	45,9009
UGP2	16,6058	17,798	8	39,2883
C6orf47	3,16801	5,5	4,32759	11,964
DNAJC13	10,7561	13,3367	12,0948	33,3063
LGALS8	27,6042	43,5918	10,0259	74,7117
KLF9	51,8288	47,1796	20,2931	109,604
SAP18	37,8142	36,1918	36,8707	101,829
EFTUD2	5,06139	6,42653	5,13793	15,2613
MICU1	5,29402	4,69592	13,2672	21,3423
DYNC1I2	19,3813	11,198	20,2155	46,5135
BMP2K	4,87237	7,14898	21,2931	30,4865
RIT1	7,24556	10,502	5,0431	20,8378
TBC1D22A	5,16478	8,21224	10,8966	22,1712
SSB	36,6155	38,5306	39,0086	104,153
DOCK8	38,7011	32,1204	41	102
BZW1	31,3635	38,4816	34,6034	95,2703
MTDH	52,0985	59,9184	54,569	151,856
HEXA	2,88045	3,63673	11,7672	16,6577
DCTN4	4,41195	3,62245	7,93966	14,4955
PNPLA8	10,5024	11,0408	19,0172	36,7928
TNFAIP8	36,0969	42,4429	56,6121	122,135
FAM111A	11,2197	7,9102	10,8276	27,045
ZNF787	3,09531	3,96327	5,75862	11,5496
USP16	9,61389	14,9673	5,35345	26,973
PTPN2	18,7286	13,6694	23,1983	50,027
GNB1	58,1002	59,4898	70,5	169,234
GNA13	19,4265	20,1102	51,1638	81,5856
WASHC2A	7,20032	6,95306	6,89655	18,9009
TAX1BP1	38,6462	47,1653	40,75	113,631
EP300	25,273	30,6816	33	79,8018
CLSTN1	7,25202	8,18571	10,1552	22,955
USF3	9,78837	7,24694	15,9914	29,6126
CAPRIN1	5,25848	10,0286	8,31897	21,1441
SEC61G	15,8094	20,8224	21,4828	52,009
PPM1B	6,27464	10,1551	6,03448	20,0901
SF3B4	22,5719	42,1612	35,569	89,6937
CHMP2B	14,748	13,7837	17,5	41,1532
UBE2K	23,7658	23,0857	22,819	62,1982
SP100	68,622	78,9755	94,8103	216,378
DNM2	15,832	19,698	19,5345	49
RALBP1	17,391	20,3	25,2328	55,955
SAMD8	5,57835	4,12857	12,2931	19,5495
TIMM10	7,33764	3,14082	4,62931	13,4054
DEDD2	15,4701	14,7796	27,7155	51,4144
SNAPIN	4,15024	5,3102	8,06034	15,5315

PSMC1	8,04039	9,7551	3,57759	18,9369
PIM3	8,91115	5,86531	21,8103	32,4054
OTUB1	10,3393	11,7265	4,75	23,7477
KIF5B	24,9257	35,7531	43,9741	92,6667
NDUFA2	10,5897	13,5816	19,9483	39,0541
PPP2R1A	9,24233	9,86122	15,7155	30,8018
UBE2A	8,90469	19,3571	17,3534	40,3423
PHF11	40,0969	35,6653	46,1207	107,703
SELENOT	12,084	18,5429	32,2759	55,5495
NPC1	1,12763	5,90204	5,16379	10,7658
PJA2	13,231	16,2327	19,6983	43,2342
UBL5	12,9693	18,0653	23,3793	47,8198
VPS4B	21,399	26,8327	20,6638	60,4685
NFKBIB	5,88691	6,16122	9,26724	18,7027
PSMB4	30,6171	29,6306	48,5948	95,3063
RLIM	9,73021	6,44286	17,1121	29,1081
PAFAH1B1	26,7593	30,7653	30,6207	77,045
SSR3	22,294	32,0673	26,8276	70,9369
ROCK1	34,3619	40,5	31,3534	92,7568
VPS39	7,10339	7,01224	8,74138	19,9279
HOOK3	20,5703	17,2184	21,7845	51,8919
CCNI	159,578	145,686	143,112	390,369
SLAMF7	0,168013	3,4551	7,96552	10,0811
RHOB	2,16801	3,73673	9,19828	13,1171
DYNLRB1	22,4588	31,5653	29,931	72,5946
HNRNPF	34,727	30,2041	21,431	74,5586
SLIRP	11,0129	10,3286	18,8707	34,7117
CERS2	22,7738	32,0571	22,6897	66,9009
ATP5F1E	180,013	162,604	186,75	456,82
ACSL5	3,21486	2,96327	4,98276	9,63063
NDUFB9	9,04039	8,28367	5,2069	19,4414
YPEL5	39,1486	63,3673	95,3276	170,495
UBE2L3	22,1292	34,0857	43,0862	85,4865
ISG20L2	15,105	18,1245	16,2414	42,5315
RB1CC1	25,1842	32,1694	21,569	67,8468
PQLC1	6,18094	3,32245	10,5517	17,2072
MKRN1	13,9015	19,8082	24,2845	49,7117
DHRS7	16,9176	36,8531	24,8362	67,2793
TRAM1	50,769	64,5898	67,4138	156,198
CLIP1	15,0549	15,7469	19,569	43,045
CHMP1A	6,71244	12,0122	9,74138	24,2973
PPTC7	12,7916	17,0959	24,1207	46,0721
RNF10	15,9128	20,7837	23,319	51,1622
PRXL2C	4,70275	5,63673	5,81897	13,7658
ADAR	38,0048	37,898	44,6293	102,559
DICER1	12,0291	11,5429	20,9655	37,8739

CCT8	12,8562	16,849	13,1379	36,4054
SERINC3	24,8239	32,9245	22,5862	68,2072
HNRNPK	67,2698	77,3143	101,802	209,18
IFI16	50,7157	84,4408	56,4741	162,64
GSK3B	13,8207	21,0755	18,0259	44,8468
NUS1	8,97254	17,3735	18	37,5586
LINC02227	0,140549	0,144898	0,172414	0,387387
SSU72	32,2488	22,7775	26,25	68,6577
TBC1D5	8,15832	22,8408	19,1724	42,3514
JARID2	7,09693	13,3245	19,6466	33,8018
ATP5MPL	21,1551	19,3408	14,4397	46,2793
RRBP1	5,04039	7,79184	9,63793	18,9279
RPS6KA1	1,8126	4,64286	3,42241	8,31532
IRF2	16,8918	18,6816	13,9397	41,6757
CYBC1	11,1309	8,73878	26,7759	39,1351
RNF114	11,1486	10,1204	18,5259	33,2973
EIF2S2	27,4798	32,0837	31,1207	75,8649
TGOLN2	47,063	43,6918	42,7672	111,667
VTI1A	4,36026	6,25714	5,00862	13,0631
SLC50A1	6,13247	7,0551	7,59483	17,3514
TRIP12	17,1147	17,2265	22,3621	47,3333
FBXO7	18,7771	23,2143	16,0431	48,3964
PPP4R2	14,378	19,3163	26,681	50,2883
PNPLA2	26,273	29,5816	35,2414	75,8649
NCBP3	15,3102	22,5776	29,3621	55,955
ATP5PF	15,7318	17,3531	23,431	47,009
PSMG2	13,9305	12,1449	21,4483	39,4414
SP140L	16,0598	13,3694	28,569	48,0811
TRIM44	21,2278	10,4673	38,7586	58,3964
RHEB	4,36672	4,8102	3,25	10,2973
LARP7	15,3231	18,6163	23,1293	47,2523
DNAJC7	8,89984	9,41633	8,85345	22,4955
WIPF2	13,643	26,7306	26,9569	55,7207
CLASRP	6,58643	7,98367	8,62069	19,1892
MTRF1L	5,89661	6,4449	11,2845	19,5405
HBP1	19,2536	25,3245	34,0431	64,9369
YME1L1	35,441	33,4061	21,6293	74,6937
H2AFZ	29,6333	25,1551	29,4052	69,4505
C6orf62	54,3409	44,2347	48,1034	120,901
MESD	14,9548	13,6306	15,5086	36,2793
RERE	14,8223	8,83673	26,9741	41,6577
SYNGR2	6,7609	7,05306	40,3362	44,5405
PIP5K1C	7,72698	7,46122	8,63793	19,5946
ARHGDIA	22,7561	23,6939	19,0172	53,8288
BECN1	22,3958	23,6531	37,2155	68,4234
ELL2	7,87884	19,8939	57,5948	70,027

CNPY3	6,78675	4,55714	8,26724	16,0811
PSMA3	12,5412	15,1918	22,0517	40,8108
LCP1	40,5267	72,9265	70,8362	151,063
HDAC2	9,10985	6,17959	10,25	20,9099
UBALD2	13,1357	6,22449	15,569	28,5856
ARID3A	2,18417	4,11429	8,25	11,8829
DBET	0,387722	0,536735	0,896552	1,48649
C19orf66	42,9822	29,7796	48,6034	99,027
TPM3	85,2439	98,6775	100,31	231,604
DNAJC8	21,517	24,6592	24,569	57,6306
MAT2A	8,15832	5,8898	6,93966	17,0811
FOXN2	16,9241	23,1837	8,69828	39,7207
WIPF1	51,7593	77,2449	46,25	142,477
ATP5MF	18,4879	15,5082	18,4483	42,6036
CCDC6	7,47657	13,0082	33,7328	44,027
SRP54	12,4927	10,8918	13,9483	30,2973
NDUFB1	14,9176	12,3959	13,1897	32,8559
HSD17B4	3,51858	3,49796	11,1552	14,7387
SEC22B	12,378	12,698	11,681	29,8108
ATP5MG	17,1486	17,6184	5,43966	32,6036
HSP90AA1	99,8013	122,112	142,164	295,099
CDC37	21,6963	14,6367	14,4655	41,1441
NUAK2	5,56381	0,391837	8,49138	11,6847
MLF2	18,9903	18,0449	21,0776	46,991
FKBP15	9,23263	7,76327	18,8966	29,009
ARFGAP3	12,8675	18,0429	25,6638	45,7207
PPP1R3D	1,79321	1,64694	4,99138	6,8018
HNRNPA2B1	126,565	93,9061	147,31	296,459
PPP2CA	18,1567	21,8061	19,0086	47,5135
CBWD2	1,26817	1,67551	4,7069	6,16216
KIAA0040	3,42811	2,21837	10,5776	13,0631
ELK3	26,3796	48,5837	48,3362	99,1712
FUS	74,4863	55,8347	81,319	170
JAK1	98,5913	72,7959	58,8017	184,811
CLTC	8,73344	9,14082	21,3362	31,4054
STAT6	19,3312	12,4878	48,5172	64,3153
HECA	38,4733	33,0959	23,1897	75,8378
SUN2	46,2682	45,8714	24,8621	93,5766
MBD6	5,40872	5,6449	10,8534	17,4685
EIF4G2	67,8401	82,9755	103,595	202,342
CSNK1D	23,7205	29,2939	19,9828	57,9459
PRPF3	12,3877	7,13061	10,3966	23,7387
CD99	69,7431	106,667	44,2672	175,009
UFM1	29,4362	35,3796	26,1293	72,027
SLK	18,1373	21,1184	19,0948	46,2072
SP1	27,9031	14,1224	18,3793	47,8108

RBM22	20,1228	22,9204	24,5776	53,4955
CSDE1	79,3506	63,5857	80,3707	176,541
UBXN4	38,1761	43,7755	21,5603	81,8108
GABARAPL2	12,4297	17,9082	29,1121	46,982
LMAN2	7,64782	10,6327	10,9138	23,036
RAP1B	28,7544	33,1286	20,6121	65,0901
VPS35	16,6139	18,2898	16,0431	40,0901
ADIPOR2	4,92569	10,1673	10,0172	19,7477
SLC3A2	23,3312	25,4939	31,0776	62,8288
USP9X	15,8998	16,2939	19,5517	40,6847
CTDNEP1	13,6947	12,702	15,8448	33,1892
GSTK1	31,1325	18,8898	20,8966	55,6937
BLOC1S6	16,8174	10,2469	28,5086	43,6216
VDAC2	21,5121	22,4694	15,0603	46,2432
ARF6	60,7641	89,4245	131,207	220,207
HSPA1A	2,32795	15,6755	10,1466	22,027
RALGDS	4,19709	6,54898	10,3362	16,4955
NFKB1	3,94346	8,47347	11,7328	18,8649
MAPK1IP1L	19,5719	27,4673	33,2672	62,6937
CSNK1A1	37,2359	39,9694	38,0259	89,8649
ZFP36L1	106,344	104,814	230,638	344,441
COX6A1	19,7367	23,7714	21,7241	50,7838
ABI1	17,8756	15,451	18,931	40,6487
RAPGEF1	9,35056	12,7286	32,2845	42,2703
WIPI2	12,8546	14,7918	14,6207	32,8559
TRIM38	17,7884	17,3939	25,9052	47,4324
EFCAB14	24,3796	32,351	28,7586	66,2072
MOB3A	7,12439	13,3673	34,3103	42,3153
EWSR1	24,4523	33,4204	35,4741	72,027
BRWD3	4,81422	5,73265	7,5431	13,955
POLR1D	50,3683	48,7327	47,5603	113,126
DNAJA2	21,8191	15,6592	18,8276	43,4054
BAG6	5,29725	4,0551	12,4741	16,8198
SLC25A6	33,8304	28,1	48,1983	84,7568
SPAG7	3,63005	4,32857	3,85345	9,09009
CREB3L2	1,92892	3,61429	7,75862	10,2072
PTMS	0,369952	4,64082	6,2931	8,66667
PFDN5	108,969	95,2531	89,6897	225,288
GDI1	21,7754	21,4531	28,931	55,2703
SNX6	23,3522	29,2082	37,1466	68,6577
DCTD	7,70759	8,11429	12,5172	21,6757
RRAGC	9,64136	8,23673	29,3017	35,991
SPCS2	18,6397	24,4816	29,0345	55,027
AKIRIN2	7,63813	10,9102	19,8793	29,2973
DCUN1D1	11,0113	8,6102	7,32759	20,4144
TCF25	27,4265	25,3388	42,181	71,9099

SDF4	8,52666	16,0143	18,3103	32,4505
AHCYL1	10,7189	8,76326	22,5776	31,8468
BCAP29	4,01131	4,43265	16,7328	19,027
ATF4	57,1195	58,1633	59,069	131,667
LYST	6,8853	20,0796	18,9397	34,6216
TIMMDC1	7,37157	7,69592	6,81897	16,5045
YWHAB	120,937	120,351	122,75	274,225
CCDC50	0,455573	3,52041	22,4828	19,9279
GADD45B	17,8158	11,4327	49,2155	59,036
FMNL3	7,28918	5,90408	19,9655	24,8829
RAB21	15,8837	12,3	21,931	37,5856
CHMP1B	20,5299	21,1102	32,2155	55,1171
MED13	12,3683	21,0286	19,681	39,5946
PTBP3	57,6204	57,9653	69,7328	138,108
CSRNP1	5,57351	8,74082	14,8879	21,7477
PPP1R15B	12,7108	13,402	17,8448	32,7027
MFSD10	5,40549	9,83877	10,5948	19,2162
ZNF207	36,4701	32,6776	43,3534	83,5586
PABPN1	24,6074	21,1775	32,9138	58,3153
KHDRBS1	48,2278	40,3408	46,7586	100,027
VPS28	9,14378	9,12245	13,931	23,7838
SERP1	39,3118	38,4061	45,1466	90,7477
SH3BGRL	14,8158	15,2082	34,6983	47,7027
ELOB	12,3005	17,9	15,3362	33,5495
CREBRF	59,1583	45,7939	36,2586	104,036
ECH1	8,35703	9,24082	7,63793	18,5676
GALNT2	4,57835	8,15714	39,1207	38,1441
FMR1	12,0307	10,502	15,3017	27,8108
TRAF3	3,42811	5,41633	21,1379	22
PRDM1	14,5864	69,3898	4,84483	65,1532
SYT11	2,01939	11,4347	1,63793	11,027
MBD4	16,3732	17,3571	23,569	41,8559
GNA12	4,29887	4,25306	48,0431	41,2973
ZNHIT1	11,1018	13,5449	18,2155	31,2703
UBE3A	19,1486	20,6286	35,9483	55,2162
NSFL1C	7,03393	9,1449	10	19,0811
UBE2B	22,3635	24,2184	28,6379	54,7477
WHRN	1,48142	12,049	5,66379	13,955
TMEM258	30,9305	33,502	31,5172	69,6937
STK17B	115,152	131,49	158,328	293,838
OXA1L	29,2116	23,9265	30,9828	61
TMCO1	13,0388	14,4	17,1466	32,3153
H6PD	5,76898	2,86735	12,5517	15,3514
UBAP1	5,25202	8,72245	10,0259	17,3694
YBX1	124,756	121,31	231,216	345,261
MAGT1	8,57351	9,88571	11,069	21,3333

RB1	9,00162	7,92041	36,7155	38,7477
DLGAP4	4,91115	4,89796	20,5603	21,9009
QSER1	3,77221	4,74082	5,26724	9,91892
LARP1	16,126	10,5388	15,5862	30,3694
DOCK2	12,9402	12,8837	19,2155	32,3063
PAK2	43,4798	41,2551	57,9397	102,315
OSBPL11	9,08724	12,3633	18,1552	28,3784
DAPP1	1,38288	1,05714	11,2586	9,81081
ADPGK	9,08562	12,3571	22,8362	31,7027
PPIA	103,764	97,2939	134	239,748
AATF	9,07431	7,55306	12,6897	20,964
SRP14	40,7447	44,7775	55,4828	100,748
SASH3	3,36026	3,33265	11,8276	13,2252
CTBP1	15,2359	15,9612	21,3448	37,4685
CHTF18	0,819063	1,41224	4,68103	4,92793
PPP3CA	13,916	27,0184	34,681	53,8378
CCNDBP1	16,2666	21,3918	25,4741	44,8739
PRDX1	5,979	5,9449	14,2759	18,5676
DCP2	13,4588	13,0531	13,5345	28,3694
SON	100,969	85,498	132,293	225,649
USP28	1,94023	9,62857	3,74138	10,8198
PTGER4	12,5024	29,3224	11,9655	37,973
NELFE	4,69144	2,50408	8,17241	10,8468
TUBA1B	19,0097	16,6551	35,2931	50,0721
MALAT1	1269,19	775,445	1258,43	2326,13
PRKCSH	17,6058	19,849	24,6121	43,6577
KTN1	64,5751	76,2694	87,7155	160,757
PRPF40A	42,0856	48,7959	50,2759	99,2793
CDV3	38,7706	38,1143	50,2069	89,1261
KARS	15,4976	13,451	22,2069	35,8468
NFKB2	4,59935	3,27143	25,2759	23,2162
XRCC5	45,9128	53,1592	43,9052	100,099
PTBP1	36,021	45,0633	64,0086	101,135
CLPTM1L	8,74475	9,40408	11,6983	20,7658
CFLAR	34,2003	75,1347	107,655	150,676
PTGES3	39,2294	54,0796	30,681	86,018
EIF3H	85,8966	60,5796	73,6034	152,36
DCAF7	10,7318	17,4041	18,4741	32,2342
DIAPH1	23,5073	25,7694	17,9138	46,4595
VHL	12,8982	19,5735	40,1466	50,1802
DBNL	14,0145	5,66122	14,5086	23,6036
RPN2	19,7932	24,502	15,9397	41,4955
N4BP1	22,0565	25,398	25	49,9099
NTAN1	4,50565	6,9102	11,5948	15,8378
MAP7D1	19,601	17,3449	17,75	37,6396
ZDHHC3	9,41195	10,5347	9,47414	20,1712

TMED2	17,8481	24,9408	18,931	42,2883
CDC42SE1	69,958	63,0796	33,7069	113,946
N4BP2L2	44,5024	41,2388	40,4397	86,2252
CBWD1	0,864297	1,87143	6,86207	6,55856
BRK1	21,9063	22,6224	33,6034	52,9279
RNPS1	22,0129	26,3959	35,0259	56,3514
USP12	11,5622	11,3653	21,8879	30,2613
DUSP10	2,67044	31,5796	44,2845	52,7568
PAPOLA	50,609	50,8224	59,6638	108,162
SEC61B	5,55573	13,3918	14,3448	22,3063
EIF6	4,00485	5,57347	10,2931	13,2793
DNAJC1	11,7609	26,6837	19,8621	38,8919
CALR	34,7383	47,0408	101,276	122,018
TMA7	55,4588	68,2388	42,1552	110,36
TMED10	27,8885	34,298	47,569	73,018
NBPF14	3,92246	3,05102	5,42241	8,24324
STARD4	1,96769	3,89388	10,5603	10,9189
APMAP	7	19,3204	8,36207	23,0541
UTRN	12,9806	27,3755	22,3966	41,5315
RNF41	8,6769	10,7245	19,7155	25,8829
CHSY1	4,92892	6,15918	18,6034	19,6396
KIDINS220	17,546	20,102	13,8621	34,045
IFITM2	22,7512	29,2388	24,3448	50,1892
ELOVL1	4,16801	4,2	9,03448	11,4414
UBE2Q1	26,1632	22,2653	33,4052	53,7838
AP3S1	13,7011	21,8041	36,8621	47,5045
EIF3I	18,6543	17,7857	23,0345	39,027
TAOK3	28,1357	29,9469	29,5948	57,3153
IMPAD1	9,07108	14,4959	15,5948	25,5225
SELENOF	20,7011	27,1551	36,5172	54,955
NCALD	0,285945	0,606122	0,146552	0,675676
IDS	55,4782	73,9	91,3362	143,514
PRRC2C	113,616	93,3898	81,1552	187,216
PCGF5	16,4168	22,2878	33,0948	46,4775
PFDN1	10,3199	7,98163	14,9914	21,5315
NCKAP1L	11,7157	16,9878	21,2328	32,2703
TRIM8	21,0565	14,3061	15,6207	32,8829
SEM1	12,2407	15,8122	19,6293	30,7387
YWHAQ	21,7076	34,149	25,0517	52,1171
RPS24	481,635	358,494	380,328	785,892
U2AF2	38,441	36,0408	38,25	72,5496
MT-ND1	2625,86	2769,49	3070,75	5447,93
YWHAZ	173,721	215,635	314,388	452,414
HNRNPM	36,9144	40,4673	47,4224	80,1351
BAX	13,7496	13,1898	10,75	24,1712
ATP5F1B	13,6446	18,8184	11,9052	28,2432

FNDC3A	6,14378	4,07143	7,03448	10,9459
ATP5MD	18,7383	22,4245	22,75	40,4054
COPZ1	17,3457	21,4796	30,2328	43,6306
SEC14L1	12,9063	16,4122	21,4569	31,955
SMIM29	3,2601	4,87959	7,75	9,99099
MGAT4A	17,0662	20,4939	1,76724	24,7207
TUFM	12,895	16,7735	16,8966	29,2252
SCAMP2	15,8627	16,1592	24,8017	35,6577
GUCD1	2,56058	2,25918	10,2845	9,47748
SLC35E1	8,15832	13,0776	21,6897	26,8018
ZFAS1	155,044	157	169,905	300,892
PEAK1	3,19063	1,92653	8,57759	8,54955
ELF1	47,2714	46,7857	49,9828	89,8829
TPT1	2537,13	1974,14	1567,32	3792,11
CNBP	42,9111	48,9327	43,9397	84,6667
BAZ2A	19,1212	15,9755	25,7845	37,955
COA3	2,13732	1,97551	12,931	10,5766
LPGAT1	5,69467	25,9449	21,9828	33,2072
LRWD1	2,67205	2,92449	13,8707	11,964
MYO5A	4,43296	10,7041	18,1121	20,3784
MAPK1	20,1632	28,2939	18,6207	41,018
KCMF1	13,1438	18,202	31,1379	38,0991
SRRM2	79,8304	82,5755	119,422	171,234
RBM3	34,9919	26,4286	55,2328	70,8468
ACADVL	18,0759	17,5714	52,8621	53,7207
EMC4	10,273	13,1408	18,4741	25,3694
EIF1B	13,546	14,9551	36,7931	39,3694
NDUFA1	46,1632	53,6755	54,9741	92,982
EIF2S3	43,8417	26,4245	45,0345	69,2162
DRAM2	5,80291	4,40816	19,1552	17,6216
RRM2B	4,50242	4,05306	24,7069	19,8378
BIRC2	19,9935	13,8959	19,8103	31,9369
UBE2I	27,8708	23,9327	31,5	49,4865
RAPGEF2	5,79483	11,1367	18,1724	20,7838
PDIA6	9,57674	13,6939	48,1207	42,1441
PMEPA1	11,3522	17,9673	48,681	46,027
SEC31A	24,1147	26,8327	40,1638	53,7207
BTG3	2,31987	5,52245	5,87931	8,09009
RNF167	12,6624	13,3755	12,3534	22,6306
HNRNPU	76,3716	72,4204	66,9224	127,099
MT-CO1	6160,12	6883,64	8076,45	12395,4
PPFIA1	11,4055	9,65918	15,6724	21,4144
NR4A2	1,23586	5,43265	11,6724	10,6847
WASHC4	14,496	14,0633	31,9224	35,1351
GTF3C1	2,35703	6,51837	1,06034	5,76577
FEM1C	6,26979	8,92449	16,6379	18,4685

SNRPB	8,03231	13,5653	21,3362	24,8378
SRP9	17,1922	17,4837	18,9655	30,8468
CD2AP	15,189	14,8551	23,75	30,9279
TMEM208	3,03393	2,33265	7,31897	7,27928
EIF4EBP2	19,6656	26,5673	23,0517	39,6396
KDM5C	16,525	20,551	20,7328	33,045
ISG20	10,231	13,9469	31,7672	31,964
RPS9	98,3118	73,9878	86,9138	147,964
KIF1B	3,43619	5,98571	3,85345	7,55856
COX4I1	49,5638	34,6531	55,6983	79,5225
ACTG1	87,0792	64,6388	95,0517	139,45
SND1	10,1809	10,7878	10,9914	18,045
CEP104	7,5525	4,07755	7,30172	10,6216
UBB	34,2649	36,5612	63,3707	75,2883
UBA52	232,955	203,069	192,155	349,712
GMEB1	3,46527	4,41837	5,93966	7,68468
SHOC2	24,4863	24,9469	36,3707	47,3243
IRF2BP2	56,3845	38,4755	76,6034	94,5225
RSRP1	34,6931	30,1306	51,6638	64,2072
JUND	110,934	110,949	441,664	364,45
NHSL2	12,1502	12,9122	8,7931	18,5946
PRPF4B	33,7997	36,8633	51,9052	66,5856
POLR2A	20,5703	14,1918	36,4138	38,3874
PPDPF	45,5816	34,2327	58,3534	74,1892
TMCC3	0,0113086	5,30816	1,91379	3,88288
ARHGDIB	111,399	99,5816	63,9052	146,252
PHTF2	2,93538	3,84286	6,59483	7,10811
SMAD7	9,52181	28,5041	28,4569	35,0631
CREBBP	27,1179	25,002	45,181	51,1802
RPL8	185,394	165,512	186,483	281,739
MLXIP	13,7044	6,77755	22,6466	22,4414
LENG8	53,3635	37,0265	55,0603	75,5676
HNRNPL	31,0921	30,849	38,3534	51,7477
MIEN1	2,99192	4,14898	12,4052	10,0811
RBM39	119,924	121,404	154,534	204,144
PRKCE	1,6979	1,71224	11,2069	7,52252
TSC22D3	130,845	155,908	165,578	232,73
EIF4G3	7,0517	13,8041	26,6121	24,2432
THRAP3	29,8029	30,0102	54,0862	58,009
CCDC82	14,8998	14,6041	31,6983	30,8739
PDE4B	9,09208	20,6694	54,8879	42,6126
ANKRD13A	7,47657	8,30612	20,7759	18,3964
REPIN1	5,57027	3,01633	15,4655	12,0811
KDM4B	4,97738	5,22653	17,5603	13,7117
AP1G2	26,3037	14,0204	33,6121	36,4324
HSP90B1	45,0905	77,6612	78,4741	97,8829

NAA50	13,1002	36,051	17,5259	32,1712
PPP2R5A	18,6204	32,1959	5,43103	27,1261
KDM6B	10,9176	10,0327	47,0603	32,7117
BTG1	226,044	228,731	373,914	394,982
HERPUD2	22,9192	40,2449	35,569	45,9369
APOBEC3G	2,97577	33,6388	20,1121	26,2793
TMEM123	136,62	59,2041	216,802	190,811
CXCR4	347,428	594,2	1023,66	898,748
HNRNPUL1	40,0905	71,349	71,0948	83,2252
ZBTB10	8,063	6,33469	18,9914	15,1261
SPINT2	7,45073	2,64898	24,1724	15,4505
COPB1	12,6236	20,5673	27,6897	27,4414
GNAS	158,898	170,522	279,94	272,928
PTMA	697,158	717,122	1299,74	1212,34
ICOSLG	3,63166	1,18571	12,8017	7,75676
RNF115	9,65751	23,5653	9,37931	18,6036
MYH9	77,1131	132,004	80,1897	125,973
NCOA3	24,7173	28,7347	59,9914	49,0811
APOBEC3C	7,42326	31,8816	39,7672	33,955
EEF1A1	2061,75	1726,71	1826,86	2377,27
HMGB1	130,247	172,035	262,164	237,279
CIRBP	86,0808	60,1286	98,6638	101,829
RACK1	223,772	164,865	203,966	243,793
HERPUD1	23,1761	49,2122	99,1552	70,2703
SYS1	11,1115	10,1918	24,6724	18,7297
LPCAT1	3,13086	7,67347	9,12069	8,09009
DDIT3	5,07916	5,35102	22,2672	13,2252
RPS11	310,414	273,461	373,957	384,225
SLA	26,1502	53,498	9,81897	35,009
ZFP36L2	93,6801	154,947	173,655	162,441
PMAIP1	3,17447	19,3612	31,3534	20,7207
IQGAP2	26,4507	50,4755	6,06897	31,1171
PPP1R14B	8,69144	21,3735	15,5259	16,5315
SLC1A4	0,00969305	5,32857	3,93103	3,35135
JAZF1	1,34249	5,07551	8,31034	5,23423
MT-ND5	1876,29	2167,96	2444,13	2235,01
MRPL10	8,52666	23,2265	3,48276	12,0541
LYAR	9,26333	29,7327	6,34483	15,4865
SMARCA2	40,8546	43,2653	93,5862	60,5676
SPRY1	0,996769	4,92041	4,87931	3,57658
EDEM1	10,2439	10,202	19,8793	13,2523
MT-ND3	4190,52	3701,41	4358,1	3990,9
ZNF791	12,2068	9,06122	34	17,955
PIP4K2A	35,7367	79,9326	12,7069	38,5135
PRDM2	21,3457	22,898	59,2672	29,991
KDM2B	4,5832	11,2857	18,8707	9,94595

ARPC5L	12,42	29,5163	10,9914	15,0721
SLC9A8	1,82068	3,76122	8,58621	3,67568
CBLB	16,3279	26,0102	12,0259	13,9099
ARRDC2	46,2132	17,8429	82,8707	36,4324
SMAD5	4,94346	15,8653	16,9569	9,2973
ZBED5	25,3796	11,0755	41,4138	18,6126
GGA2	5,14863	19,1959	32,5	13,5856
TTC39C	9,77544	21,2327	4,02586	8,27928
TERF2IP	38,9548	70,6388	43,7586	34,964
CHST12	2,20678	8,70816	6,30172	3,90991
EBLN3P	29,1018	15,849	58,8966	23,2613
LRR1	1,96446	2,46122	2,62069	1,51351
PLEKHA2	22,6688	74,5612	99,8966	40,982
RUNX3	62,748	134,52	90,5172	57,9099
PRMT1	7,77544	9,64082	25,4483	8,54054
GNPTAB	9,73506	23,1816	41,6983	13,8288
CCSER2	29,9015	64,0673	18,3621	20,2342
ZBTB38	18,7625	38,0163	15,569	11,1081
HSP90AB1	106,459	91,6143	213,302	61,1441
DDI2	8,65105	21,2735	14,8966	6,54054
YARS	6,96931	14,9673	6,33621	3,65766
GNG2	19,538	48,7551	29,2241	12,2973
RNF19A	21,1712	75,9714	66,4741	20,2613
SNX9	34,0792	24,7	72,5172	15,3874
ARAP2	16,7011	29,6429	33,3448	8,85586
PEBP1	36,7577	42,8347	59,4828	9,52252
DUSP4	0,667205	27,8551	24,6724	3,48649
NFATC2	7,84491	16,8245	11,2759	2,10811
LINC00910	0,271405	0,477551	0,258621	0
